# Elucidation of Hepatitis C Virus Transmission and Early Diversification by Single Genome Sequencing

**DOI:** 10.1371/journal.ppat.1002880

**Published:** 2012-08-23

**Authors:** Hui Li, Mark B. Stoddard, Shuyi Wang, Lily M. Blair, Elena E. Giorgi, Erica H. Parrish, Gerald H. Learn, Peter Hraber, Paul A. Goepfert, Michael S. Saag, Thomas N. Denny, Barton F. Haynes, Beatrice H. Hahn, Ruy M. Ribeiro, Alan S. Perelson, Bette T. Korber, Tanmoy Bhattacharya, George M. Shaw

**Affiliations:** 1 Perelman School of Medicine, University of Pennsylvania, Philadelphia, Pennsylvania, United States of America; 2 Santa Fe Institute, Santa Fe, New Mexico, United States of America; 3 Theoretical Division, Los Alamos National Laboratory, Los Alamos, New Mexico, United States of America; 4 Center for Nonlinear Studies, Los Alamos National Laboratory, Los Alamos, New Mexico, United States of America; 5 University of Alabama at Birmingham, Birmingham, Alabama, United States of America; 6 Duke University School of Medicine, Durham, North Carolina, United States of America; University of Texas at Austin, United States of America

## Abstract

A precise molecular identification of transmitted hepatitis C virus (HCV) genomes could illuminate key aspects of transmission biology, immunopathogenesis and natural history. We used single genome sequencing of 2,922 half or quarter genomes from plasma viral RNA to identify transmitted/founder (T/F) viruses in 17 subjects with acute community-acquired HCV infection. Sequences from 13 of 17 acute subjects, but none of 14 chronic controls, exhibited one or more discrete low diversity viral lineages. Sequences within each lineage generally revealed a star-like phylogeny of mutations that coalesced to unambiguous T/F viral genomes. Numbers of transmitted viruses leading to productive clinical infection were estimated to range from 1 to 37 or more (median = 4). Four acutely infected subjects showed a distinctly different pattern of virus diversity that deviated from a star-like phylogeny. In these cases, empirical analysis and mathematical modeling suggested high multiplicity virus transmission from individuals who themselves were acutely infected or had experienced a virus population bottleneck due to antiviral drug therapy. These results provide new quantitative and qualitative insights into HCV transmission, revealing for the first time virus-host interactions that successful vaccines or treatment interventions will need to overcome. Our findings further suggest a novel experimental strategy for identifying full-length T/F genomes for proteome-wide analyses of HCV biology and adaptation to antiviral drug or immune pressures.

## Author Summary

Hepatitis C virus infects as many as 170 million people worldwide. Globally, there are seven major genotypes of HCV that differ by approximately 30% in nucleotide sequence. Importantly, the natural history of HCV infection is variable, ranging from spontaneous resolution to persistent viremia and chronic disease. Factors responsible for this variability in clinical outcome are unknown but likely involve a combination of viral and host determinants. To this end, a precise molecular identification of transmitted HCV genomes could illuminate key aspects of transmission biology, immunopathogenesis and natural history. We used single genome sequencing of plasma viral RNA to identify transmitted viral genomes and their progeny in 17 subjects with acute infection. Numbers of transmitted viruses leading to productive clinical infection ranged from 1 to 37 or more (median = 4). Surprisingly, we found evidence of high multiplicity acute-to-acute HCV transmission in 3 of 17 subjects, which suggests that clinical transmission of HCV, like that of HIV-1, may be enhanced in early infection when virus titers are highest and neutralizing antibodies are absent. These results provide novel insight into HCV transmission and early virus diversification key to our understanding of virus natural history and response to drug selection and immune pressure.

## Introduction

Hepatitis C virus (HCV) infects as many as 170 million people or nearly 3% of the world's population. The virus causes a wide variety of pathologic outcomes, the most significant being chronic liver disease, cirrhosis and hepatocellular carcinoma, which is nearly always fatal. HCV infection is the leading indication for liver transplantation in the United States [1].

HCV is a positive strand, non-segmented, enveloped RNA virus of approximately 9.6 kb in length. The virus is classified in the genus *Hepacivirus* within the larger family of *Flavivirus*, which includes the human pathogens West Nile virus, yellow fever virus and dengue fever virus among others [2]. A common feature among the *Flaviviridae* is their dependence on a virally-encoded RNA-dependent RNA polymerase (RdRp) for replication [3]. RdRp is error-prone, and HCV is notable for its extensive diversity within and among individuals. Globally, there are seven major genotypes of HCV that differ by approximately 30% in nucleotide sequence [1], [4], [5].

The extraordinary diversity of HCV complicates studies of virus biology, pathogenesis and susceptibility to novel therapeutics. Clinically, the different HCV genotypes exhibit variable natural history and responsiveness to interferon, ribavirin and the newer direct acting antiviral (DAA) agents [2], [6], [7]. HCV variation poses similar challenges to the development of effective vaccines and to the elucidation of viral immunopathogenesis [5], [8]–[10]. It is of interest then that the extraordinary diversity of HCV is similar to that of HIV-1 and that a novel experimental strategy to identify transmitted/founder (T/F) HIV-1 genomes has led to new insights into virus transmission and persistence [11]–[18].

Acute HCV infection isconventionally defined as the initial 6 months of infection and sets into motion virus-host interactions that to a large extent dictate the natural history of the disease [9], [10], [19]–[30]. Depending on viral genotype and host immunogenetic factors, most importantly IL28B alleles, a proportion of newly infected individuals spontaneously controls or eliminates virus [9], [10], [31]–[33]. A greater number can be cured if the infection is treated with interferon and ribavirin alone or in combination with DAA drugs [6], [34]–[36]. Mechanistically, how this occurs is incompletely understood. From a vaccine perspective, the acute infection period is critical, since transmitted viruses are the obvious targets of a vaccine and early stages of infection when viral diversity is lowest represent a period when the virus may be most vulnerable to elimination by vaccine-elicited immune responses [26], [37]. For all these reasons, there is considerable interest in the molecular features of the initial virus population ‘bottleneck’ associated with virus transmission and the subsequent pathways of virus evolution that lead to persistence [19]–[25], [28], [29], [38], [39].

Previous reports have described different experimental approaches to the analysis of the HCV transmission bottleneck. These include studies that employed a DNA heteroduplex gel shift method to estimate viral diversity [39], [40], conventional polymerase chain reaction (PCR) methods to bulk amplify, sequence, or clone and sequence fragments of HCV genomes [20], [21], [23], [25], [28], [38], [41], [42], and 454 pyrosequencing to interrogate early viral sequences more deeply but narrowly [19], [43]. These reports documented a restriction in viral diversity associated with virus transmission, but despite the use of increasingly sensitive methods, a precise quantitative, molecular description of HCV transmission and early diversification has remained elusive. In the current study, we hypothesized that T/F HCV genomes could be identified unambiguously and their early pathways of diversification mapped precisely by single genome amplification (SGA) followed by direct amplicon sequencing, otherwise known as single genome sequencing [17], an approach we used previously to gain insight into HIV-1 transmission [15], [18]. This strategy differs from previous methods applied to HCV by providing gene-wide or genome-wide viral sequences that are proportional to their representation in human plasma and are not confounded by template resampling or by *Taq*polymerase errors of nucleotide misincorporation or recombination [15], [17], [18], [44]–[47]. We amplified and sequenced HCV core, E1, E2, P7, NS2 and NS3 genes and analyzed them by adapting a model of random HIV-1 evolution [15], [48], [49] to account for differences in the biology of replication between the HIV-1 and HCV. In an accompanying report, Ribeiro and colleagues [50] used these sequences together with plasma viral load kinetic data to develop an agent based stochastic model of acute HCV replication dynamics resulting in new estimates of the HCV mutation rate in humans.

## Results

### Study subjects, viral load measurements and single genome sequencing

**Figure 1:**
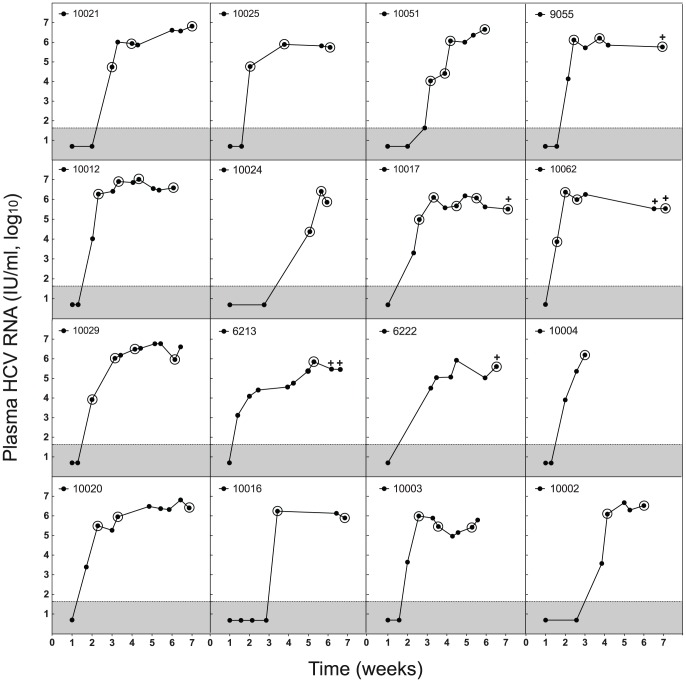
HCV plasma viral RNA kinetics in acute infection subjects. PlasmavRNA was quantified by Roche COBAS AmpliPrep/Taqman HCV assay. Circled values indicate samples subjected to vRNA sequencing and plus signs denote HCV antibody positivity.

One hundred fifty-four plasma specimens from 17 subjects with acute HCV infection and 14 subjects with chronic HCV infection were analyzed for viral RNA (vRNA) load (Figure 1; Table S1). Acute infection subjects were source plasma donors who had undergone once or twice weekly plasmapheresis for months, and in some cases years, with persistently negative testing for HCV, HBV and HIV antibody or RNA before becoming acutely infected by HCV. Chronically infected subjects were patients at the University of Alabama at Birmingham who were known to have been HCV infected for approximately 3 to >20 years. All subjects were untreated with anti-HCV medications. A median of 8 (range 5–11) sequential specimens per acutely infected subject was analyzed for vRNA load spanning the period of plasma vRNA negativity through exponential increase to an early plateau (Figure 1), and a subset of these was analyzed for sequence diversity. The median peak plasma viral load in acute infection subjects was 2,850,000 IU/ml (range = 527,000–10,300,000 IU/ml). Five of 17 acutely infected subjects developed HCV antibodies by the last sampling time point. Chronic subjects had a median vRNA load of 2,081,138 IU/ml (range = 24,000–7,690,001 IU/ml), all were HCV antibody positive, and all were sampled once for vRNA sequence diversity. A total of 2003 5′ half genomes and 919 5′ quarter genomes were amplified, sequenced, and recorded (Genbank accession numbers JQ801756–JQ804520; JX178293–178443).

### Phylogenetic analysis of acute and chronic sequences

**Figure 2:**
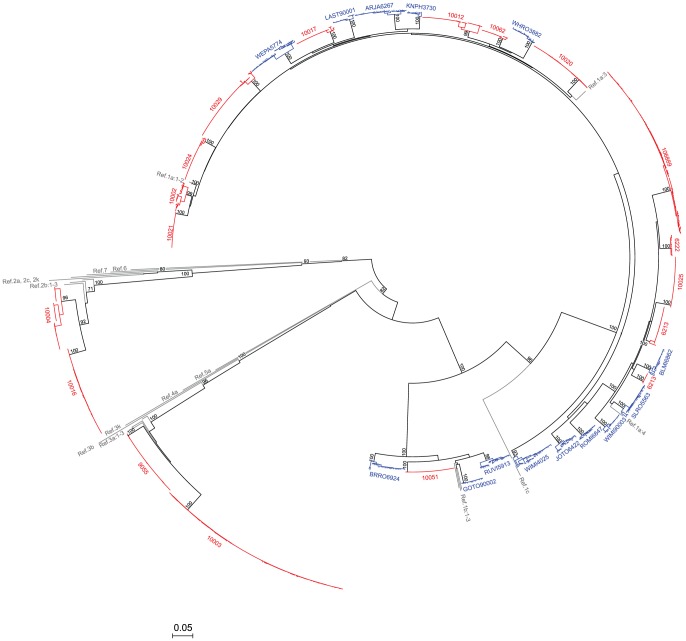
Maximum-likelihood tree (ML) of HCV sequences from acute and chronic infection subjects. 5′ quarter 1 genomesequences (*core*, *E1*, *E2*) from acute (red) and chronic (blue) subjects are shown along with HCV genotype 1 to 7 reference sequences(gray). Bootstrap values represent 100 repetitions in this and subsequent figures. The horizontal scale bar indicates 5% diversity.

Sequences corresponding to core antigen and envelope E1 and E2 genes (2.2 kb) from the initial HCV RNA positive sample from acute and chronic subjects were subjected to maximum-likelihood (ML) phylogenetic analysis (Figure 2). Sequences formed subject-specific clades (bootstraps 93–100%) and represented HCV genotypes 1a (n = 23), 1b (n = 4), 2b (n = 2) or 3a (n = 2). No subject was infected by more than one virus genotype and there was no intermixing of sequences between subjects in the phylogenetic tree. Sequences from acute and chronic subjects revealedwidely varying degrees of maximum within-subject diversity ranging from 0.14% to 6.40% (Tables 1 and S1), which was not different between the two groups (p>0.05, Mann-Whitney test). Acute sequences were, however, distinctly different from chronic sequences in having one or more discrete lineages characterized by extremely low diversity. This was true for sequences from each acutely infected subject but from none of the chronically infected subjects. Maximum diversity of sequences within these discrete viral lineages from acute subjects (mean 0.12%; median 0.12%; range 0.04–0.19%) was significantly lower than the overall diversity observed within chronic subjects (mean 2.27%; median 2.37%; range 0.56–3.83%; p<0.0001, unpaired T-test with Welch's correction) (Figure S1).

**Table 1:** Diversity and mutation analyses of HCV sequences in acute infection.

Sample ID	Sample date	Genotype	Genome^a^	Maximum nt length of viral genome analyzed	Total number of sequences analyzed	Number of transmitted/founder viruses	Maximum nucleotide sequence diversity (%)	Transmitted/founder lineage analyzed	Total number of sequences within lineage	Maximum intra-lineage diversity (%)
10021	1998	1a	5q1, 5q2, 5h	4878	151	1	0.14	v1	151	0.14
10051	1998	1b	5q1, 5q2, 5h	4863	303	1	0.19	v1	303	0.19
10025	1994	1a	5q1, 5q2, 5h	4881	175	1	0.16	v1	175	0.16
10024	1994	1a	5q1, 5q2, 5h	4881	222	6	1.35	v1	212	0.12
10012	1998	1a	5q1, 5q2, 5h	4881	230	3	3.34	v1	96	0.12
								v2	96	0.14
								v3	38	0.12
10029	1998	1a	5q1, 5q2, 5h	4875	322	9	3.08	v1	241	0.18
								v2	19	0.10
								v5	18	0.06
								v6	11	0.04
								v8	13	0.12
10062	1996	1a	5q1, 5q2, 5h	4857	188	3	0.80	v1	163	0.16
								v2	20	0.06
10017	1993	1a	5q1, 5q2, 5h	4887	249	4	1.39	v1	220	0.16
								v2	6	0.06
6213	1992	1a	5h	4905	41	3	6.40	v1	31	0.14
								v3	9	0.08
6222	1992	1a	5h	4902	17	4	0.59	v1	7	0.10
10020	1998	1a	5q1, 5h	4905	122	10	0.27	all	122	N/A^g^
								06.QB18^f^	27	0.12
10002	1994	1a	5h	4905	31	13	3.12	v5	5	0.08
10004	1998	2b	5q1	2802	36	3	4.39	v1	16	0.11
								v2	11	0.11
								v3	9	0.14
10016	1998	2b	5q1	2804	72	15	0.36	all	72	N/A
								10.QE4^f^	10	0.18
9055	1998	3a	5h	4911	157	1	0.18	v1	157	0.18
10003	1998	3a	5h	4911	133	37	0.41	all	134	N/A
								07NA5^f^	10	0.14
								07B13^f^	11	0.10
106889	2008	1a	5h	4902	87	>30	1.02	all	87	N/A
								5.B.F9^f^	10	0.06
								5.02C22^f^	7	0.10
Average^h^										
Median					151	4	0.80			0.12

Sample ID	Insertion rate^b^	Deletion rate^b^	Stop codon rate^b^	CT/TC transition rate^b^	AG/GA transition rate^b^	Trans-version rate^b^	Substitution rate^b^	dN/dS^b^	Number of sequences of 1st sampled timepoint^c^	Possion GOF_PVAL^c^	Star-like phylogeny^c^
10021	0.000000	0.000002	0.000003	0.000079	0.000052	0.000007	0.000137	0.326129	31/24	0.735/0.562	Y/Y
10051	0.000003	0.000002	0.000001	0.000056	0.000035	0.000007	0.000098	0.557751	46/45	0.237/0.601	Y/Y
10025	0.000002	0.000003	0.000000	0.000089	0.000075	0.000018	0.000182	0.687757	40/43	0.758/0.270	Y/Y
10024	0.000001	0.000004	0.000000	0.000063	0.000028	0.000008	0.000098	0.414301	37/68	0.390/0.898	Y/Y
10012	0.000000	0.000000	0.000000	0.000061	0.000061	0.000019	0.000141	0.322564	13/26	0.867/0.890	Y/Y
	0.000000	0.000006	0.000000	0.000075	0.000054	0.000015	0.000143	0.307586	34/20	0.916/0.870	Y/Y
	0.000006	0.000000	0.000006	0.000115	0.000048	0.000012	0.000175	0.166389	5/3	0.794/0.563	Y/Y
10029	0.000000	0.000002	0.000000	0.000083	0.000057	0.000016	0.000156	0.413231	55/38	0.584/0.283	Y/Y
	0.000000	0.000000	0.000000	0.000096	0.000055	0.000000	0.000151	0.401201	4^d^	0.59	Y
	0.000000	0.000000	0.000000	0.000050	0.000037	0.000012	0.000099	0.166901	4^d^	0.59	Y
	0.000015	0.000015	0.000000	0.000075	0.000015	0.000000	0.000090	0.067286	5^d^	0.612	Y
	0.000000	0.000000	0.000000	0.000132	0.000066	0.000016	0.000214	0.534788	4^d^	0.64	Y
10062	0.000001	0.000000	0.000000	0.000046	0.000051	0.000019	0.000116	0.356217	19/19	0.801/0.598	Y/Y
	0.000000	0.000000	0.000000	0.000024	0.000036	0.000012	0.000073	1.665695	5^d^	0.852	Y
10017	0.000003	0.000009	0.000001	0.000078	0.000062	0.000016	0.000156	0.428199	30/21	0.536/0.879	Y/Y
	0.000000	0.000000	0.000042	0.000166	0.000083	0.000000	0.000250	0.332046	2/0	-e/-e	-e/-e
6213	0.000000	0.000033	0.000000	0.000118	0.000039	0.000026	0.000184	0.238436	31	0.93	Y
	0.000000	0.000000	0.000000	0.000113	0.000045	0.000023	0.000181	0.329702	9	0.668	Y
6222	0.000000	0.000000	0.000000	0.000058	0.000117	0.000117	0.000291	0.774175	7	0.753	Y
10020	N/A	N/A	N/A	N/A	N/A	N/A	N/A	N/A	57	0.0494	N
	0.000000	0.000000	0.000000	0.000060	0.000048	0.000012	0.000120	0.148288	18	0.65	Y
10002	0.000000	0.000000	0.000000	0.000122	0.000163	0.000000	0.000285	0.249986	5	0.232	Y
10004	0.000000	0.000000	0.000000	0.000156	0.000022	0.000000	0.000178	0.039859	16	0.65	Y
	0.000000	0.000000	0.000000	0.000065	0.000032	0.000000	0.000097	0.639207	11	0.662	Y
	0.000000	0.000000	0.000000	0.000119	0.000119	0.000000	0.000238	1.593102	9	0.837	Y
10016	N/A	N/A	N/A	N/A	N/A	N/A	N/A	N/A	53	0.001	N
	0.000000	0.000000	0.000000	0.000214	0.000071	0.000036	0.000321	0.253774	8	0.873	Y
9055	0.000000	0.000001	0.000000	0.000082	0.000061	0.000019	0.000162	0.407277	44	0.23	Y
10003	N/A	N/A	N/A	N/A	N/A	N/A	N/A	N/A	121	0	N
	0.000000	0.000020	0.000000	0.000102	0.000143	0.000081	0.000326	0.468484	7	0.166	Y
	0.000000	0.000000	0.000000	0.000074	0.000130	0.000000	0.000204	0.273291	10	0.94	Y
106889	N/A	N/A	N/A	N/A	N/A	N/A	N/A	N/A	87	0	N
	0.000000	0.000000	0.000000	0.000061	0.000000	0.000041	0.000102	0.220469	10	0.815	Y
	0.000000	0.000000	0.000000	0.000146	0.000087	0.000000	0.000233	0.198278	7	0.875	Y
Average^h^	0.000001	0.000003	0.000001	0.000075	0.000053	0.000014	0.000143	0.392939			
Median											

a 5h - 5′ half genome contains core, E1, E2, p7, NS2, and NS3; 5q1- 5′ quarter 1 genome contains core, E1, E2, p7 and partial NS2; 5q2 - 5′ quarter 2 genome contains partial NS2 and NS3.

b Rate calculations were derived from sequences from each discrete transmitted/founder lineage.

c Sequences from 1st sampled time point with ≥4 sequences per lineage were analyzed. If 5′ half genomes were not available, quarter genomes 1 and 2 were analyzed with each result shown.

d Sequences from 2nd sampled time point were analyzed due to insufficient number of sequences or sequence diversity from 1st sampled time point.

e Insufficient numbers of sequences from each time point to calculate fit to Poisson or star-like phylogeny.

f Transmitted/founder lineage identified by this sequence in respective ML tree and *Highlighter* plot.

g N/A, not applicable due to multiple transmitted/founder virus genomes.

h Averages were calculated from total mutations in all transmitted/founder lineages from all subjects combined. Because of low numbers of sequences and mutations in some lineages, certain values (e.g. dN/dS for subjects 10062 v2 and 10004 v3) vary substantially from the mean.

### Mathematical models of early HCV diversification

**Figure 3:**
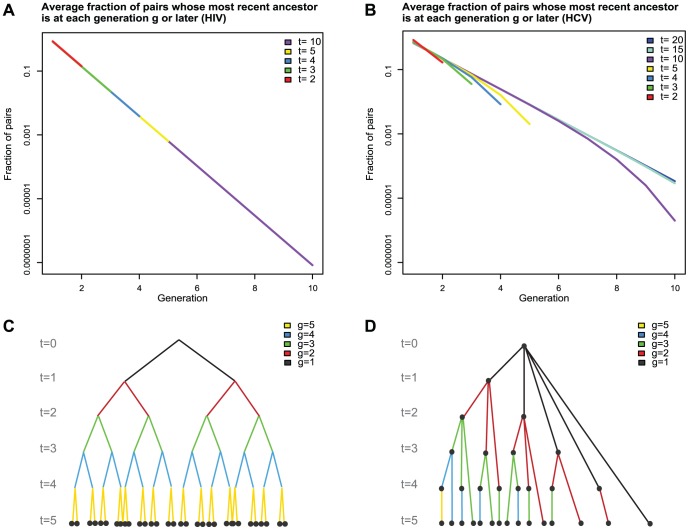
Models of HCV and HIV-1 diversification in acute infection. Panels A and C depict patterns of HIV-1 replication and diversification compared with HCV in panels B and D. The various generations of HIV-1 (panel C) and HCV (panel D) are indicated as filled dots to represent HIV-1 proviruses or HCV replication complexes. The symbol *t* indicates the time to sampling in units of provirus generations for HIV-1 (∼2 days) and of replication complex generations for HCV (∼1 day). Whereas HIV-1 is produced only from the latest generation of provirus, HCV is produced from replication complexes of all generations. In this model, the number of HCV replication complexes of generation *g* at time *t* is given by 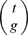. For HIV-1 each provirus of the same generation produces roughly equal numbers of descendant HIV viruses. The replication complexes of HCV produce widely different number of descendant viruses. Panels A and B show the impact of this difference on the expected fraction of pairs of viruses, sampled at various times, that share a given number of ancestors. For HCV, the number of pairs of viruses sampled at time *t* that share at least *g* generations is given by 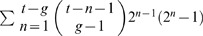. One sees that HCV viruses typically share 50% more generations of ancestors than HIV, and with the estimated mutation rates, this means that they share about 3 times the stochastic mutation events expected in HIV.

Because of the distinctive replication strategy of HCV, which is still not fully understood [3], [5], we could not predict *a priori* the patterns of early virus diversification that we might observe in acutely infected subjects. Thus, we developed two mathematical models to analyze HCV sequence diversity (Figure 3). The first model [15], [48], [49], employed previously to analyze early HIV-1 sequence diversification, assumes a narrow genetic bottleneck associated with virus transmission, initial rapid exponential virus growth, constant lineage-independent mutation rates at all sites, no recombination between sequences or back mutations, and no differential selection. When diversity is low, most base substitutions occur at distinct loci, pairwise differences between sequences (i.e., Hamming distances, HD) follow a Poisson distribution, and sequences exhibit a star-like phylogeny and coalesce to distinct unambiguous T/F genomes [15], [18], [51]. Early HIV-1 diversification conforms well to this model and it was suggested that other viruses including HCV might also [48]. However, HCV replication differs from HIV-1 in that HCV RNA does not integrate into chromosomal DNA and it does not produce all of its daughter progeny in a large burst of viruses within a couple days after infecting a cell [52]. Instead, it forms as many as 40 cytosolic replication complexes that continue to produce virions throughout the lifetime of the cell [50], [53]. In early infection prior to the onset of HCV specific cellular immune responses, the lifetime of infectedcells is likely to span our sampling period given that the lifetime of uninfected hepatocytes is estimated to be months to years [54], [55]. To account for these differences with HIV-1, we developed an alternative simplified deterministic model of HCV diversification (Figure 3). This model predicts occasional violations in star-like diversification and in the Poisson fit of mutations with increasing probability as time goes on, and it predicts greater numbers of shared stochastic mutations between HCV sequences compared with HIV-1 sequences. The latter model provided us with a mathematical and statistical basis for distinguishing closely related T/F HCV lineages from sequences that evolved from a single genome but shared early stochastic mutations. Importantly, it allowed us to vary key assumptions regarding the contributions of long-lived hepatocytes containing multiple generations of replication complexes and assess the effects on sample-based T/F virus enumeration. From this analysis, we adopted a conservative operational cut-off of >2 shared mutations per quarter genome (∼2500 bp) or >4 shared mutations per half genome (∼5000 bp) to distinguish T/F genomes in most cases of HCV transmission from chronically infected individuals (see Methods). In more complicated transmission scenarios, where the index case was hypothesized to be either acutely infected or to have experienced a viral genetic bottleneck due to antiviral drug therapy, we applied both an empirical approach where a single shared polymorphism could represent a distinct T/F viral genome as well as the more conservative modeling approaches. Importantly, both empirical and model-based analyses predicted that consensus sequences of low diversity lineages before the onset of immune-driven positive selection coalesced to founder viral genomes at or near the moment of transmission.

### Identification and enumeration of T/F genomes

**Figure 4:**
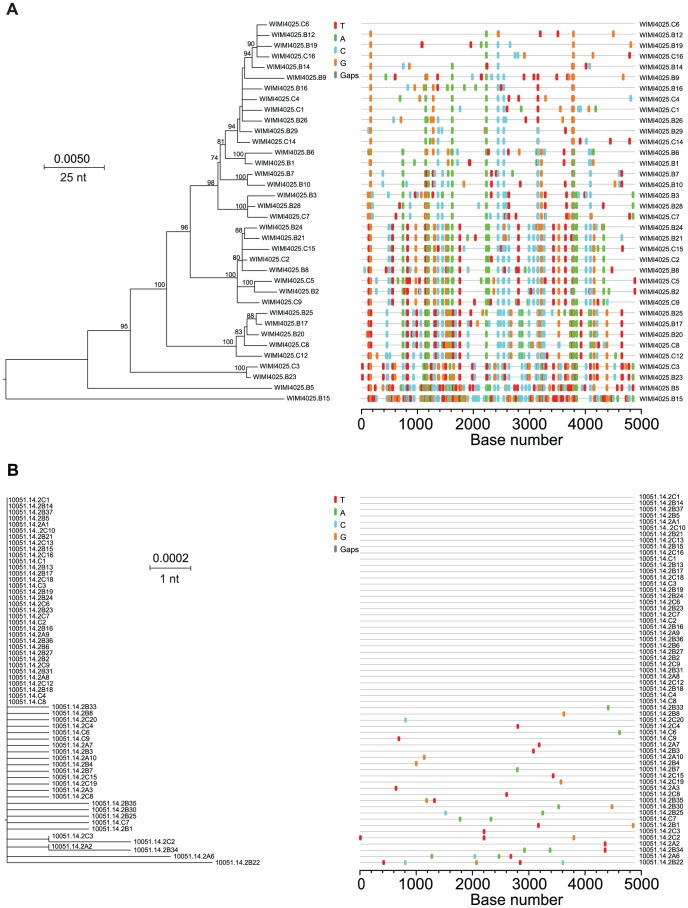
HCV diversity in chronic (WIMI4025)and acute (10051) subjects. 5′ half genome sequences (*core*, *E1*, *E2*, *p7*, *NS2* and *NS3*) from WIMI4025 (A) and 10051 (B) are depicted by mid-point rooted ML phylogenies and *Highlighter* plots.

Figure 4 depicts ML phylogenetic trees and *Highlighter* plots of viral 5′ half genome sequences from two subjects, one typical of chronic infection (WIMI4025, panel A) and one illustrating the reduced genetic diversity characteristic of a subject productively infected by a single viral genome (10051, panel B). Sequences from the chronic subject showed broad genotypic heterogeneity with a maximum inter-sequence diversity of 3.83% (mean 1.2%; median 1.21%; range 0.12–3.83%) typical of chronic infection (Table S1). Sequences from the acute subject revealed a very different pattern of diversification (Figure 4B). These sequences, which were derived from the last sampled time point 21 days after the beginning of documented viremia (see Figure 1), were extremely homogeneous with a mean diversity of 0.03%, median diversity of 0.02%, and a range in diversity of 0–0.19%. Nucleotide substitutions corresponded to a near star-like phylogeny, although unlike most early HIV-1 sequence sets they deviated from a Poisson distribution (p<5×10^−5^), a finding consistent with predictions of the HCV adapted model. This deviation resulted from two sequences (2C3 and 2C2) that contained a single shared polymorphismat position 2197 and two other sequences (2A2 and 2B34) that contained a different shared polymorphism at position 4344. The shared polymorphism at position 2197 was a first position GAG to TAG transversion that resulted in the introduction of a stop codon. Surprisingly, the same nonsense mutation was found in the identical position in two additional sequences (2B8 and 2B9) from this subject in a plasma sample taken 12 days earlier (Genbank accession nos. JQ803586, JQ803587, JQ803590 and JQ803591). We could be assured that these persistent but defective genomes were authentic and did not result from cross-contamination of amplicon sequences since each of the four sequences had additional distinguishing nucleotide polymorphisms (e.g., compare sequences 2C3 and 2C2 in Figure 4B) or were processed, PCR amplified and analyzed on different days (e.g., 2C3 and 2C2 versus 2B8 and 2B9). Occasional shared polymorphisms are commonly found in acute HIV-1 infection sequences and can be explained by polymerase errors early in infection being retained in the population [15], [49], but for neither HIV-1 nor HCV would nonsense mutations be expected to be retained unless they were complemented by competent genomes [50], [56], [57]. Regardless, the 60 sequences depicted in Figure 4B coalesced to a single unambiguous consensus, which we inferredto represent a likely T/F virus in this subject. To explore if additional T/F sequence lineages might have been overlooked due to inadequate sampling, we sampled 243 additional 5′ quarter genome sequences from this subject at three earlier time points spanning a 19 day period (Figures 1 and S2). Power calculations indicate that a sample size of 60 sequences provides 95% likelihood of detecting variants present at 5% in the population [15], whereas a sample size of 303 sequences provides 95% likelihood of detecting variants present at 1% prevalence. 43 of 46 (93%) of the sequences at the initial time point were identical, and diversity increased with time (Figure S2). Whether the 303 5′ quarter genome sequences from the different time points were considered separately or together, they conformed to a near star-like phylogeny and coalesced to the same T/F genome. Thus, we can conclude with a high level of confidence that subject 10051 was productively infected by a single virus whose sequence is represented by the consensus in Figure 4B and S2.

**Figure 5 ppat-1002880-g005:**
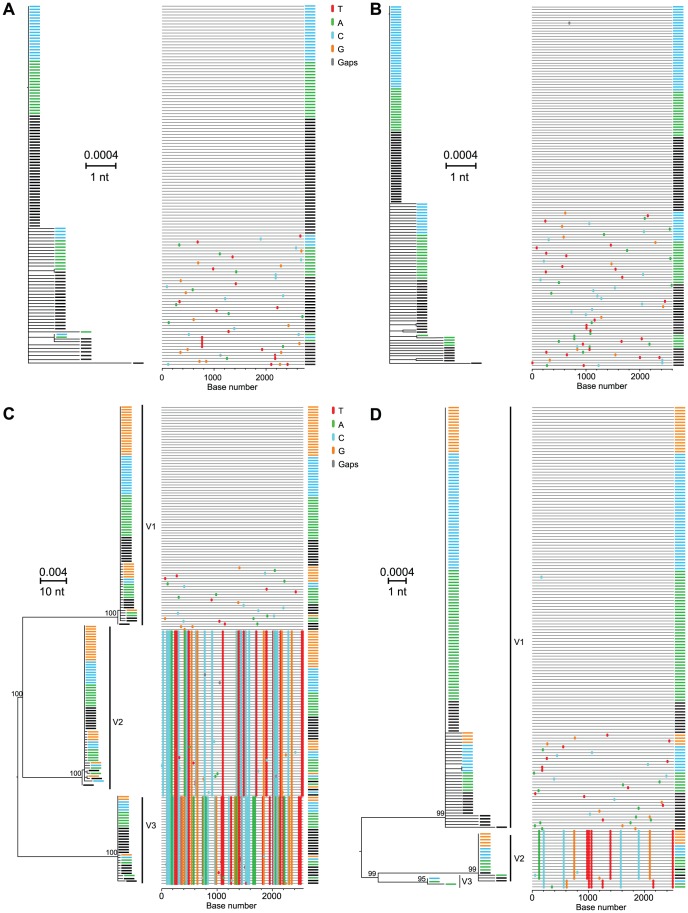
HCV diversity in acute infection. ML trees and *Highlighter* plots of 5′ quarter 2 genomesequences from four acutely infected subjects. Sequences are color coded to reflect sampling time points indicated in Figure 1. Subjects 10021 (A) and 10025 (B) revealed productive clinical infection by a single viruses whereas subjects 10012 (C) and 10062 (D) each showed infection by three viruses (v1–3).

**Figure 6 ppat-1002880-g006:**
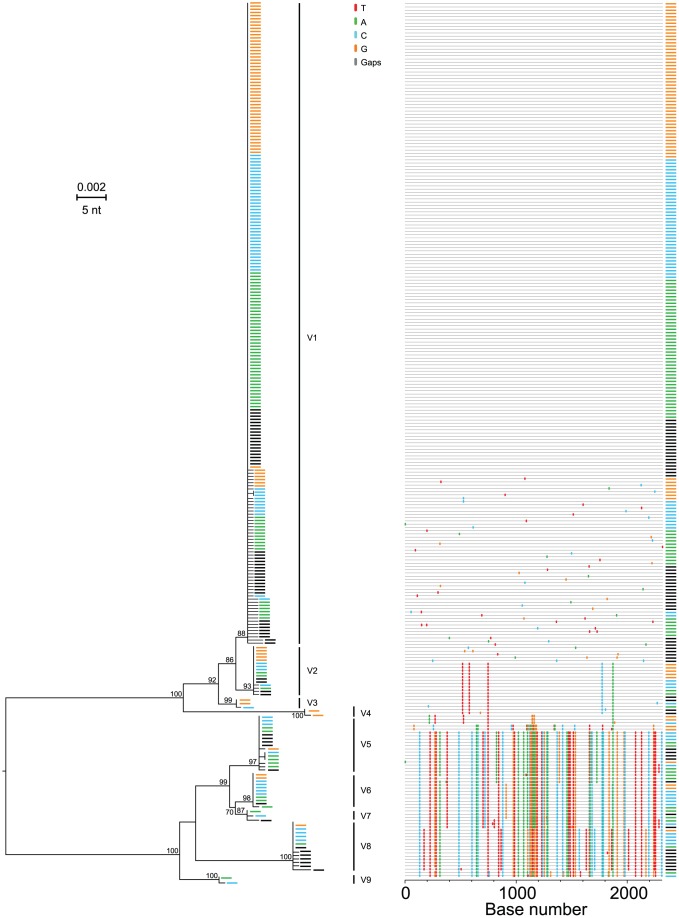
HCV diversity in acute subject 10029. MLtree and *Highlighter* plot of 5′ quarter 1 genomesequences,color coded to reflect sampling time points in Figure 1, reveal productive infection by 9genetically-distinct viruses.

Figure 5 extends the analysis of T/F HCV genomes to four acutely infected subjects where sequences from sequential time points revealed variable patterns of early viral diversity. In each subject, sequences from the initial sample were more homogeneous than those from later time points, as expected in a model of random accumulation of mutations. For subject 10021 (panel A), 19 of 24 (79%) sequences from the initial timepoint were identical. For subject 10025 (panel B), 32 of 43 (74%) of initial sequences were identical. The remaining sequences from this first time point in each subject differed from the respective consensus sequences by only 1 or 2 nucleotides. At the second and third sampling time points 1–4 weeks later, an increasing proportion of sequences from each subject differed from the respective consensus sequences by as many as 3 or 4 nucleotides. Interestingly, in both samples rare shared mutations became evident at later time points, again consistent with predictions of the HCV adapted model. For subjects 10021 and 10025, we thus concluded that the respective consensus sequences corresponded to single T/F HCV genomes. Of the 17 acutely infected subjects, four had evidence of productive clinical infection by single viruses (Tables 1 and S2). Panels C and D depict sequences from subjects 10012 and 10062, each of whom had evidence of productive infection by more than one genetically distinct virus based on the presence of multiple discrete low diversity lineages whose consensus sequences differed from each other by far more than the 2 nucleotides per quarter genome cut-off. Importantly, unlike HIV-1 where viral recombination in acute and early infection is extremely common [12], [16], we found no evidence of inter-lineage recombination in HCV sequences from these subjects or from any other subjects in this study.We thus interpreted the consensus sequences of each low diversity lineage in subjects 10012 and 10062 to correspond to a unique T/F HCV genome, three for each subject. This represents a minimum estimate, since deeper sampling could conceivably identify additional T/F sequence lineages, although with 188–230 sequences analyzed there was a 95% likelihood of detecting variants present at 2% in the population (Table S2). Figure 6 depicts sequences from subject 10029 where discrete low diversity sequence lineages indicated clinical acquisition of a minimum of 9 T/F viruses. In 6 additional acutely infected subjects, clearly distinguishable T/F lineages ranged from 3 to 13 per subject (Figures S3, S4, S5, S6, S7, S8).Importantly, for all of the acutely infected subjects described above, the numbers of T/F sequence lineages identified by visual inspection using the cut-off of >2 per quarter genome and >4 per half genome were nearly identical to those inferred from the HCV adapted model of early virus diversification using standard (maximum cut-off) or stringent (average cut-off) assumptions (Table S2).

### Evidence of acute-to-acute HCV transmission

**Figure 7 ppat-1002880-g007:**
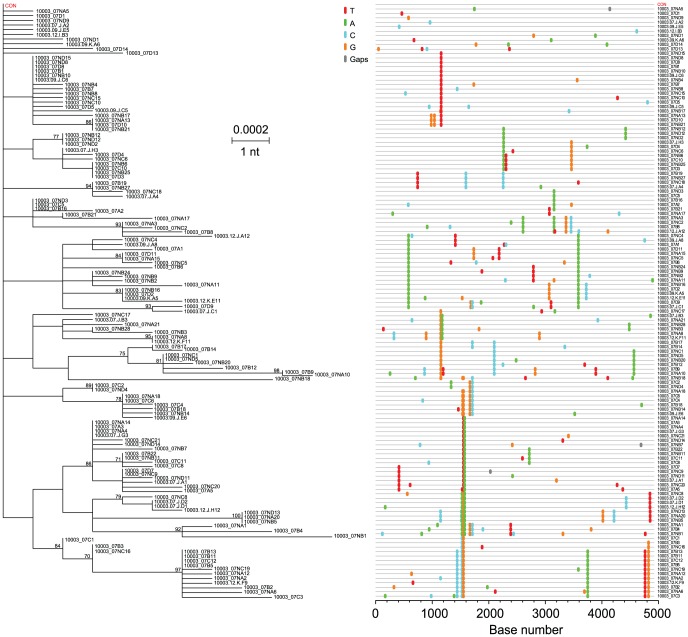
HCV diversity in subject 10003. ML tree and *Highlighter* plot of 5′ halfgenome sequences reveal many sets of closely related sequences distinguished by unique shared mutations.

Virus diversity in three acutely infected subjects (10003, Figure 7; 10020, Figure S9A; 10016, Figure S9B) shared features that distinguished them from the other 14 acute subjects. In each case, maximum sequence diversity was relatively low (0.27%–0.41%), consistent with recent infection. However, unlike in other subjects, samples from 10003, 10020 and 10016exhibited many closely related sets of sequences that shared unique polymorphisms, which appeared as multiple vertical ‘stripes’ in the *Highlighter* plots and shared nodes in the phylogenetic trees (Figures 7 and S9A,B). In subject 10003 (Figure 7), there were at least 37 distinctsequence sets, and in subjects 10020 (Figure S9A) and 10016 (Figure S9B) there were 10 and 15, respectively. Although we recognized that occasional shared mutations are predicted by the model and were found in samples from subjects infected by single or few viruses with well-defined T/F sequence lineages (e.g., see Figures 4B, 5 and 6), the high frequency of unique shared mutations in subjects 10003 (Figure 7), 10020 (Figure S9A) and 10016 (Figure S9B) was quite unusual and led us to hypothesize that most of the 10–37 distinct virus lineages observed in these individuals resulted from discrete transmitted viruses from individuals who themselves were recently infected by a single virus or by closely related viruses. An alternative hypothesis that we considered was that infection by single viruses had occurred in subjects 10003, 10020 and 10016but was followed by atypical diversification patterns not seen in any of the other acutely infected subjects. Our strategy to distinguish between these scenarios was both empirical and model-based. First, we observed that the maximum Hamming distances, expressed as per cent diversity, between the consensus sequences of the discrete sequence sets (i.e., between potential T/F genomes) from subjects 10016, 10020 and 10003, were 0.21%, 0.28% and 0.35% (mean 0.28%; median 0.28%), respectively. These values exceeded the maximum intra-lineage diversity (mean 0.12%; median 0.12%; range 0.04–0.19%; p = 0.0051, Mann-Whitney test) found in all lineages from all subjects in the initial ∼6 weeks of infection. These findings suggested acquisition of multiple distinct variants from recently infected subjects, not evolution of viruses from single transmitted variants. Secondly, we found that the multiple sequence sets in subjects 10016, 10020 and 10003 were present from the initial sampling time points and did not accumulate over time, again suggesting acquisition not evolution of the variants. Thirdly, we noted that the linear distribution of shared nonsynonymous nucleotide substitutions across the viral genomes in subjects 10016, 10020 and 10003 was not random but instead was concentrated in regions of E1 and E2 (Figure S10) previously associated with immune escape [41], [42], [58]. Inspection of the amino acid substitutions in the E2 region of the putative T/F genomes revealed combinations of different nonsynonymous mutations in identical or neighboring positions in the primary sequence that were unlikely to have occurred by chance (probability estimates <0.001). Since these subjects were sampled very early after infection, well before antibody seroconversion (Figure 1) or the onset of cellular immune responses [9], [10], the data suggest that the patterns of viral diversity observed in the putative T/F viral genomes from subjects 10016, 10020 and 10003 were the consequence of acquisition of multiple variants from transmitting individuals who themselves had become infected in the preceding ∼6 months and whose viral sequences had been subjected to early epitope-focused immune selection.

**Figure 8 ppat-1002880-g008:**
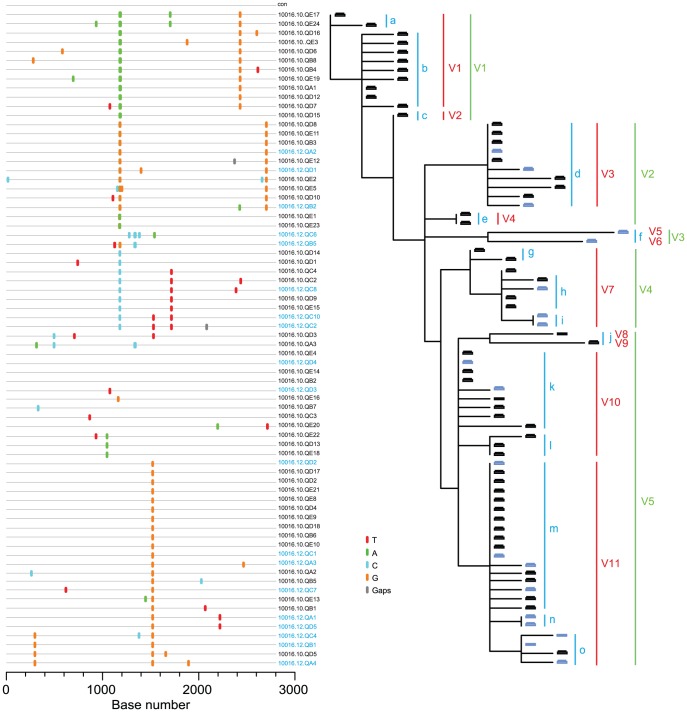
HCV diversity analysis in subject 10016 suggests acute-to-acute transmission. *Highlighter* plot and neighbor-joining tree of 5′ quarter 1 genome sequences. Visualization of 15 potential T/F viral lineages distinguished by unique shared mutations is indicated by lower case (blue) letters. Model estimates of T/F virus lineages using maximum (red) and average (green) cut-offs reveal 10 and 4 potential T/F virus lineages, respectively, based on increasingly stringent model assumptions (see text).

We next applied our HCV adapted model of early virus diversification to the sequences from the three subjects 10016, 10020 and 10003(Figures 8; S14–15). Under standard model assumptions (maximum cut-off, see methods) for delineating T/F lineages, transmission by at least 6–19 genetically distinct viruses was necessary to explain the viral diversity observed in the three subjects. Even under the most conservative model assumptions (average cut-off), transmission of at least 2–9 distinct viruses was required. Based on these findings, we conclude that the pattern of viral sequence variation in subjects 10003, 10020 and 10016can be most plausibly explained by acute-to-acute virus transmission.

### Transmission of multiple NS3A drug resistant variants

**Figure 9:**
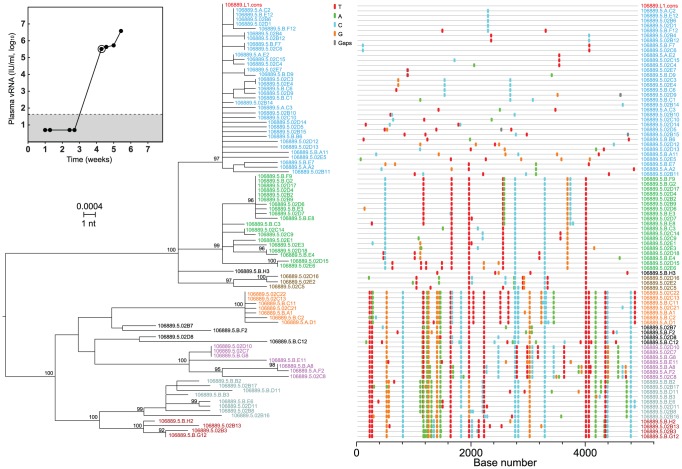
HCV plasma vRNA kinectics and diversity in subject 106889 reveals multivariant transmission of drug resistant mutants. vRNA kinetics (inset), ML phylogenetic tree, and *Highlighter* analysis of 5′ half genome sequences from the initial vRNA positive sample (circled). Sequences fall into distinct lineages (color-coded) with high statistical bootstrap support. All inferred T/F genomes contained V36M and R155K NS3 protease drug resistance mutations.

The phylogenetic pattern of sequences from subject 106889 (Figure 9) was still more complicated than that of any of the other 16 acutely infected subjects. This subject exhibited a typical acute infection viral kinetic profile with four sequential plasma samples negative for HCV vRNA and antibody followed by rapid vRNA ramp-up to nearly 4×10^6^ vRNA IU/ml (Figure 9 insert). These plasma samples from subject 106889 were obtained in June and July 2008 and were preceded by over 150 plasma collections from this individual in 2003–2008, all of which were negative for HCV RNA or antibody, proving this individual had incident infection. Eighty-seven 5′ half genome sequences were obtained from the initial plasma vRNA positive time point (Figure 9). Maximum diversity of these sequences was 1.03%, indicating multi-variant transmission. Similar to subjects 10003, 10020 and 10016, numerous discrete, low diversity sequence clades, many of which were closely related to each other, were apparent in the maximum-likelihood tree and *Highlighter* plot. Unlike sequences from the other subjects, however, the distinct sequence sets in 106889 clustered with high bootstrap values into larger lineages (color-coded in Figure 9). Thoseclades that contained sufficiently large numbers of sequences for analysis (e.g., clades identified by sequences 5.B.F9 and 5.02C22) exhibited a star-like phylogeny and Poisson distribution of mutations (Table 1), indicating that they had evolved very recently from discrete T/F genomes.These findings suggested that subject 106889 had been infected by large numbers of virusesfrom a chronically infected individual whose HCV sequences had been subjected to a stringent genetic bottleneck. A quite unexpected finding suggested a likely explanation: 86 of the 87 sequences from subject 106889 were found to contain two signature mutations in the NS3 protease gene (V36M and R155K) that confer high level drug resistance to the NS3 protease inhibitors Boceprevir and Telaprevir [7]. One of 87 sequences (sequence 02B11 in Figure 9) contained one of these DAA resistance mutations (V36M). Since the combination of V36M and R155K mutations is uncommon in treatment-naïve individuals [59], this result suggested that a transmitting partner to 106889 was chronically infected with HCV, was treated with an investigational NS3 protease inhibitor, experienced a DAA-induced viral population bottleneck followed by the emergence of NS3 protease resistant variants, and as these variants rebounded, transmitted multiple drug resistant variants directly to subject 106889 or indirectly through a second acutely infected individual. Under this scenario, where closely related sequences differing by as few as one nucleotide would be expected to be transmitted, we estimated by visual inspection of the phylogenetic tree that as many as 30 or more T/F virus could have been responsible for productive clinical infection (Table S2). By the standard model analysis, 28 distinct T/F viral genomes were necessary to account for the observed diversity. By the more conservative model analysis, at least 16 distinct T/F genomes were needed (Table S2).

### Virus diversification from T/F genomes

The identification of T/F viral sequencesin 17 subjects provided us with a unique opportunity for analyzing HCV sequence evolution in natural human infection beginning at or near the moment of virus transmission and extending through the establishment of early viral load setpoint, and in some subjects, antibody seroconversion. Among the 17 subjects, we identified a total of 146 T/F genomes. As expected, all 146T/F sequences had intact open reading frames for core, E1, E2, P7, NS2 and NS3. Thirty T/F genomes had 5 or more identifiable progeny (range 5–303; median 19) from which we could analyze molecular features of sequence diversification *in vivo* using phylogenetic tools and algorithms. A summary of this analysis is presented in Table 1. Maximum intra-lineage diversity for the 17 subjects ranged from 0.04% to 0.19% (mean = 0.12%; median = 0.12%), which was significantly lower than the maximum, mean and median viral diversities observed in chronically infected subjects (3.83%, 2.27% and 2.37%, respectively; p<0.0001, unpaired T-test with Welch's correction) (Tables 1 and S1; Figure S1). Evolved sequences compared to their respective T/F genomes revealed low frequencies of per nucleotide insertions (1×10^−6^), deletions (3×10^−6^) and stop codons (1×10^−6^). Transitions outnumbered transversions by 8.8 to 1, and when corrected for the number of available sites, by 18 to 1. The average dN/dS ratio was low at 0.39. The overall mutation frequencyamong all 17 acute subjects uncorrected for time from transmission or numbers of virus replication cycles was 1.4×10^−4^. When sequences were analyzed in the context of an agent-based stochastic model of virus diversification that incorporates estimated time from transmission and HCV specific parameters of virusreplication, the mutation rate of HCV *in vivo* was estimated to be 2.5×10^−5^ per nucleotide per genome replication [50]. We confirmed this low value by an analysis of the nonsense codon frequency per nonsense mutation target site [50]. Progeny of T/F viruses sampled at the earliest time points generally conformedto a star-like phylogeny and a Poisson distribution of randommutations (Table 1), but at later time points there were occasional deviations. Deviations from the model were of three types: (i) shared polymorphisms resulting from stochastic changes after the transmission event or from the transmission of multiple closely related viruses; (ii) immune selection or reversion in later samples at the time of antibody seroconversion in a single subject 9055 (Figures S16, S17); (iii) rare examples of short perfect inverted repeats, 3–20 nucleotides in length, that resulted from template switching between double-stranded RNA molecules in locations prone to RNA stem-loop secondary structure (Figure S18). This latter finding, which was found in 7 sequences out of 2922 analyzed,was observed in samples from three different study subjects. Four of these strand transfers occurred at the same location in the core gene.

## Discussion

The present study provides new quantitative and qualitative insights into HCV transmission and early diversification in humans. Previous reports documented a virus population bottleneck associated with HCV transmission, but none of those studies including ones based on 454 deep sequencing captured the broad range in multiplicity of infection or the full spectrum of genetic diversity that exists among transmitted viruses. In our study of 17 acutely infected subjects, we could unambiguously identify and determine the exact nucleotide sequences of one or more T/F virus genomes in each subject. This was true for all subjects whose HCV genomes were sequenced within the initial ∼6–8 weeks of infection; beyond that there were examples of immune selection that confounded the identification of T/F virus genomes (Figures S16 and S17). We estimated the multiplicity of infection (numbers of T/F viruses leading to productive clinical infection) to range from 1 to as many as 37 or more with a median of 4. These are minimum estimates given our sampling limitations, although we note that our median sampling depth of 151 sequences (Table 1) afforded us a 95% likelihood of detecting variants present at 2% prevalence [15]. In subjects productively infected by lower numbers of viruses (<10), where the progeny of each transmitted virus is repeatedly sampled, our estimates (Tables 1 and S1) are likely to be an accurate and precise measure of the number viruses that result in productive infection. In subjects infected by higher numbers of viruses, especially in the setting of acute-to-acute transmission where transmitted viruses are expected to differ by as few as one nucleotide, the accuracy of our estimates are necessarily less. This is because we could not sample deeply enough due to practical constraints of single genome sequencing of quarter and half genomes, and because we could not distinguish between transmitted viruses that differ by one or few nucleotides from single variant transmission followed by early stochastic mutations. However, based on the striking differences in diversity patterns that we observed between subjects with chronic-to-acute versus apparent acute-to-acute transmission, we suspect that the actual numbers of T/F viruses in subjects 10016, 10020, 10003 and 106889 approximate or exceed our estimates of 15, 10, 37 and 30 T/F genomes, respectively (Tables 1 and S2).

The broad range in numbers of T/F viruses responsible for acute HCV infection in our cohort must reflect the different transmission routes and risk practices ofsource plasma donors. Ostensibly, such individuals should be at low risk of acquiring HCV infection since they are qualified as regular source plasma donors only after extensive pre-enrollment screening that consists of medical histories, physical examinations and behavioral questionnaires designed specifically to eliminate from the donor pool individuals at risk for HCV, HBV or HIV infection (http://www.fda.gov/BiologicsBloodVaccines/GuidanceComplianceRegulatoryInformation/default.htm). However, self-reporting of risk behaviors among paid plasma donors is admittedly imperfect [60]. Thus, it is likely that the subjects in the present study represent the broad clinical spectrum of community-acquired HCV infection in the United States, which includes injection drug users, men who have sex with men, heterosexuals, and possibly, household contacts of HCV infected individuals.

Our findings regarding the multiplicity of human infection by HCV are quite different from those obtained by 454 pyrosequencing in seven acutely infected subjects reported by two different investigative groups where the range in T/F viruses was one to four with a median one [19], [43]. Our findings are also substantially different from estimates from other reports that employed reverse transcription, bulk PCR amplification, population sequencing or molecular cloning followed by sequencing [20], [23], [25], [30], [38], [41], [42].The latter studies showed that acutely infected subjects exhibited a spectrum in HCV sequence diversity that could at best be interpreted qualitatively as reflecting ‘few-variant’ versus ‘multi-variant’ transmission. We also note a recent study that used conventional bulk PCR amplification, cloning and sequencing to analyze acute and early HCV sequences consisting of a 225 bp hypervariable region of *env* from 10 acute infection subjects following IDU, sexual or nosocomial exposures [28]. Thisreportdescribed perplexing findings: 7 of 10 acutely infected subjects seemed to harbor more than one HCV genotype andsequential sequences obtained from these subjects a median of 17.5 days apart throughout the acute infection period suggested fluctuations in the prevalence of different HCV genotypes, subtypes and clades. These findings are at odds with our results (Figures 2, 4–9, S2, S3, S4, S5, S6, S7, S8, S9 and S14, S15, S16) and those of most other studies [24].

The SGA-direct amplicon sequencing strategy used in the present study represents a substantial advance in sensitivity and molecular resolution for distinguishing closely and distantly related T/F HCV genomes and their evolving progeny. Studies of HCV specific CTL recognition and escape [20], [25], [61], [62], neutralizing antibody recognition and escape [41], [42], and DAA drug resistance development [7] have previously been performed without a precise identification of T/F viral genomes and future studies may benefit from such an approach. We could readily distinguish evolving viral lineages that differed from the T/F genome by just 1 nucleotide in 5,000 (0.02%) at sites under selective pressure (Figures S16–17). This discriminating power further revealed evidence of acute-to-acute virus transmission in three subjects (Figures 7, 8 and S9) and DAA drug-induced viral genetic bottlenecking in a donor to a fourth acutely infected subject(Figure 9). This exquisite sensitivity in distinguishing T/F virus genomes and their progeny stands in contrast to the 454-based approaches, which were unable to distinguish between T/F viruses that differed by less than 2.5% [43] and bulk PCR-clone-sequencing methodsthat used a cutoff of 3% diversity to distinguish homogeneous from heterogeneous virus transmission [23]. Both of the latter methods are further confounded by the potential for *Taq* polymerase-mediated strand transfers leading to recombination artifacts in finished sequences [15], [19], [63].

The ability to identify actual T/F viral sequences and to track virus diversification from these sequences with single nucleotide resolution provided a unique opportunity to assess HCV sequence evolution *in vivo*. Virus diversification from discrete T/F viruses was generally star-like and conformed well to our HCV adapted model of early virus diversification. The finding of only a single instance of potential CTL escape or reversion among 17 acutely infected subjects at the last sampling time point is consistent with previous reports indicating substantial delays in the onset of adaptive immunity to HCV [9], [10]. The overall nucleotide substitution frequency that we observed among all subjects and including all sampling time points was 1.4×10^−4^. This substitution frequency is different from the mutation rate since it does not account for time, numbers of replication cycles, or different modes of HCV replication (linear versus geometric) [3], [57], [64], nor does it account for nucleotide substitutions introduced by the MuLV polymerase (Superscript III) during cDNA synthesis. The latter is estimated to occur at a frequency as low as 2×10^−6^ [65] and thus likely contributes negligibly to the mutation frequencies observed in the present study. Consistent with this interpretation were our results of single genome sequencing performed on the earliest vRNA positive plasma sample from subject 10051 where we found that 43 of 46 5′ quarter 1 genome sequences were identical (Figure S2). Among all 46 sequences, there were only four nucleotide substitutions in 100,050 nucleotides. This corresponds to a combined substitution frequency for the HCV polymerase and the Superscript III MuLV polymerase of 4×10^−5^, Again, thisresult does not account for the numbers of HCV replication cycles occurring between the moment of virus transmission and the time point of sampling, which in this case was very early during the viral ramp-upperiod when the plasma virus load was approximately 10,000 vRNA molecules/ml (Figure 1). In an accompanying report [50], we describe a new stochastic model of HCV replication and diversification that provides for a more precise estimation of the *in vivo* HCV RdRp error rate, which was found to be ∼2.5×10^−5^ per base per generation. This is lower than previous reports for HCV [56], [57] and comparable to the RT error rate of HIV-1 [57], [66].

We found a low dN/dS ratio consistent with early negative or purifying selection and a strong 18 to 1 mutational bias for transitions over transversions in acute infection. The latter finding is consistent with a recent report by Gotte and colleagues [67] who studied sequence evolution in chronically infected subjects and *in vitro* where a strong preference for G∶U/U∶G mismatches was observed for recombinant HCV RdRp. A mutational bias favoring transitions may be a factor besides RdRp error rate that influences the rate of development of DAA resistance mutations [67]. Importantly, we found no evidence of viral recombination in any subject, which would have been plainly evident in those subjects infected by multiple genetically diverse viral genomes (Figures 5C, D; 6; S3–9). The absence of recombination distinguishes HCV from HIV-1, where early recombination is widespread [12], [16], [46], but is consistent with molecular epidemiological data that suggest that HCV recombination is rare [68]–[71]. In addition, the failure to find plus-plus strand recombination in any of the sequences in the present report shows that strand switching by the MuLV reverse transcriptase (RT) *in vitro* must be extremely rare. This is important because it demonstrates that MuLV RT-mediated recombination does not confound single genome sequence analyses of HCV or other RNA viruses including HIV-1 andSIV [15], [18], [51]. On the other hand, we did observe seven examples of template switching between plus and minus strands of double-stranded HCV RNA templates (Figure S18). We could not determine if this resulted from strand switching by MuLV RT *in vitro* or by HCV RdRp *in vivo*. We note that Branch and colleagues [72] recently reported high levels of double-stranded HCV RNA in hepatic tissue, thus providing a plausible source of dsRNA for the observed template switching events.

A surprising finding of the current study was evidence of acute-to-acute HCV transmission in a relatively high proportion (3 of 17) of subjects. The acute infection period of HCV, like that of HIV-1, is characterized by very high plasma virus loads, absence of neutralizing antibodies, and rapid expansion of biologically fit virus populations that are homogeneous relative to the respective T/F virus genomes [22], [26], [27]. For HIV-1, the acute and early infection period has been shown to be associated with hyper-transmissibility with epidemiological studies and epidemic modeling indicating substantial enhancement in spread of the virus as long as six months post-transmission [73]–[76]. In the simian immunodeficiency virus (SIV) – Indian rhesus macaque transmission model, virus from acute infection plasma is up to 750-fold more transmissible on a per virion basis than is virus from chronic infection plasma [77]. To our knowledge, a clinical predilection for acute-to-acute HCV transmission has not previously been reported. In addition to the three subjects whom we identified with putative acute-to-acute HCV transmission, an argument can be made for an additional potential case in subject 10017 in whom distinct subsets of closely related T/F sequences were found within a context of high overall sequence diversity (e.g., see lineages v1 and v3; Figure S8). In this example, a plausible scenario is that a virus ‘donor’ to subject 10017 was acutely infected by multiple genetically-diverseviruses and that multiple progenyrepresenting several of these lineages were transmitted. The implication of these findings is that if the acute period of HCV infection is characterized by hyper-infectiousness as is the case for HIV-1, it could be a previously unrecognized but important contributing factor to the spread of HCV, potentially contributing to a recently described emerging HCV ‘epidemic’ in HIV-1 positive men who have sex with men [78], [79]. A limitation in our evidence supporting ‘acute-to-acute’ infection is that our study design did not allow us to identify paired donors and recipients of virus in order to analyze virus transmission directly. Future viral sequencing studies involving social networks of HCV transmission partners [80], or analyses of cryopreserved plasma specimens from previously conducted acute-to-acute human-to-chimpanzee HCV transmission studies [81], can provide corroborative evidence. We notethat there is precedent for phylogenetic linkage of HCV sequences in a human-to-human transmission case where clinical epidemiologic linkage between donor and recipient was established [42].

Still another surprising observation in this study was transmission of what we estimated to be as many as 30 NS3 protease-resistant viruses to subject 106889 (Figure 9). These mutations (V36M and R155K) confer high level resistance to both Boceprevir and Telaprevir, which were used in clinical trials near the time when 106889 samples were collected. Recently, we performed single genome sequencing of plasma viral RNA from subjects before and after treatment with a next generation investigational HCV protease inhibitor and observed viral genetic bottlenecking closely resembling that found in subject 106889 (unpublished data). To our knowledge, the data from subject 106889 is the first example of high multiplicity DAA drug resistant virus transmission, and the findings here illustrate how transmission of DAA resistant mutants can be deciphered with single genome specificity and sensitivity.

The identification of T/F genomes of HCV, HIV-1 [18], SIV [51] and potentially other RNA viruses by single genome sequencing is an enabling experimental strategy that captures molecular entities that are wholly sufficient and responsible for productive clinical infection and disease causation. In an accompanying report [50], we use sequences derived by this approach to analyze and mathematically model the early dynamics of HCV replication and diversification in acutely infected humans and derive new estimates of the *in vivo* mutation rate of HCV. A second application of the single genome sequencing method is to reveal through enumeration of T/F genomes, the challenge that vaccine candidates face in attempting to prevent or constrain HCV transmission. In a third application of the method, we previously demonstrated for HIV-1 that single genome sequencing allows for the molecular identification, cloning and biological characterization of full-length T/F genomes and a comprehensive proteome-wide analysis of autologous, strain-specific patterns of cytotoxic T-cell and neutralizing antibody responses [13], [18], [82]–[84]. By demonstrating that early HCV diversification generally conforms to a model of essentially random virus evolution where sequences coalesce to distinct, unambiguous T/F genomes, the present study has taken the first critical steps to demonstrate the feasibility of similar genome-wide analyses for HCV. An intriguing possibility is that full-length T/F HCV genomes, which by definition possess nucleotide and amino acid sequences sufficient for efficient *in vivo* replication in humans, can be identified, molecularly cloned and expressed for biological analyses in cell culture and animal models.

## Materials and Methods

### Ethics statement

This study was conducted according to the principles expressed in the Declaration of Helsinki. It was approved by the Institutional Review Boards of the University of Pennsylvania, the University of Alabama at Birmingham and Duke University. Subjects provided written informed consent for the collection of blood samples and subsequent analyses.

### Study subjects

Plasma samples were obtained from 17 subjects with acute HCV infection. These subjects were regular source plasma donors (ZeptoMetrix, Inc.; SeraCare, Inc.) who were HCV and HIV-1 antibody negative but who became HCV infected sometime in the course of their twice-weekly plasma donations as evidenced by the development of HCV viremia on sequential viral RNA testing (Figure 1). The subjects were asymptomatic throughout the collection period and did not receive anti-HCV treatment. By the time of the last sample collection, 5 of the subjects had seroconverted to HCV antibody positivity. Plasma samples from 14 patients from University of Alabama at Birmingham with chronic, treatment-naïve HCV infection were obtained as controls.

### HCV RNA and antibody assays

Plasma samples were tested for HCV RNA and antibodies by a battery of commercial tests. These included Roche COBAS AmpliPrep/COBAS Taqman HCV vRNA assay; ABBOTT Anti-HCV 3.0 Assay and ORTHO Enhanced SAVe Anti-HCV 3.0 Assay. HCV vRNA analyses were performed according to manufacturer's specifications (http://www.accessdata.fda.gov/cdrh_docs/pdf6/P060030c.pdf) in a CLIA certified laboratory with all assay controls meeting predetermined parameters for assay sensitivity and specificity and with a dynamic linear range of 43 to 6.9×10^7^ vRNA IU/ml.

### Viral RNA extraction and cDNA synthesis

For each plasma sample, approximately 100,000 viral RNA copies were extracted using the QiagenBioRobot EZ1 Workstation with EZ1 Vrius Mini Kit v2.0 (Qiagen, Valencia, CA). RNA was eluted and immediately subjected to cDNA synthesis. Reverse transcription of RNA to single stranded cDNA was performed using MuLV (SuperScript III) reverse transcriptase using methods recommended by the manufacturer (Invitrogen Life Technologies, Carlsbad, CA). Briefly, each cDNA reaction included 1× RT buffer, 0.5 mM of each deoxynucleoside triphosphate, 5 mM dithiothreitol, 2 units/ulRNaseOUT (RNase inhibitor), 10 units/ul of SuperScript III reverse transcriptase, and 0.25 uM antisense primer. The antisense primers were designed specifically for different genotype. 1.NS4A-R1 5′- GCACTCTTCCATCTCATCGAACTC -3′ (nt 5451–5474 H77 (accession number NC_004102)) for genotype 1, 2NS2-R1 5′-CCCCAGACGATGACTTTCTTCTCCAT-3′ (nt 5445–5467 H77) for genotype 2 and 3aNS3-R2V2 5′–TTACTTCCAGATCAGCTGACA-3′ for genotype 3. The reverse transcription reaction was carried out at 50°C for 60 minutes followed by an increase in temperature to 55°C for an additional 60 minutes. The reaction was then heat-inactivated at 70°C for 15 minutes and then treated with 0.1 U/ul RNaseH at 37°C for 20 minutes. The newly synthesized cDNA was used immediately or kept frozen at −80°C.

### Single genome amplification

cDNA was serially diluted and distributed among wells of replicate 96-well plates (Applied Biosystems, Foster City, CA) so as to identify a dilution where PCR positive wells constituted less than 30% of the total number of reactions. At this dilution, most wells contain amplicons derived from a single cDNA molecule. This was confirmed in every positive well by direct sequencing of the amplicon and inspection of the sequence for mixed bases (double peaks), which would be evidence of priming from more than one original template or the introduction of PCR error in early cycles. Any sequence with evidence of mixed bases was excluded from further analysis. PCR amplification was carried out in the presence of 1× High Fidelity Platinum PCR buffer, 2 mM MgSO_4_, 0.2 mM of each deoxynucleoside triphosphate, 0.2 uM of each primer, and 0.025 units/ul Platinum Taq High Fidelity polymerase in a 20 ul reaction (Invitrogen, Carlsbad, CA). The nested or hemi-nested primers for generating 5′ half or 5′ quarter genome from different genotypes included: (1) 5′ half genome of genotype 1: 1^st^ round sense primer 1.core.F1 5′-ATGAGCACGAATCCTAAACCTCAAAGA-3′ (nt 342–368 H77) and 1^st^ round antisense primer 1.NS4A.R1 5′-GCACTCTTCCATCTCATCGAACTC-3′ (nt 5451–5474 H77), 2^nd^ round sense primer 1.core.F2 5′- TCAAAGAAAAACCAAACGTAACACCAACCG-3′ (nt 362–391 H77 and 2^nd^ round antisense primer 1.NS3A4A.R2 5′- AGGTGCTCGTGACGACCTCCAGG-3′ (nt 5297–5319 H77); (2) 5′ quarter genome of genotype 2: 1^st^ round sense primer 2.core.F1 5′- ATGAGCACAAATCCTAAACCTCAAAGA-3′ (nt 342–368 H77) and 1^st^ round antisense primer 2.NS2.R1 5′- CCCCACACAATGACCTTCTTCTCCATTG -3′ (nt 5445–5467 H77), 2^nd^ round sense primer 2.core.F2 5′- AATCCTAAACCTCAAAGAAAAACCAAA -3′ (nt 351–377 H77) and 2^nd^ round antisense primer 2.NS2.R2 5′- GGGGAGAGGTGGTCATAGATGTAA -3′; (3) 5′ half genome of genotype 3: 1^st^ round sense primer 3a.core.F1 5′- ATGAGCACACTTCCTAAACCTCAAAGA -3′ and 1^st^ round antisense primer 3aNS3-R1V2 5′-TTACTTCCAGATCAGCTGACA-3′, 2^nd^ round sense primer 3a.core.F2 5′- TCAAAGAAAAACCAAAAGAAACACCATCCG -3′ and 2^nd^ round antisense primer PCR 3a.NS3-R2V2 5′-TTACTTCCAGATCAGCTGACA -3′. PCR was performed in MicroAmp 96-well reaction plates (Applied Biosystems, Foster City, CA) with the following PCR parameters: 1 cycle of 94°C for 2 min; 35 cycles of a denaturing step of 94°C for 15 s, an annealing step of 58°C for 30 s, an extension step of 68°C for 5 min, followed by a final extension of 68°C for 10 min. The product of the 1^st^ round PCR was subsequently used as a template in the 2^nd^ round PCR under same conditions but with a total of 45 cycles. Amplicons were inspected on precasted 1% agarose E-gels 96 (Invitrogen Life Technologies, Carlsbad, CA). All PCR procedures were carried out under PCR clean room conditions using procedural safeguards against sample contamination, including pre-aliquoting of all reagents, use of dedicated equipment, and physical separation of sample processing from pre- and post-PCR amplification steps.

### DNA sequencing

PCR amplicons were directly sequenced by cycle-sequencing using BigDye terminator chemistry and protocols recommended by the manufacturer (Applied Biosystems; Foster City, CA). Sequencing reaction products were analyzed with an ABI 3730xl genetic analyzer (Applied Biosystems; Foster City, CA). Both DNA strands were sequenced using partially overlapping fragments. Individual sequence fragments for each amplicon were assembled and edited using the Sequencher program 5.0 (Gene Codes; Ann Arbor, MI). Inspection of individual chromatograms allowed for the identification of amplicons derived from single versus multiple templates. The absence of mixed bases at each nucleotide position throughout the entire 5′ half or quarter genome sequences was taken as evidence of amplification from a single viral RNA/cDNA template. This quality control measure enabled us to exclude from the analysis amplicons that resulted from PCR-generated *in vitro* recombination events or *Taq* polymerase errors and to obtain multiple individual sequences that proportionately represented those circulating HCV virions.

### Sequence alignments

All the sequences alignments were initially made with ClustalW and then hand-checked using MacClade 4.08 to improve the alignments according to the codon translation. All 2922 5′ half or quarter-genome sequences from acute and chronic patients were deposited in GenBank and edited alignments can be accessed at http://www.hiv.lanl.gov/content/sequence/hiv/user_alignments/Li2012.html.

### Sequence diversity analysis

Two thousand three 5′ half genomes and 919 5′ quarter genomes were amplified by SGA-direct amplicon sequencing from the 31 subjects. Half genome sequences were generated so as to obtain longer sequences for linkage analyses, whereas quarter genome sequences were generated to enhance sensitivity of amplification from early samples with lower viral loads. Among the 3168 amplicons generated, the sequences of 2922 were unambiguous at every position. The other 246 amplicons contained one or more “double peaks” representing mixed bases and these were discarded and not included in the analysis. The median number of sequences analyzed per time point was 54 (mean = 54; range = 5–122) for acutely infected subjects and 25 (mean = 27; range = 13–44) for chronically infected subjects. A total of 2922 sequences from all 17 acutely-infected and 14 chronically-infected subjects were analyzed using phylogenetic tree analysis together with a sequence visualization tool, *Highlighter* (www.HIV.lanl.gov), that allows tracing of common ancestry between sequences based on individual nucleotide polymorphisms. Phylogenetic trees were generated by maximum likelihood methods using PhyML [85] or RAxML-VI-HPC [86]. For subjects productively infected by more than one T/F virus, lineages contained more than 5 closely related sequences were included in the lineage diversity analyses. Lineage-specific sequences were analyzed by the Poisson Fitter program (www.HIV.lanl.gov).

### Mutation rate calculation

A total of 30 T/F lineages from 17 acutely-infected subjects were analyzed. Each sequence within the lineage was compared with the T/F virus sequence of that lineage. The insertion, deletion, transition and transversion frequencies were counted manually or by computer program. The rates were calculated by taking the ratio of each frequency number and the total number of nucleotides of all the sequences within that lineage.

### Synonymous and non-synonymous substitution rate analysis

The SNAP program (www.HIV.lanl.gov) was applied to the codon-aligned sequences of each T/F lineage. Within each lineage, the number of synonymous and nonsynonymous substitutions were derived by comparing to the T/F viral sequences. The accumulation rates of synonymous substitutions per potential synonymous site and nonsynonymous substitution per potential nonsynonymous site were compared to screen for positive selection.

### Mathematical models and algorithms for estimating numbers of T/F variants

Two mathematical models were used to analyze early HCV sequence diversity. The first was originally designed for HIV-1 and has been previously described in detail [15], [48], [49]. The second is a simplified deterministic model that accounts for the essential differences in replication dynamics between HCV and HIV-1, taking into account HCV's life cycle, that HCV replication occurs via a cytosolic replication complex, and that there can be many replication complexes continuously producing viruses from a long-lived infected cell. In this model, each HCV replication complex was assumed to give rise to a new replication complex at regular intervals by undergoing two RdRp copying events. These complexes, which may reach 40 per cell [53], were presumed to persist and produce viruses for the entire duration of the acute infection sampling period. Because of the sequential creation of the complexes, those at the same generation depth have widely varying number of descendants, unlike the situation in HIV (Figure 3). An average pair of HCV viruses then has a later most common ancestor than does HIV. As a result, the model predicts that sequences with a small number of shared mutations can arise in a subject at detectable frequencies prior to the onset of immune selection. This translates to an expectation of about 3 times larger numbers of stochastically shared mutations in HCV than in HIV, with the potential to violate the star-like hypothesis more often. Furthermore, the persistence of HCV replication complexes of all generations means that, in contrast to the situation in HIV, the Poisson distribution is not necessarily a good model for inter-sequence distances prior to selection, and sequences with distances that deviate from Poisson distribution canbe derived from a single T/F viral genome. Our simple model of HCV diversification accounts for these issues and is amenable to an analytical approach. At the same time, the numerical results from the simplified model are consistent with those from a more detailed agent based stochastic model of early HCV infection that we present in an accompanying paper [50].

The implementation of the clustering algorithm works on a phylogenetic tree describing the evolutionary relations between these sequences and aims to identify monophyletic clusters that could reasonably have arisen by evolution in the infected individual. The modeling in Ribeiro *et al.* [50] shows that these clusters should satisfy two separate criteria: (a) the total number of mutations that could have accumulated is limited by the mutation rate of the virus and the generation time, and (b) the number of mutations shared by distinct sequences from a single T/F virus is related by coalescent theory to the growth and stabilization of viral load in these acute infections. Starting at the tips of the phylogenetic tree, our algorithm identifies the largest clusters that are consistent with these two criteria. Following from (a), every replication complex present in the body produces a new generation of replication complexes about once a day. Following from this, at time *t* days into infection, the most divergent replication complex is *t* generations from the founder, but most replication complexes are of generation *t/2*. We therefore split the clusters whose average divergence is larger than expected from this scenario. Following from (b), if one samples approximately 30–100 sequences in the initial weeks of infection prior to the onset of immune selection, 1–10 pairs of sequences are expected to coalesce 4–7 generations after the T/F virus.Assuming a mutation rate of 2.5×10^−5^ per base per generation [50], this corresponds to a probability of more than 5% of seeing 3–4 shared mutations in 5000 bases. Thus, to be conservative in our model estimates of minimum numbers of T/F genomes, wedefined clusters of sequences as having >4 shared mutations in a half genome, or >2 in a quarter genome, as unambiguously to have arisen from a different founder variant.

In the case of acute-to-acute infections, however, the average divergence is a very weak measure for delineating the clusters. In particular, transmission of one or a few highly divergent sequences affects the average little, and they are often not identified as separate variants when they are likely to represent a distinct founder. Thus, mean diversity is a robust measure but occasionally allows one or a few highly divergent sequences to be counted as a part of the cluster. To provide an estimate of the number of T/F variants that takes this into account, we also calculated the number of clusters by implementing a threshold on the most distant tip from the inferred ancestor for each cluster, splitting clusters based on the maximum distance that is improbable. Though this provides a better overall estimate for the number of clusters, such extreme-value statistics are more affected by the approximations made in going from the fully stochastic to the deterministic model. We verified that the main conclusions of this paper, including the identification of three subjects as cases of acute-to-acute transmission, follow from either of the two methods of identifying T/F lineages. Both of these methods provide a minimum estimate for the number of T/F viruses for two reasons. First, the number of shared mutations we have allowed within a cluster provides a highly conservative criterion and lineages could still contain closely related but distinct transmitted viral sequences, a situation made likely by our identification of probable cases of acute-to-acute virus transmission. Second, finite depth of sequencing means we might miss T/F clusters that are represented by a small fraction of sequences. To estimate the impact of this second scenario, we performed direct power calculations as previously described [15] to assess the likelihood of missing infrequent T/F sequence lineages based on sampling depth.

Finally, acute samples are often very homogeneous and can have conflicting phylogenetic signals, and these calculations rely on the phylogenetic tree being a description of the true evolutionary relations. In cases of infection with two highly divergent strains, the descendants of the two strains form distant outgroups from each other, and homoplasy on the long branch linking the two may root the individual clusters suboptimally. To avoid this problem, our algorithm looks for long branches (longer than 30 mutations), deletes them, and applies the rest of the algorithm to each of the then disconnected clusters.After identifying these clusters, the roots of each of these is chosen as tentative transmitted founder viruses. The paths in the phylogenetic tree between these roots represent evolution that happened prior to infection, whereas the paths linking the roots to the tips indicate within host evolution. Occasionally, we find overlap between the two sets of paths indicating convergent evolution between the donor and the recipient infected individuals. Since convergent evolution is unlikely, we further subdivide the previously found clusters to avoid this.After finding the optimal clustering at each time point, the results are mapped on to a tree of all the sequences obtained at various time points from the same individual. The overall number of clusters is then found by merging clusters from different time points that interleave, and leaving the other clusters as distinct. In the scenario of early infection when there are very few mutations, the tree can be ambiguous because of single conflicting mutations causing polytomies; while this could theoretically make the number of transmitted variants ambiguous, this was not the case in our analysis of this sequence set.Codes written in C that implement these two clustering strategies (using either maximum or average distances to define clusters) are available at http://www.santafe.edu/~tanmoy/programs/HCV/.

### Statistical analyses and power calculations

Standard descriptive statistics including Mann-Whitney and unpaired T-tests with Welch's correction were employed and identified throughout the text. Power calculations to estimate the likelihood of detecting rare sequence variants based on sampling depth were performed as previously described [15]. Estimates of the probability that observed clusters of nonsynonymous mutations in the Env E2 coding region of putative T/F viral genomes from subjects 10003, 10016 and 10020 could have occurred by chance were performed with a binomial expansion as previously reported [87]. We considered ten codon windows because this could generally span the observed clusters as well as typical T-cell epitopes and potential linear neutralizing antibody epitopes. From the binomial expansion, the probability of seeing at least the observed number (*k*) of clustered mutations within a single 10-mer is:
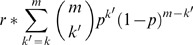
where *r* is the number of potential 10-mers, *m* is the total number of mutations relative to the consensus, and *p* is the probability that a mutation falls by chance in any particular 10-mer. We calculated the probability only for *k*′ = *k*, since the results for all *k*′>*k* are very much smaller and can be ignored. Only nonsynonymous mutations were included in the analysis. Within the 10-mers of interest, but not elsewhere along the alignment, different amino acid substitutions and combinations of substitutions were identified and analyzed.

## Supporting Information

Figure S1 **Maximum diversity of discrete HCV sequence lineages from acute infection subjects versus maximum sequence diversity in chronic subjects.** Primary data are derived from Tables 1 and S1. Mean (±95% CI) values are represented by horizontal lines. Differences between the two groups were highly significant (p<0.0001; unpaired T-test with Welch's correction), reflecting the recent and remote diversification histories of acute and chronic sequences, respectively.
(PDF)

Figure S2 **HCV diversity in acute subject 10051.** 5′ quarter 1 genomesequences are color coded in orange, green, blue and black in chronological order to reflect sampling time points in Figure 1 and are represented in a ML tree and *Highlighter* plot. Sequences show evidence of productive clinical infection by a single virus. The horizontal scale bar indicates genetic distance.
(PDF)

Figure S3 **HCV diversity in acute subject 10024.** 5′ quarter 1 genomesequences are color coded in blue, green and black in chronological order to reflect sampling time points in Figure 1 and are represented in a ML tree and *Highlighter* plot. Sequences show evidence of productive clinical infection by at least 6 T/F viruses. Bootstrap values are indicated and represent 100 repetitions. The horizontal scale bar indicates genetic distance.
(PDF)

Figure S4 **HCV diversity in acute subject 6213.** 5′ half genomesequences are represented in a ML tree and *Highlighter* plot. Sequences show evidence of productive clinical infection by at least 3 T/F viruses. Bootstrap values are indicated and represent 100 repetitions. The horizontal scale bar indicates genetic distance.
(PDF)

Figure S5 **HCV diversity in acute subject 6222.** 5′ half genomesequences are represented in a ML tree and *Highlighter* plot. Sequences show evidence of productive clinical infection by at least 4 T/F viruses. Bootstrap values are indicated and represent 100 repetitions. The horizontal scale bar indicates genetic distance.
(PDF)

Figure S6 **HCV diversity in acute subject 10004.** 5′ quarter1 genomesequences are represented in a ML tree and *Highlighter* plot. Sequences show evidence of productive clinical infection by at least 3 T/F viruses. Bootstrap values are indicated and represent 100 repetitions. The horizontal scale bar indicates genetic distance.
(PDF)

Figure S7 **HCV diversity in acute subject 10002.** 5′ half genomesequences are represented in a ML tree and *Highlighter* plot. Sequences show evidence of productive clinical infection by at least 13 T/F viruses. Bootstrap values are indicated and represent 100 repetitions. The horizontal scale bar indicates genetic distance.
(PDF)

Figure S8 **HCV diversity in acute subject 10017.** 5′ quarter 1 genomesequences are color coded in red, orange, green, blue and black in chronological order to reflect sampling time points in Figure 1 and are represented in a ML tree and *Highlighter* plot. Sequences show evidence of productive clinical infection by at least 4 T/F viruses. Bootstrap values are indicated and represent 100 repetitions. The horizontal scale bar indicates genetic distance.
(PDF)

Figure S9 **HCV diversity in acute subjects 10020 and 10016.** 5′ quarter1 genome sequences from subject 10020 (panel A) and 10016 (panel B) are depicted by ML tree and *Highlighter* plots. Many sets of closely related sequences distinguished by unique shared mutations are evident. Bootstrap values represent 100 repetitions.
(PDF)

Figure S10 **Nonsynonymous and synonymous mutations in HCV sequences from acute subjects 10003, 10020 and 10016.** *Highlighter* plotsof 5′ half or quarter1 genomesequences are color coded to denote nonynonymous (red) and synonymous (green) mutations for subjects 10003 (panel A), 10020 (panel B) and 10016 (panel C).
(PDF)

Figure S11 **Amino acid alignment of the HCV Env coding region of acute subject 10003.** The H77 reference sequence is shown at the top. Nonrandom concentrations of nonsynonymous mutations are evident.
(PDF)

Figure S12 **Amino acid alignment of the HCV Env coding region of acute subject 10020.** The H77 reference sequence is shown at the top. Nonrandom concentrations of nonsynonymous mutations are evident.
(PDF)

Figure S13 **Amino acid alignment of the HCV Env coding region of acute subject 10016.** The H77 sequence is shown at the top. Nonrandom concentrations of nonsynonymous mutations are evident.
(PDF)

Figure S14 **HCV diversity analysis in subject 10020 suggests acute-to-acute transmission.** *Highlighter* plot and neighbor-joining tree of 5′ quarter 1 genome sequences. Visualization of 10 potential T/F viral sequences distinguished by unique shared mutations is indicated by lower case (blue) letters. Model estimates of T/F virus lineages using maximum (red) and average (green) cut-offs reveals 5 and 3 potential T/F virus lineages, respectively, based on increasingly stringent model assumptions (see text).
(PDF)

Figure S15 **HCV diversity analysis in subject 10003 suggests acute-to-acute transmission.** *Highlighter* plot and neighbor-joining tree of 5′ half genome sequences. Visualization of 37 potential T/F viral sequences distinguished by unique shared mutations is indicated by lower case (blue) letters. Model estimates of T/F virus lineages using maximum (red) and average (green) cut-offs reveals 15 and 8 potential T/F virus lineages, respectively, based on increasingly stringent model assumptions (see text).
(PDF)

Figure S16 **HCV diversity in acute subject 9055.** 5′ half genomesequences are color coded in green, blue and black in chronological order to reflect sampling time points in Figure 1 and are represented in a ML tree and *Highlighter* plot. Sequences show evidence of productive clinical infection by one T/F virus with mutations accumulating at positions 3311 and 3346 at the last sampling time point coincident with antibody seroconversion. Bootstrap values represent 100 repetitions. The horizontal scale bar indicates genetic distance.
(PDF)

Figure S17 **Nonsynonymous and synonymous mutations in HCV sequences from acute subject 9055.** A *Highlighter* plot (panel A) of 5′ half genomesequences is color coded to denote nonynonymous (red) and synonymous (green) mutations. The boxed area reveals a temporal expansion of sequences with concentrated amino acid subtitutions in NS3. In panel B, amino acid selection is evident in a previously identified CTL epitope highlighted in red. The top-most sequence represents the genotype 3a consensus.
(PDF)

Figure S18 **Polymerase template switching.** Template switching by the RNA polymerase between minus sense and plus sense strands of double-stranded HCV RNA resulted in short stretches of perfect inverted repeat sequences. Five examples are illustrated in panels A–E. Proposed mechanisms for template switching are illustrated.
(PDF)

Table S1 **Diversity analysis of HCV 5′ half genome sequences from 14 chronically infected subjects.**
(DOC)

Table S2 **Estimates of numbers of T/F viruses in acute HCV infection using empirical and model based methods.**
(DOC)

Table S3 **Estimation of numbers of T/F viruses by time point using model-based methods with different cut-offs.**
(DOC)
