# Supplementary figures and images for: Elucidation of Hepatitis C Virus Transmission and Early Diversification by Single Genome Sequencing
Source: PLoS Pathog. 2012 Aug 23;8(8):e1002880. doi: 10.1371/journal.ppat.1002880 (PMC3426529; doi:10.1371/journal.ppat.1002880)

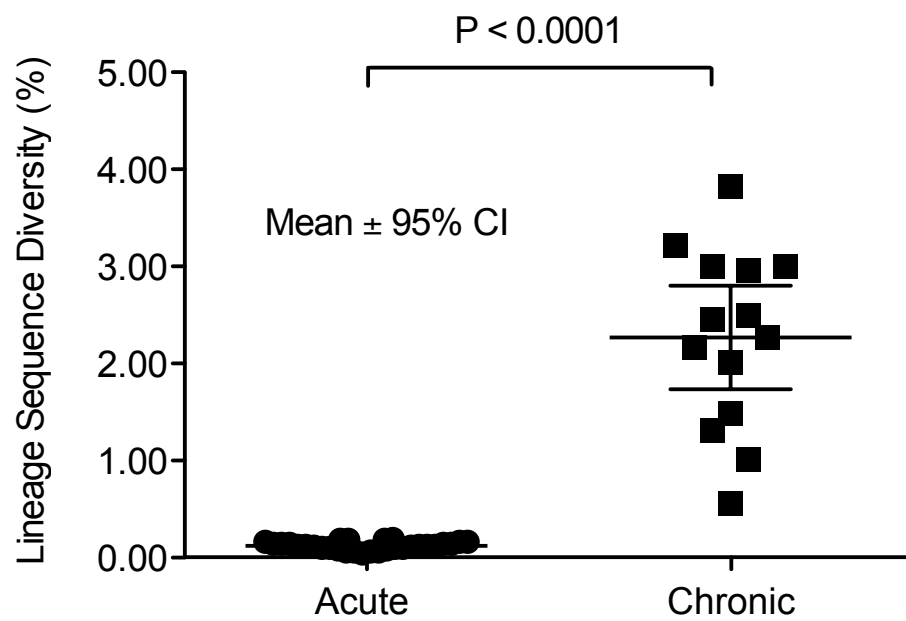

Figure S1

Supplement: Figure S1 — Maximum diversity of discrete HCV sequence lineages from acute infection subjects versus maximum sequence diversity in chronic subjects. Primary data are derived from Tables 1 and S1. Mean (±95% CI) values are represented by horizontal lines. Differences between the two groups were highly significant (p<0.0001; unpaired T-test with Welch's correction), reflecting the recent and remote diversification histories of acute and chronic sequences, respectively. (PDF) [file ppat.1002880.s001.pdf]

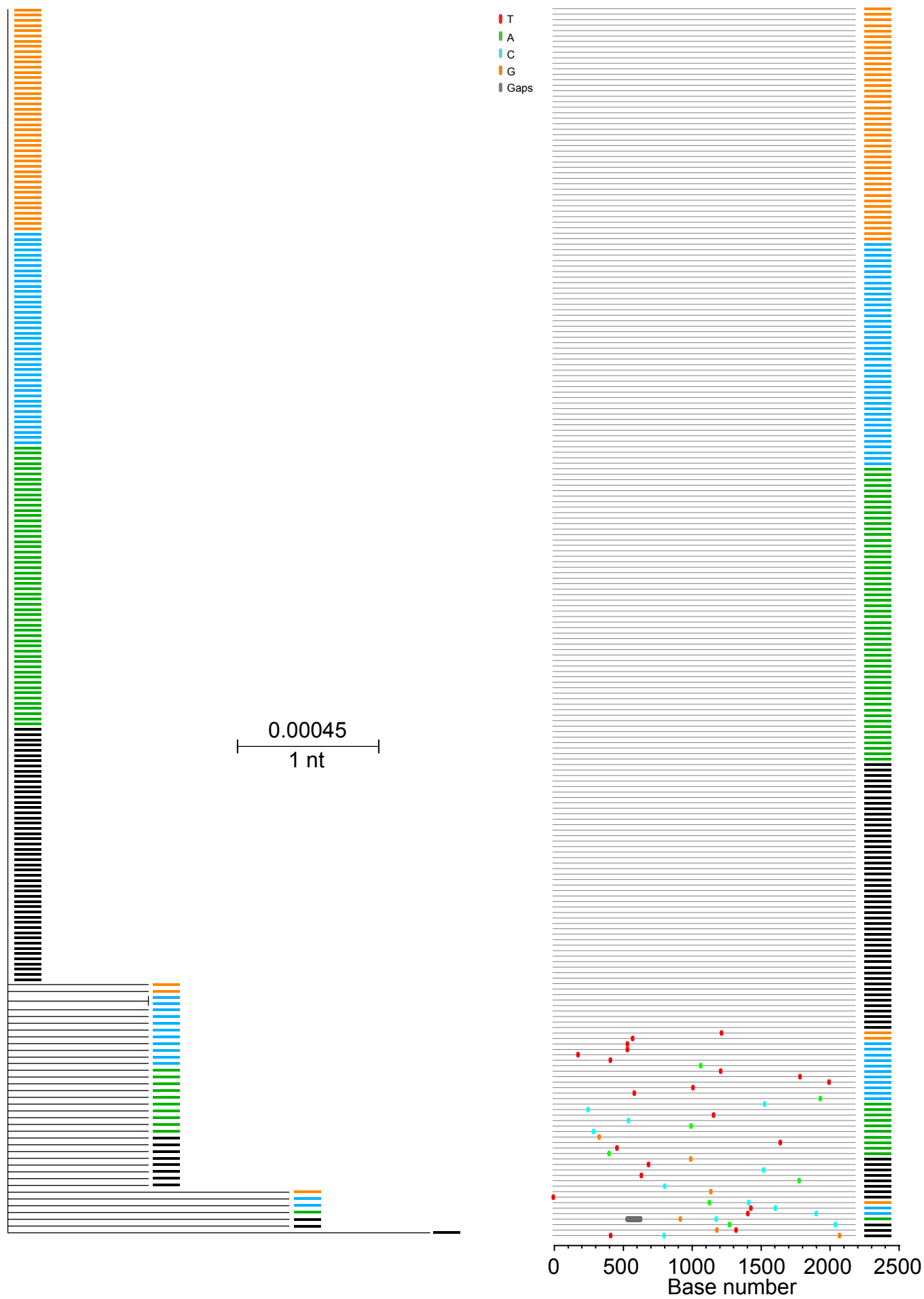

Figure S2

Supplement: Figure S2 — HCV diversity in acute subject 10051. 5′ quarter 1 genomesequences are color coded in orange, green, blue and black in chronological order to reflect sampling time points in Figure 1 and are represented in a ML tree and Highlighter plot. Sequences show evidence of productive clinical infection by a single virus. The horizontal scale bar indicates genetic distance. (PDF) [file ppat.1002880.s002.pdf]

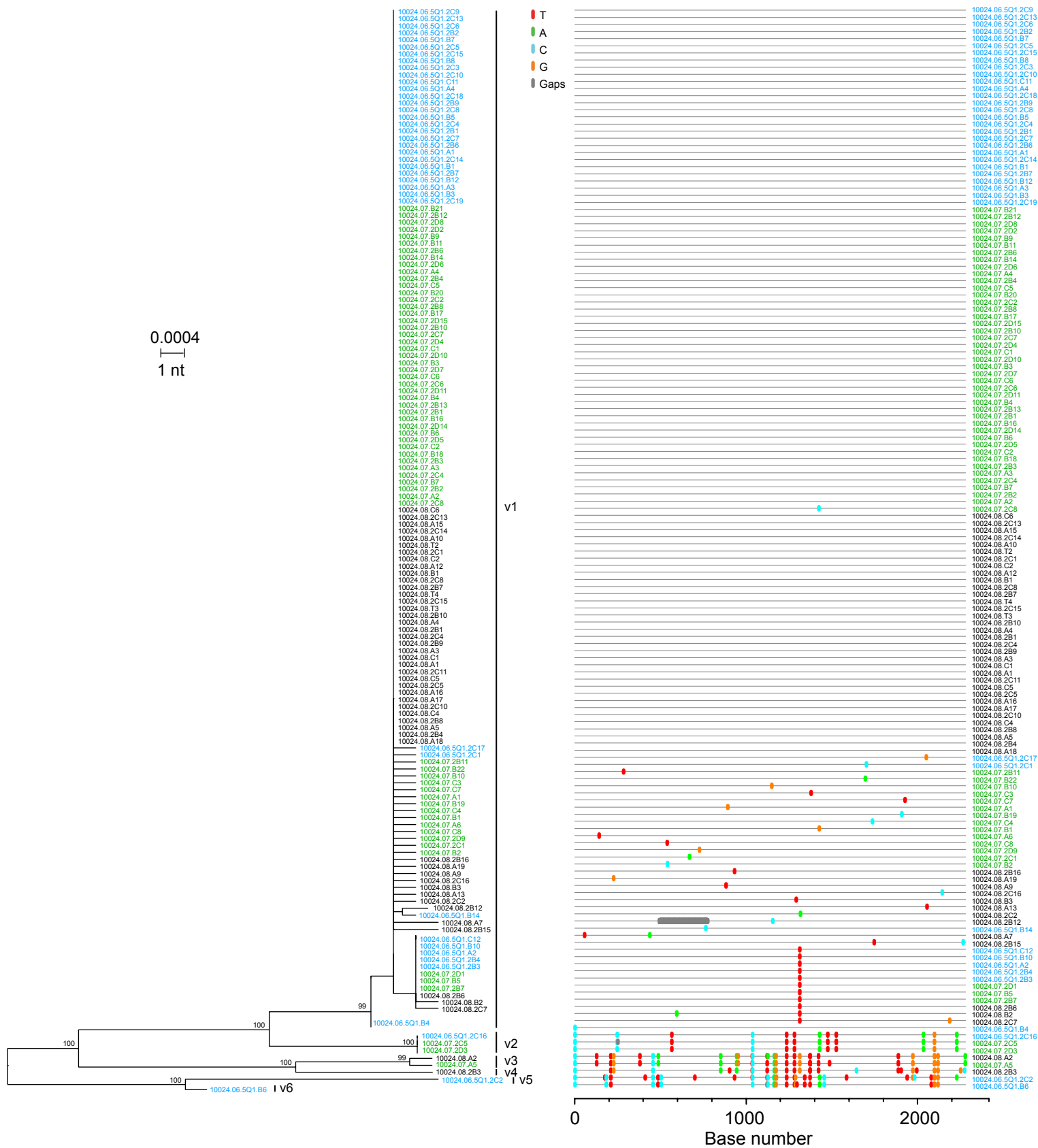

Figure S3

Supplement: Figure S3 — HCV diversity in acute subject 10024. 5′ quarter 1 genomesequences are color coded in blue, green and black in chronological order to reflect sampling time points in Figure 1 and are represented in a ML tree and Highlighter plot. Sequences show evidence of productive clinical infection by at least 6 T/F viruses. Bootstrap values are indicated and represent 100 repetitions. The horizontal scale bar indicates genetic distance. (PDF) [file ppat.1002880.s003.pdf]

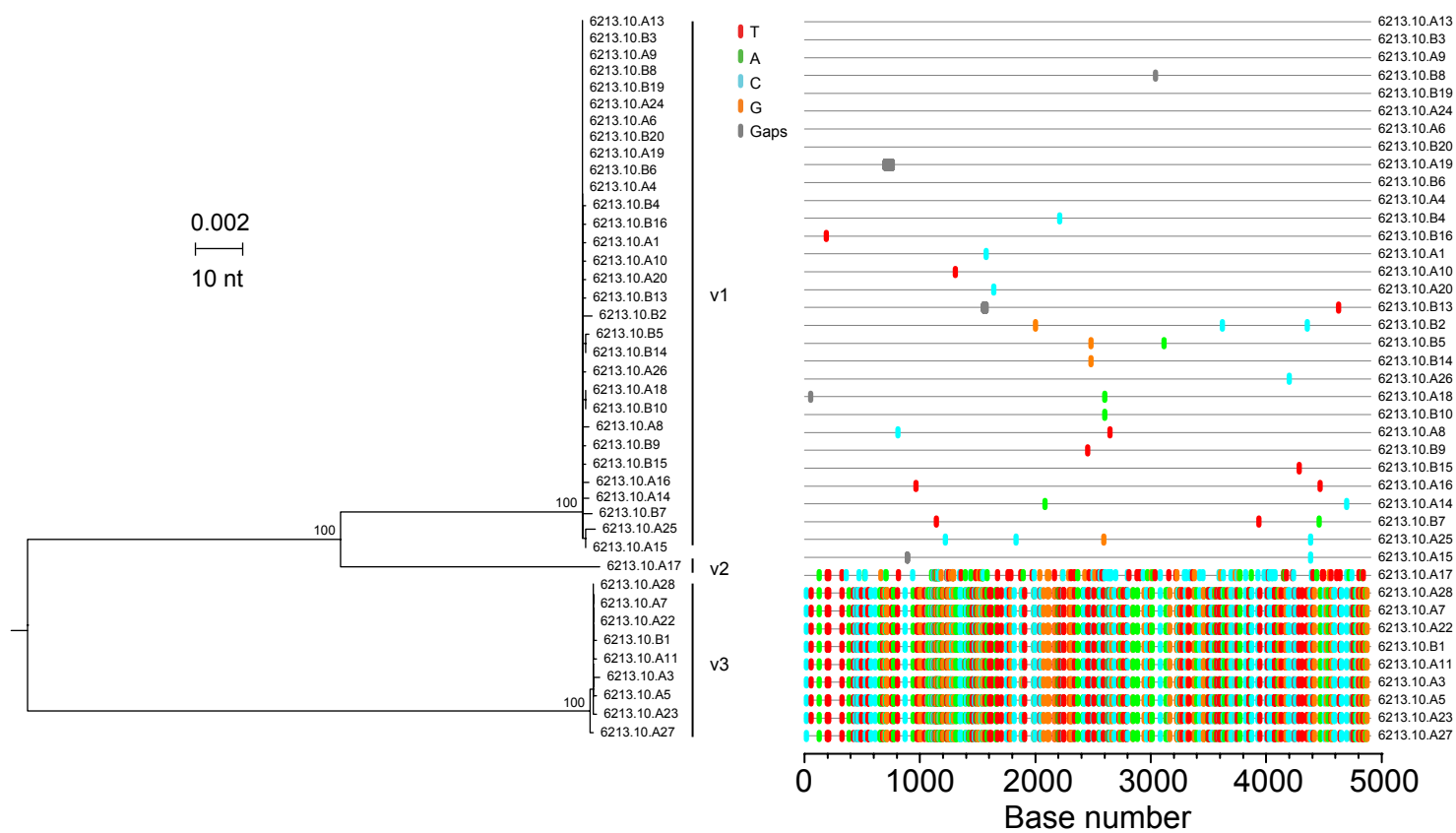

Figure S4

Supplement: Figure S4 — HCV diversity in acute subject 6213. 5′ half genomesequences are represented in a ML tree and Highlighter plot. Sequences show evidence of productive clinical infection by at least 3 T/F viruses. Bootstrap values are indicated and represent 100 repetitions. The horizontal scale bar indicates genetic distance. (PDF) [file ppat.1002880.s004.pdf]

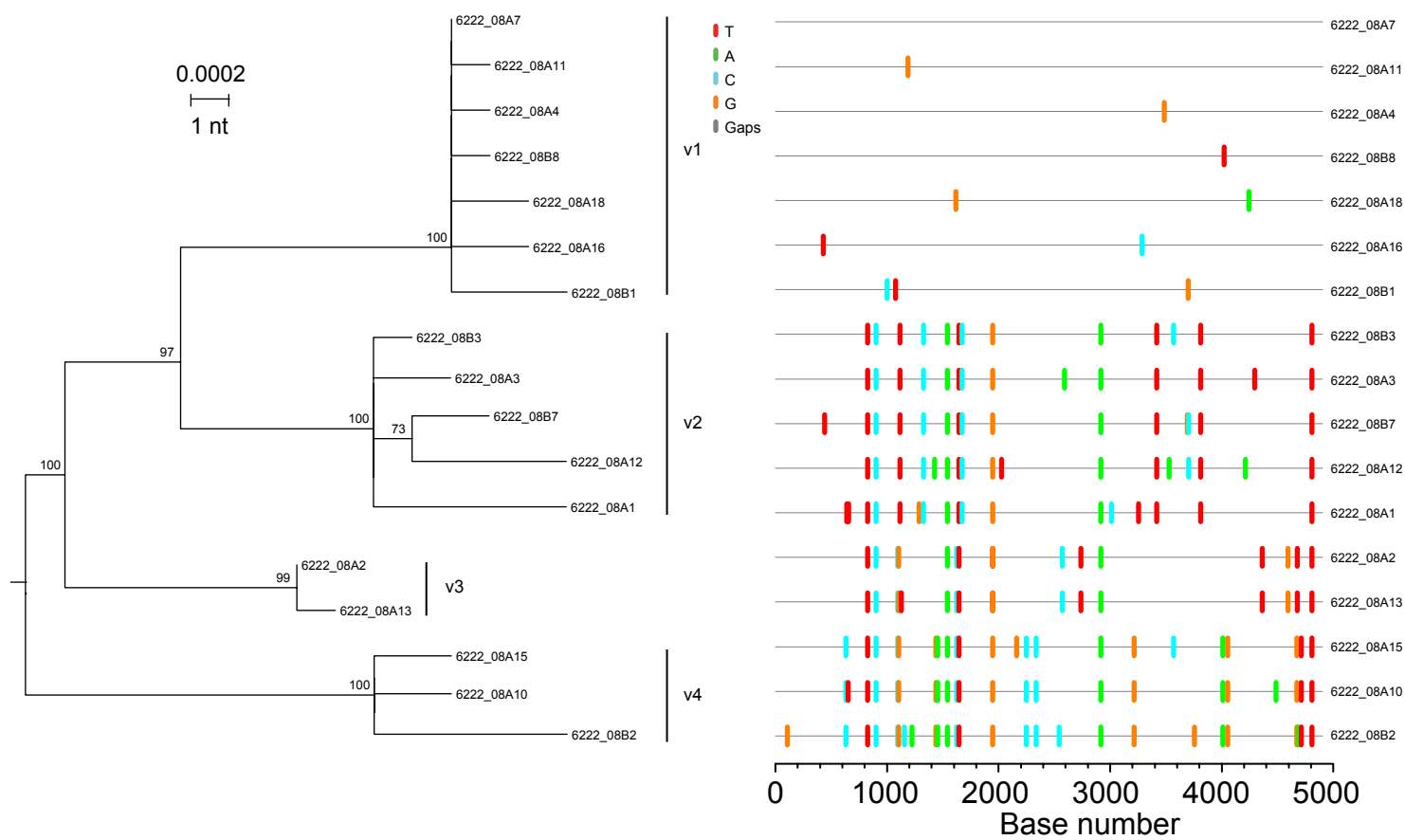

Figure S5

Supplement: Figure S5 — HCV diversity in acute subject 6222. 5′ half genomesequences are represented in a ML tree and Highlighter plot. Sequences show evidence of productive clinical infection by at least 4 T/F viruses. Bootstrap values are indicated and represent 100 repetitions. The horizontal scale bar indicates genetic distance. (PDF) [file ppat.1002880.s005.pdf]

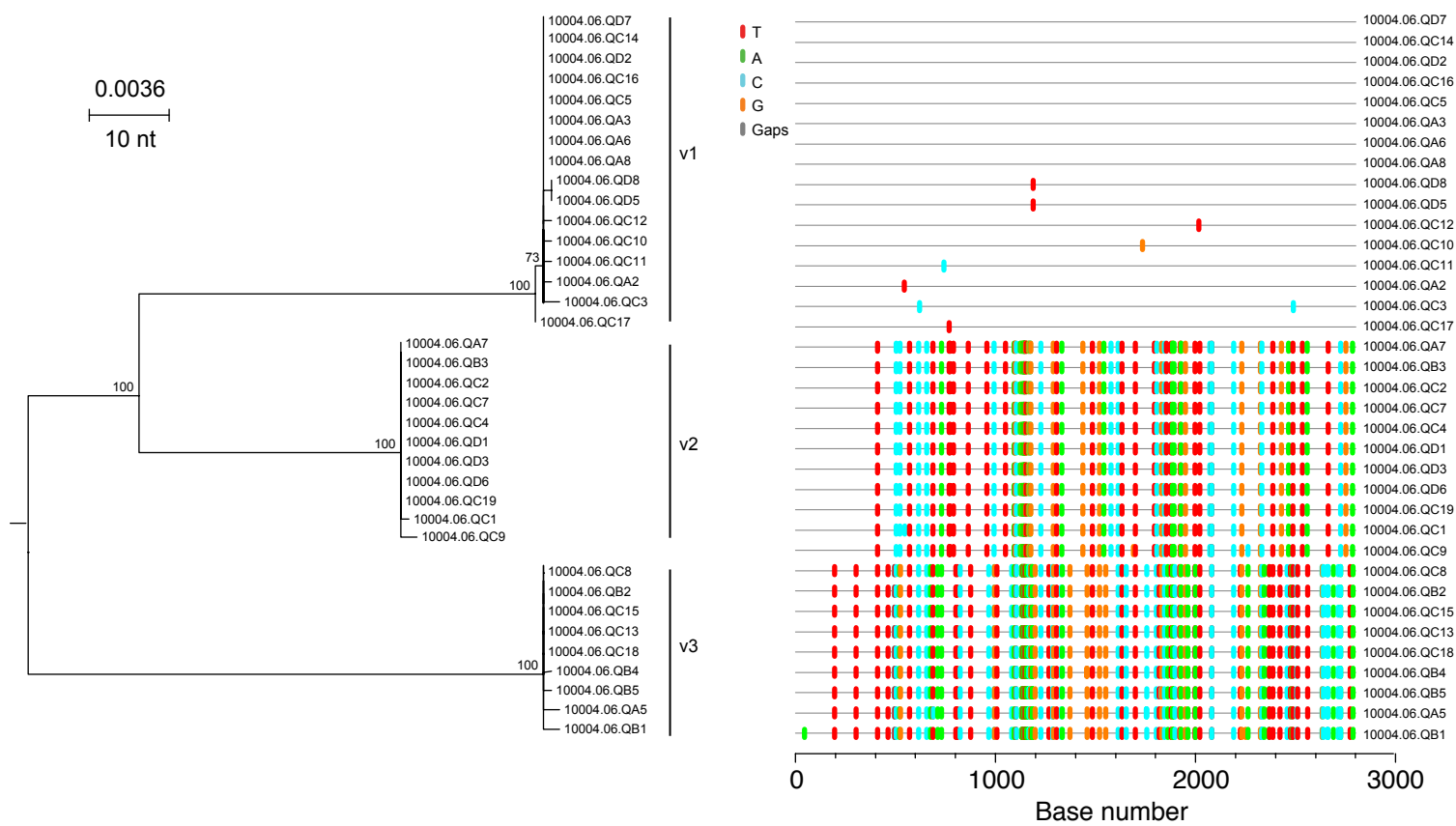

Figure S6

Supplement: Figure S6 — HCV diversity in acute subject 10004. 5′ quarter1 genomesequences are represented in a ML tree and Highlighter plot. Sequences show evidence of productive clinical infection by at least 3 T/F viruses. Bootstrap values are indicated and represent 100 repetitions. The horizontal scale bar indicates genetic distance. (PDF) [file ppat.1002880.s006.pdf]

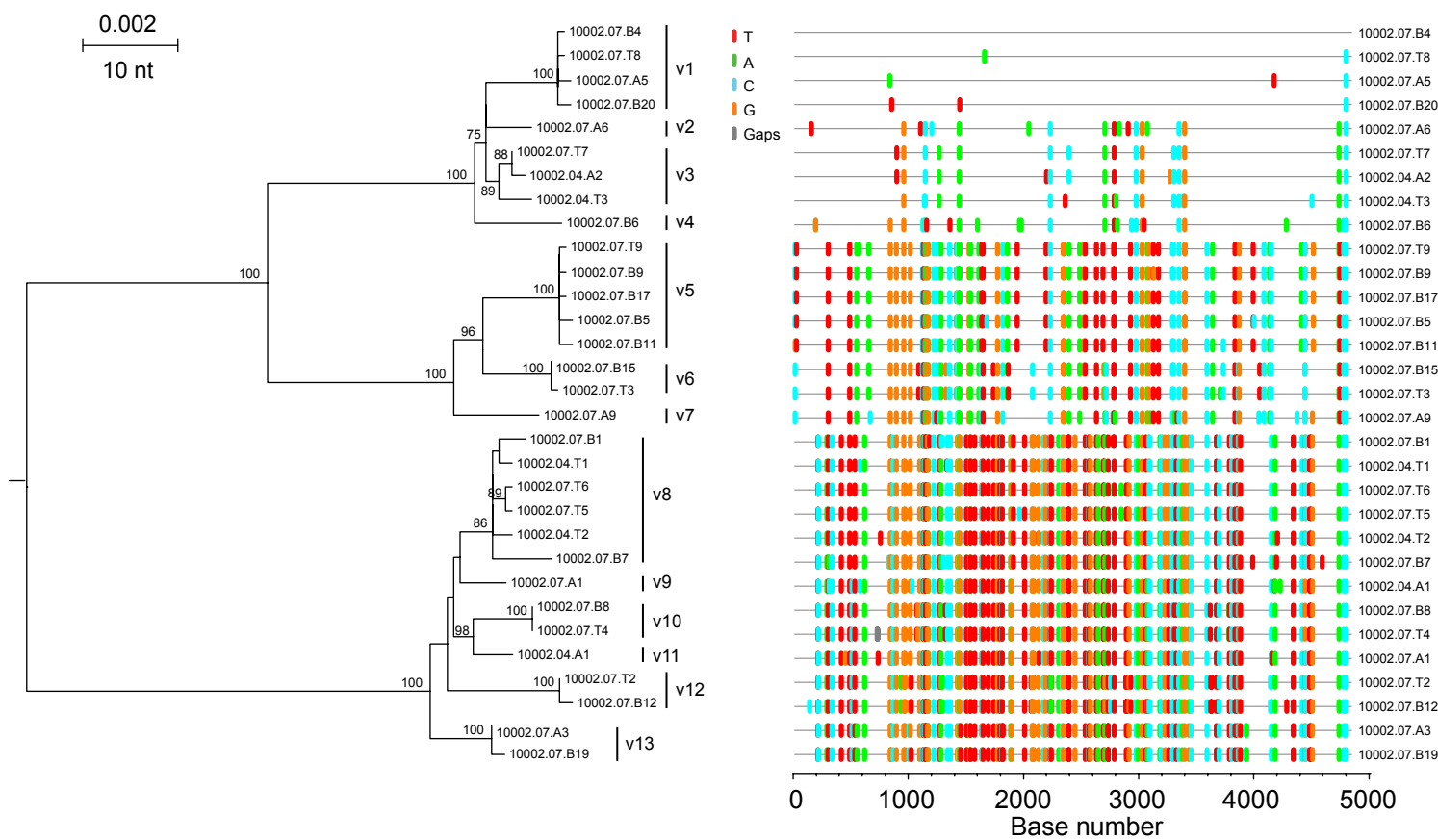

Figure S7

Supplement: Figure S7 — HCV diversity in acute subject 10002. 5′ half genomesequences are represented in a ML tree and Highlighter plot. Sequences show evidence of productive clinical infection by at least 13 T/F viruses. Bootstrap values are indicated and represent 100 repetitions. The horizontal scale bar indicates genetic distance. (PDF) [file ppat.1002880.s007.pdf]

**A**

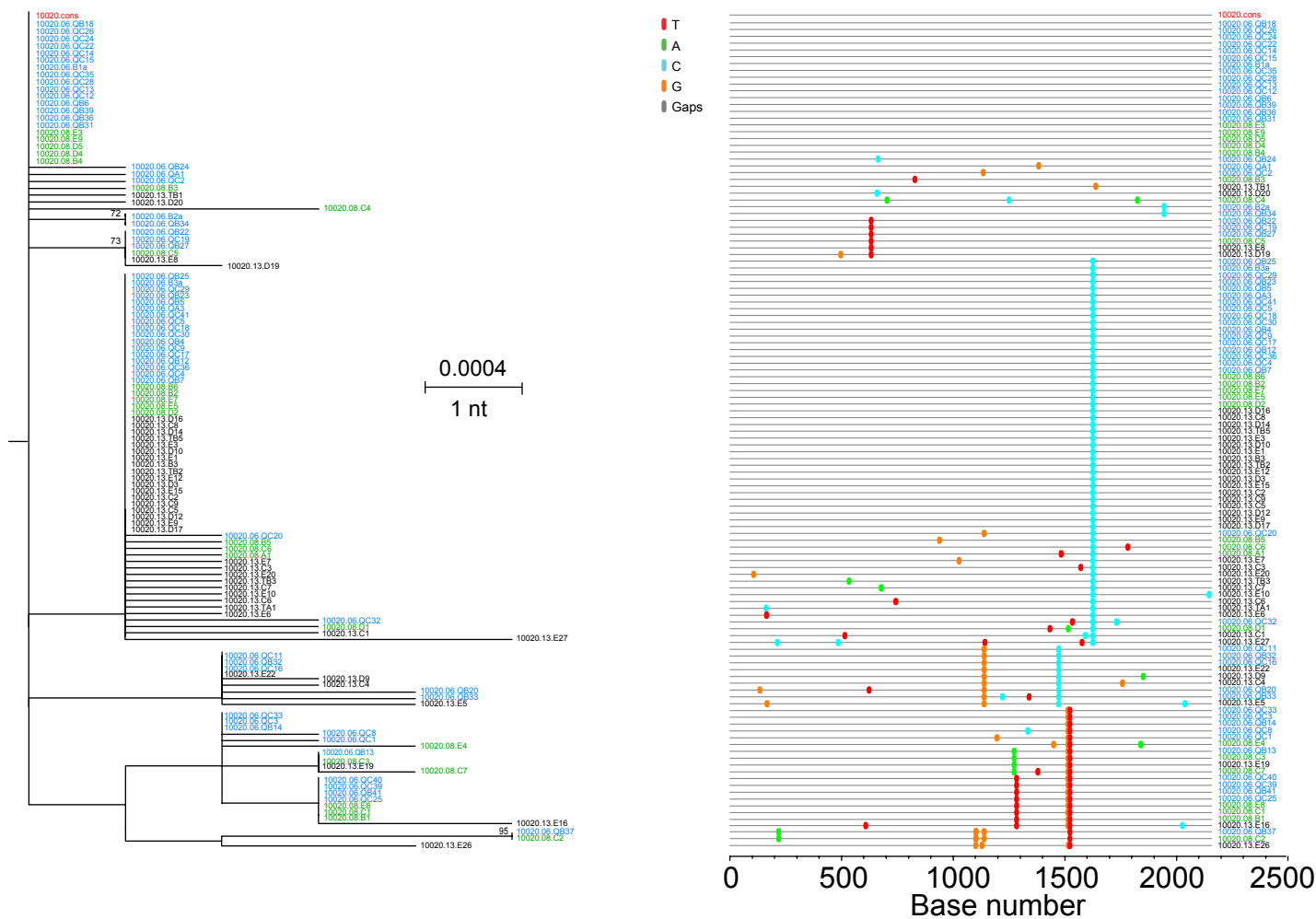

**B**

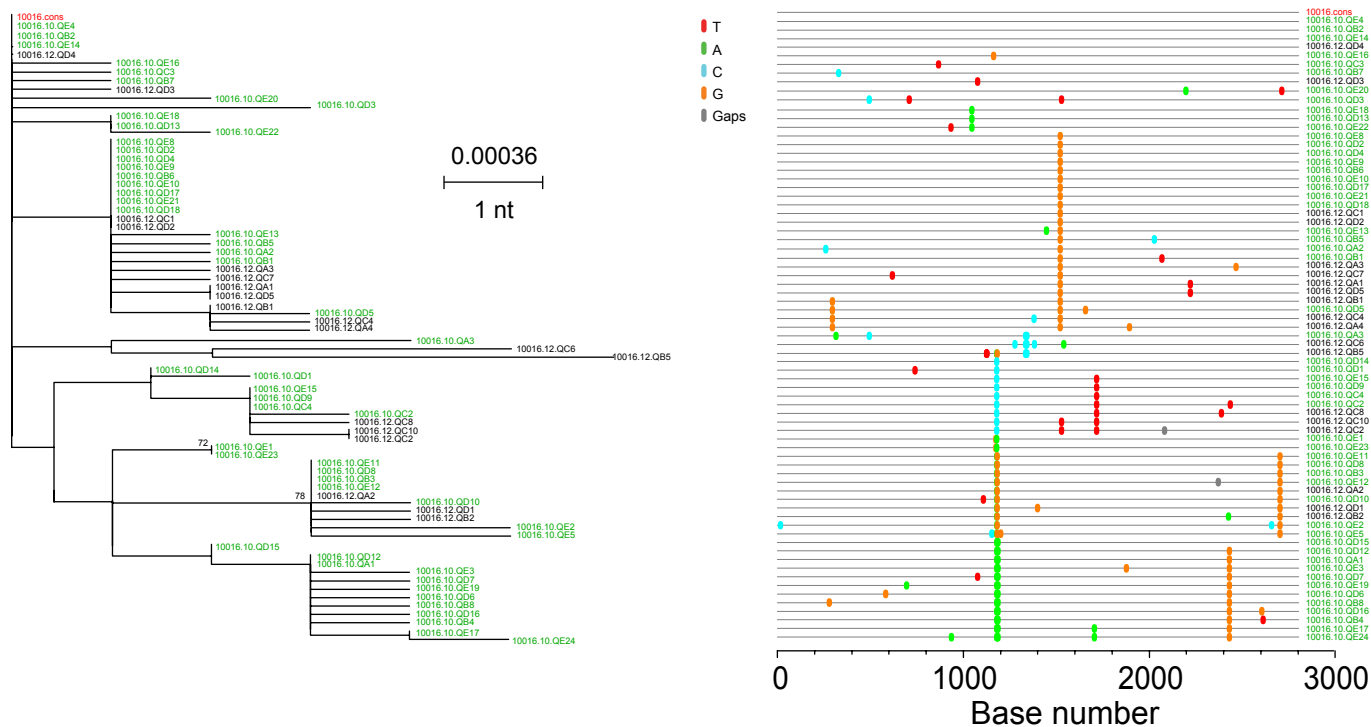

Figure S9

Supplement: Figure S9 — HCV diversity in acute subjects 10020 and 10016. 5′ quarter1 genome sequences from subject 10020 (panel A) and 10016 (panel B) are depicted by ML tree and Highlighter plots. Many sets of closely related sequences distinguished by unique shared mutations are evident. Bootstrap values represent 100 repetitions. (PDF) [file ppat.1002880.s009.pdf]

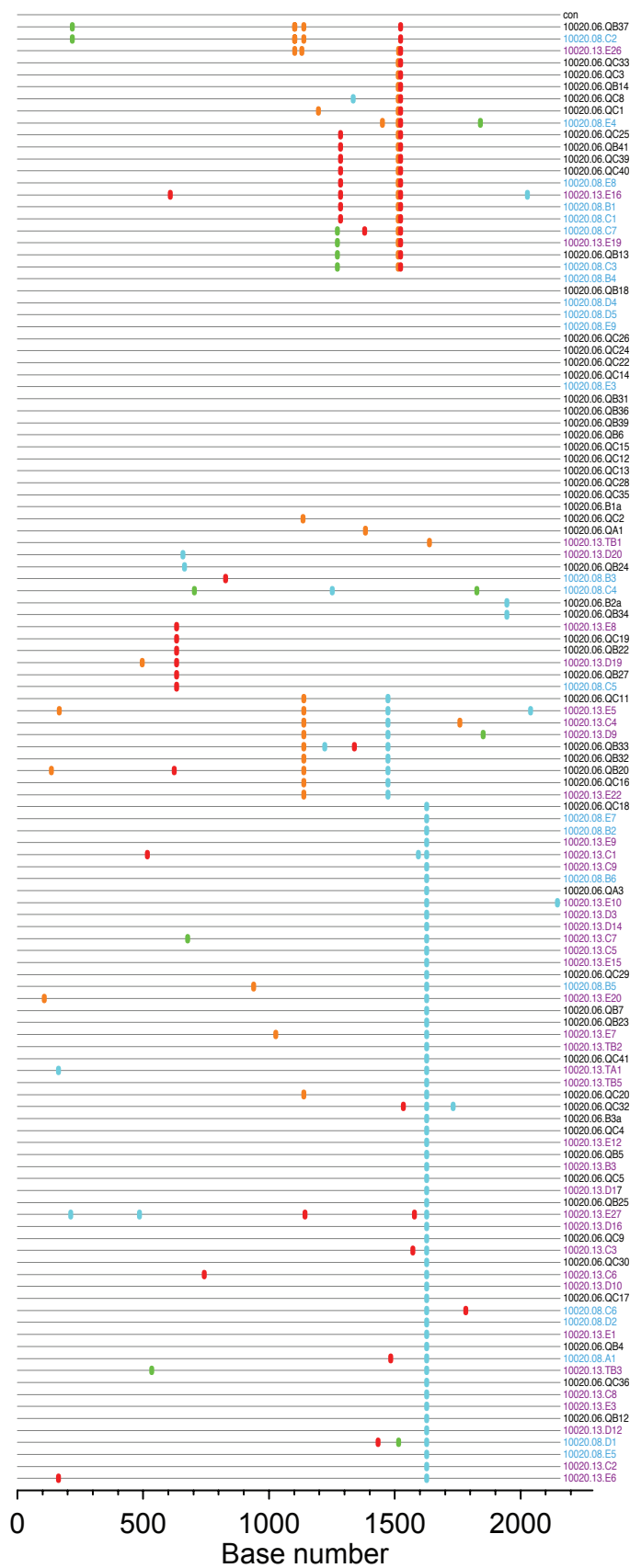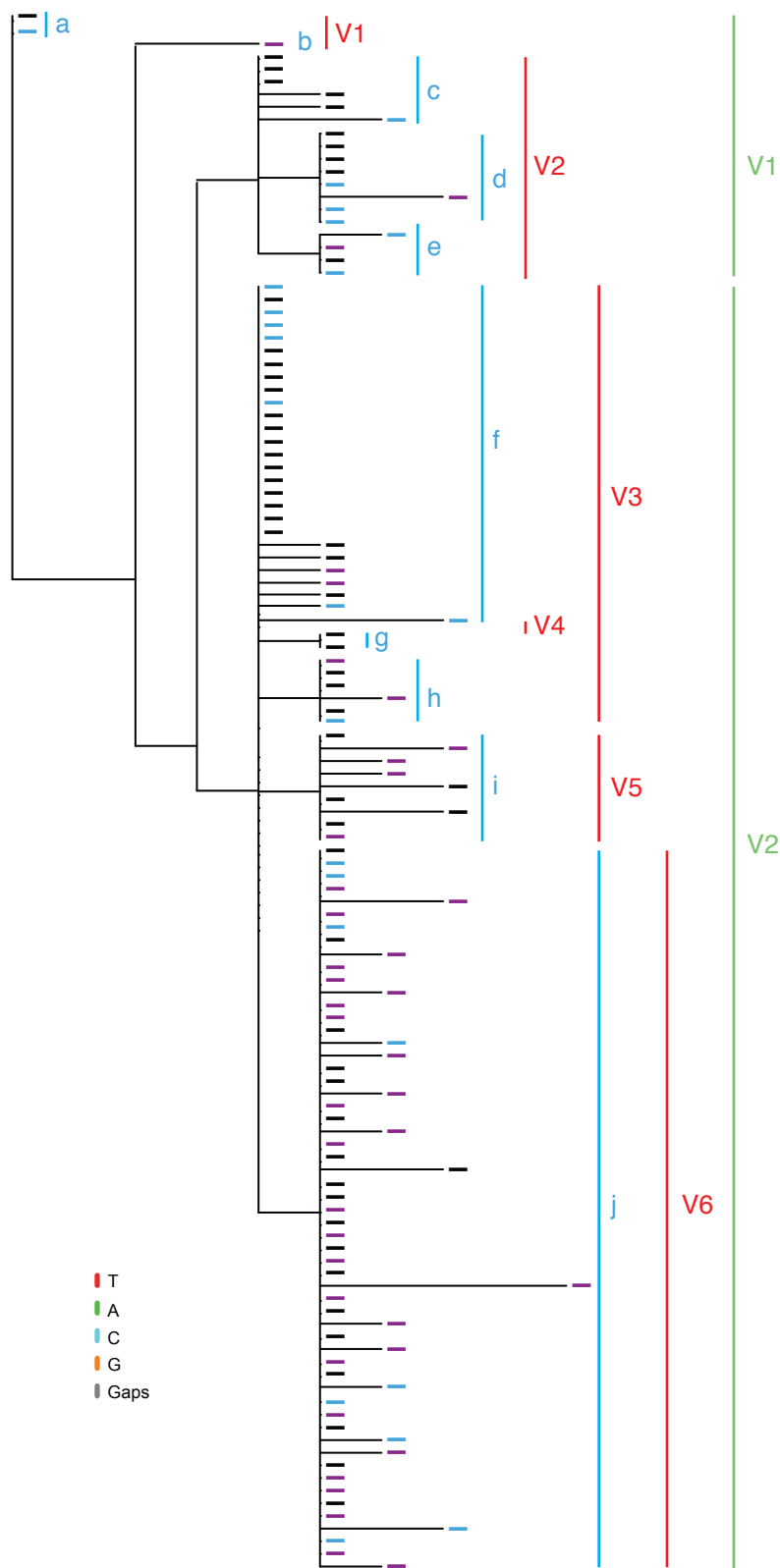

Figure S14

Supplement: Figure S14 — HCV diversity analysis in subject 10020 suggests acute-to-acute transmission. Highlighter plot and neighbor-joining tree of 5′ quarter 1 genome sequences. Visualization of 10 potential T/F viral sequences distinguished by unique shared mutations is indicated by lower case (blue) letters. Model estimates of T/F virus lineages using maximum (red) and average (green) cut-offs reveals 5 and 3 potential T/F virus lineages, respectively, based on increasingly stringent model assumptions (see text). (PDF) [file ppat.1002880.s014.pdf]

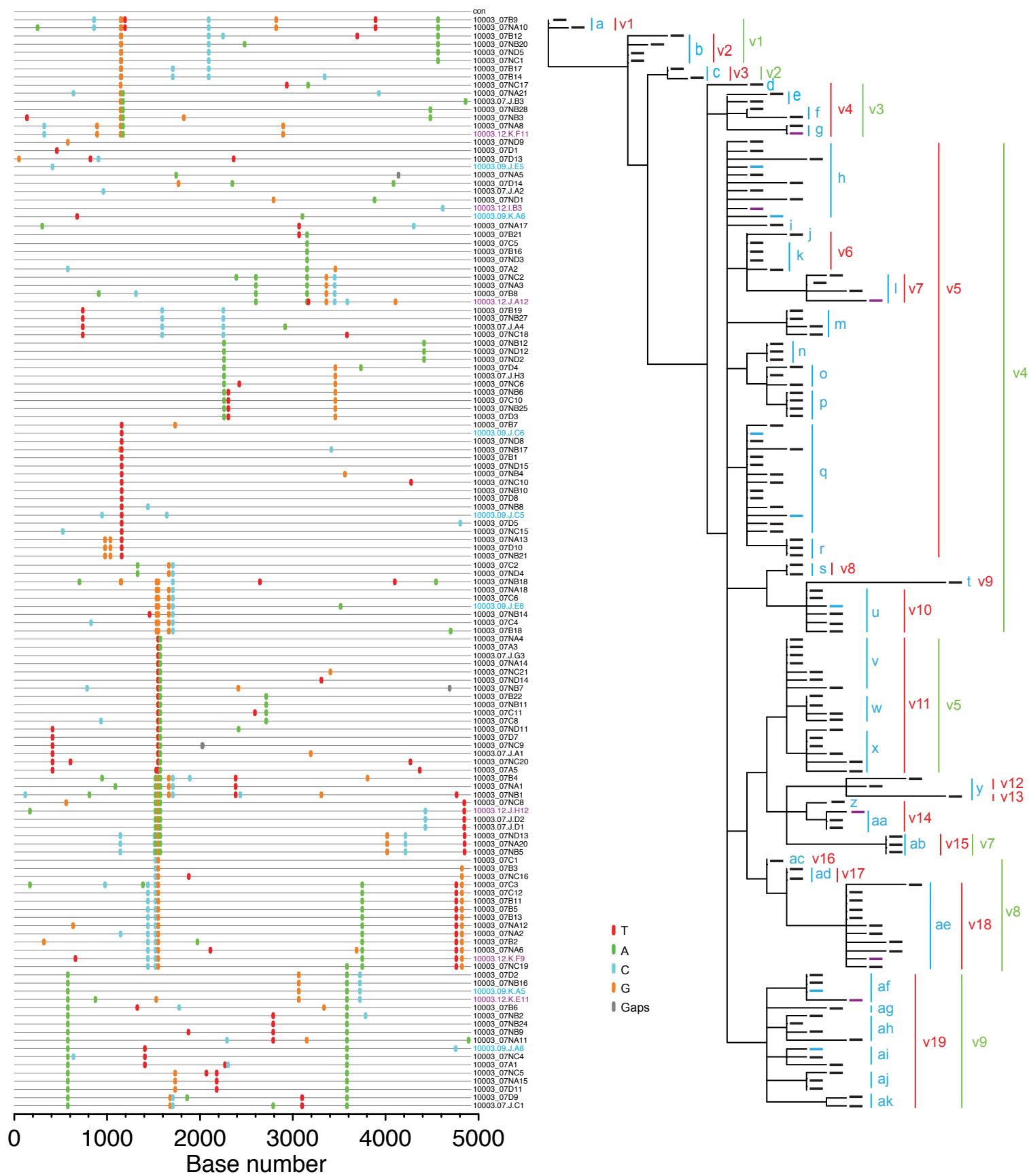

Figure S15

Supplement: Figure S15 — HCV diversity analysis in subject 10003 suggests acute-to-acute transmission. Highlighter plot and neighbor-joining tree of 5′ half genome sequences. Visualization of 37 potential T/F viral sequences distinguished by unique shared mutations is indicated by lower case (blue) letters. Model estimates of T/F virus lineages using maximum (red) and average (green) cut-offs reveals 15 and 8 potential T/F virus lineages, respectively, based on increasingly stringent model assumptions (see text). (PDF) [file ppat.1002880.s015.pdf]

A

B

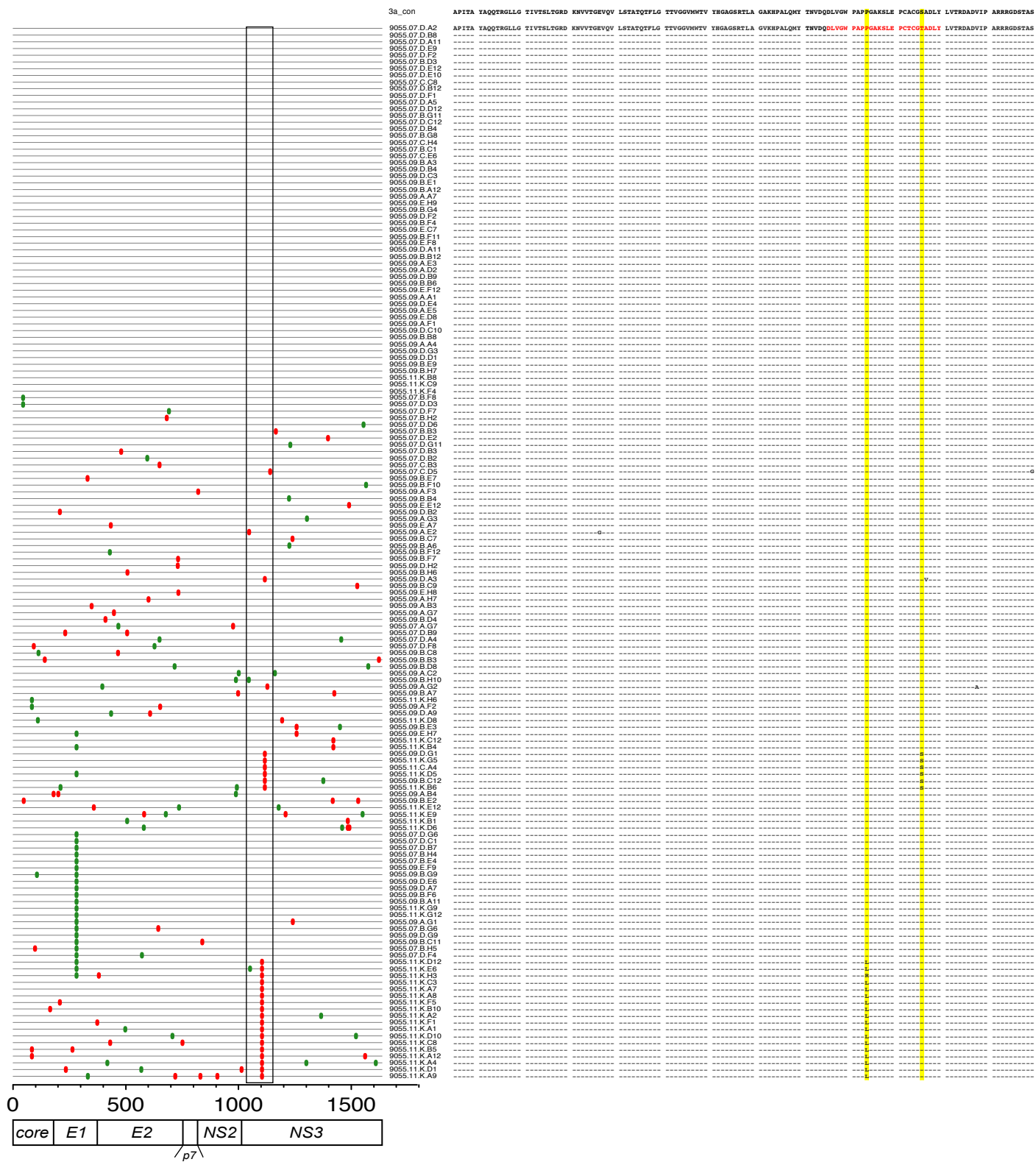

Figure S17

Supplement: Figure S17 — Nonsynonymous and synonymous mutations in HCV sequences from acute subject 9055. A Highlighter plot (panel A) of 5′ half genomesequences is color coded to denote nonynonymous (red) and synonymous (green) mutations. The boxed area reveals a temporal expansion of sequences with concentrated amino acid subtitutions in NS3. In panel B, amino acid selection is evident in a previously identified CTL epitope highlighted in red. The top-most sequence represents the genotype 3a consensus. (PDF) [file ppat.1002880.s017.pdf]
