# Supplementary material for: Elucidation of Hepatitis C Virus Transmission and Early Diversification by Single Genome Sequencing
Source: PLoS Pathog. 2012 Aug 23;8(8):e1002880. doi: 10.1371/journal.ppat.1002880 (PMC3426529; doi:10.1371/journal.ppat.1002880)
Supplement: Figure S11 — Amino acid alignment of the HCV Env coding region of acute subject 10003. The H77 reference sequence is shown at the top. Nonrandom concentrations of nonsynonymous mutations are evident. (PDF) [file ppat.1002880.s011.pdf]

```
                E2
               HVR1                               HVR2       CD81-1                                                     CD81-2
H77            ETHVTGGNAG RTTAGLVGLL TPGAKQNIQL....QGWGPISYAN -GSGLDERPY CWHYPPRPCG IVPAKSVCGP....VGTTDRSGAP TYSWGANDTD VFVLNNTRPP LGNWFGCTWM ...APPCVI-GGV GNNT----LL

CON            TTYATGGALG HSVFSFTSFL SVGPRQNLQL....QGWGPLTDAN ITGPSDDKPY CWHYAPRPCG VVEASRVCGP....VGTTDAKGVP TYTWGENKTD VFLLESLRPP GGQWFGCTWM ...APPCNIYGGK GDSTNQSDLF
10003_07NA5    ---------- ---------- ----------    ---------- ---------- ---------- ----------    ---------- ---------- ---------- ----------    ---------- ----------
10003_07D1     ---------- ---------- ----------    ---------- ---------- ---------- ----------    ---------- ---------- ---------- ----------    ---------- ----------
10003_07ND9    ---------- ---------- ----------    ---------- ---------- ---------- ----------    ---------- ---------- ---------- ----------    ---------- ----------
10003.07.J.A2  ---------- ---------- ----------    ---------- ---------- ---------- ----------    ---------- ---------- ---------- ----------    ---------- ----------
10003.09.J.E5  ---------- ---------- ----------    ---------- ---------- ---------- ----------    ---------- ---------- ---------- ----------    ---------- ----------
10003.12.I.B3  ---------- ---------- ----------    ---------- ---------- ---------- ----------    ---------- ---------- ---------- ----------    ---------- ----------
10003_07ND1    ---------- ---------- ----------    ---------- ---------- ---------- ----------    ---------- ---------- ---------- ----------    ---------- ----------
10003.09.K.A6  ---------- ---------- ----------    ---------- ---------- ---------- ----------    ---------- ---------- ---------- ----------    ---------- ----------
10003_07D14    ---------- ---------- ----------    ---------- ---------- ---------- ----------    ---------- ---------- ---------- ----------    ---------- ----------
10003_07D13    ---------- ---------- ----------    ---------- ---------- ---------- ----------    ---------- ---------- ---------- ----------    ---------- ----------
10003_07ND15   ---------- ---------- ----------    ---------- ---------- ---------- ----------    ---------- ---------- ---------- ----------    ---------- ----------
10003_07ND8    ---------- ---------- ----------    ---------- ---------- ---------- ----------    ---------- ---------- ---------- ----------    ---------- ----------
10003_07D8     ---------- ---------- ----------    ---------- ---------- ---------- ----------    ---------- ---------- ---------- ----------    ---------- ----------
10003_07B1     ---------- ---------- ----------    ---------- ---------- ---------- ----------    ---------- ---------- ---------- ----------    ---------- ----------
10003_07NB10   ---------- ---------- ----------    ---------- ---------- ---------- ----------    ---------- ---------- ---------- ----------    ---------- ----------
10003.09.J.C6  ---------- ---------- ----------    ---------- ---------- ---------- ----------    ---------- ---------- ---------- ----------    ---------- ----------
10003_07NB4    ---------- ---------- ----------    ---------- ---------- ---------- ----------    ---------- ---------- ---------- ----------    ---------- ----------
10003_07B7     ---------- ---------- ----------    ---------- ---------- ---------- ----------    ---------- ---------- ---------- ----------    ---------- ----------
10003_07NB8    ---------- ---------- ----------    ---------- ---------- ---------- ----------    ---------- ---------- ---------- ----------    ---------- ----------
10003_07NC15   ---------- ---------- ----------    ---------- ---------- ---------- ----------    ---------- ---------- ---------- ----------    ---------- ----------
10003_07NC10   ---------- ---------- ----------    ---------- ---------- ---------- ----------    ---------- ---------- ---------- ----------    ---------- ----------
10003_07D5     ---------- ---------- ----------    ---------- ---------- ---------- ----------    ---------- ---------- ---------- ----------    ---------- ----------
10003.09.J.C5  ---------- ---------- ----------    ---------- ---------- ---------- ----------    ---------- ---------- ---------- ----------    ---------- ----------
10003_07NB17   ---------- ----R----- ----------    ---------- ---------- ---------- ----------    ---------- ---------- ---------- ----------    ---------- ----------
10003_07NA13   ---------- ---------- ----------    ---------- ---------- ---------- ----------    ---------- ---------- ---------- ----------    ---------- ----------
10003_07D10    ---------- ---------- ----------    ---------- ---------- ---------- ----------    ---------- ---------- ---------- ----------    ---------- ----------
10003_07NB21   ---------- ---------- ----------    ---------- ---------- ---------- ----------    ---------- ---------- ---------- ----------    ---------- ----------
10003_07NB12   ---------- ---------- ----------    ---------- ---------- ---------- ----------    ---------- ---------- ---------- ----------    ---------- ----------
10003_07ND12   ---------- ---------- ----------    ---------- ---------- ---------- ----------    ---------- ---------- ---------- ----------    ---------- ----------
10003_07ND2    ---------- ---------- ----------    ---------- ---------- ---------- ----------    ---------- ---------- ---------- ----------    ---------- ----------
10003.07.J.H3  ---------- ---------- ----------    ---------- ---------- ---------- ----------    ---------- ---------- ---------- ----------    ---------- ----------
10003_07D4     ---------- ---------- ----------    ---------- ---------- ---------- ----------    ---------- ---------- ---------- ----------    ---------- ----------
10003_07NC6    ---------- ---------- ----------    ---------- ---------- ---------- ----------    ---------- ---------- ---------- ----------    ---------- ----------
10003_07NB6    ---------- ---------- ----------    ---------- ---------- ---------- ----------    ---------- ---------- ---------- ----------    ---------- ----------
10003_07C10    ---------- ---------- ----------    ---------- ---------- ---------- ----------    ---------- ---------- ---------- ----------    ---------- ----------
10003_07NB25   ---------- ---------- ----------    ---------- ---------- ---------- ----------    ---------- ---------- ---------- ----------    ---------- ----------
10003_07D3     ---------- ---------- ----------    ---------- ---------- ---------- ----------    ---------- ---------- ---------- ----------    ---------- ----------
10003_07B19    ---------- ---------- ----------    ---------- ---------- ---------- ----------    ---------- ---------- ---------- ----------    ---------- ----------
10003_07NB27   ---------- ---------- ----------    ---------- ---------- ---------- ----------    ---------- ---------- ---------- ----------    ---------- ----------
10003_07NC18   ---------- ---------- ----------    ---------- ---------- ---------- ----------    ---------- ---------- ---------- ----------    ---------- ----------
10003.07.J.A4  ---------- ---------- ----------    ---------- ---------- ---------- ----------    ---------- ---------- ---------- ----------    ---------- ----------
10003_07ND3    ---------- ---------- ----------    ---------- ---------- ---------- ----------    ---------- ---------- ---------- ----------    ---------- ----------
10003_07C5     ---------- ---------- ----------    ---------- ---------- ---------- ----------    ---------- ---------- ---------- ----------    ---------- ----------
10003_07B16    ---------- ---------- ----------    ---------- ---------- ---------- ----------    ---------- ---------- ---------- ----------    ---------- ----------
10003_07A2     ---------- ---------- ----------    ---------- ---------- ---------- ----------    ---------- ---------- ---------- ----------    ---------- ----------
10003_07B21    ---------- ---------- ----------    ---------- ---------- ---------- ----------    ---------- ---------- ---------- ----------    ---------- ----------
10003_07NA17   ---------- ---------- ----------    ---------- ---------- ---------- ----------    ---------- ---------- ---------- ----------    ---------- ----------
10003_07NA3    ---------- ---------- ----------    ---------- ---------- ---------- ----------    ---------- ---------- ---------- ----------    ---------- ----------
10003_07NC2    ---------- ---------- ----------    ---------- ---------- ---------- ----------    ---------- ---------- ---------- ----------    ---------- ----------
10003_07B8     ---------- ---------- ----------    ---------- ---------- ---------- ----------    ---------- ---------- ---------- ----------    ---------- ----------
10003.12.J.A12 ---------- ---------- ----------    ---------- ---------- ---------- ----------    ---------- ---------- ---------- ----------    ---------- ----------
10003_07NC4    ---------- ---------- ----------    ---------- ---------- ---------- ----------    ---------- ---------- ---------- ----------    ---------- ----------
10003.09.J.A8  ---------- ---------- ----------    ---------- ---------- ---------- ----------    ---------- ---------- ---------- ----------    ---------- ----------
10003_07A1     ---------- ---------- ----------    ---------- ---------- ---------- ----------    ---------- ---------- ---------- ----------    ---------- ----------
10003_07D11    ---------- ---------- ----------    ---------- ---------- ---------- ----------    ---------- ---------- ---------- ----------    ---------- ----------
10003_07NA15   ---------- ---------- ----------    ---------- ---------- ---------- ----------    ---------- ---------- ---------- ----------    ---------- ----------
10003_07NC5    ---------- ---------- ----------    ---------- ---------- ---------- ----------    ---------- ---------- ---------- ----------    ---------- ----------
10003_07B6     ---------- ---------- ----------    ---------- ---------- ---------- ----------    ---------- ---------- ---------- ----------    ---------- ----------
10003_07NB24   ---------- ---------- ----------    ---------- ---------- ---------- ----------    ---------- ---------- ---------- ----------    ---------- ----------
10003_07NB9    ---------- ---------- ----------    ---------- ---------- ---------- ----------    ---------- ---------- ---------- ----------    ---------- ----------
10003_07NB2    ---------- ---------- ----------    ---------- ---------- ---------- ----------    ---------- ---------- ---------- ----------    ---------- ----------
10003_07NA11   ---------- ---------- ----------    ---------- ---------- ---------- ----------    ---------- ---------- ---------- ----------    ---------- ----------
10003_07NB16   ---------- ---------- ----------    ---------- ---------- ---------- ----------    ---------- ---------- ---------- ----------    ---------- ----------
10003_07D2     ---------- ---------- ----------    ---------- ---------- ---------- ----------    ---------- ---------- ---------- ----------    ---------- ----------
10003.09.K.A5  ---------- ---------- ----------    ---------- ---------- ---------- ----------    ---------- ---------- ---------- ----------    ---------- ----------
10003.12.K.E11 ---------- ---------- ----------    ---------- ---------- ---------- ----------    ---------- ---------- ---------- ----------    ---------- ----------
10003_07D9     ---------- ---------- ----------    ---------- ---------- ---------- ----------    ---------- ---------- ---------- ----------    ---------R ---------L
10003.07.J.C1  ---------- ---------- ----------    ---------- ---------- ---------- ----------    ---------- ---------- ---------- ----------    ---------R ---------L
10003_07NC17   ---------- ------A--- ----------    ---------- ---------- ---------- ----------    ---------- ---------- ---------- ----------    ---------- ----------
10003.07.J.B3  ---------- ------A--- ----K-----    ---------- ---------- ---------- ----------    ---------- ---------- ---------- ----------    ---------- ----------
10003_07NA21   ---------- ------A--- ----K-----    ---------- ---------- ---------- ----------    ---------- ---------- ---------- ----------    ---------- ----------
10003_07NB28   ---------- ------A--- ----K-----    ---------- ---------- ---------- ----------    ---------- ---------- ---------- ----------    ---------- ----------
10003_07NB3    ---------- ------A--- ----K-----    ---------- ---------- ---------- ----------    ---------- ---------- ---------- ----------    ---------- ----------
10003_07NA8    ---------- ------A--- ----K-----    ---------- ---------- ---------- ----------    ---------- ---------- ---------- ----------    ---------- ----------
10003.12.K.F11 ---------- ------A--- ----K-----    ---------- ---------- ---------- ----------    ---------- ---------- ---------- ----------    ---------- ----------
10003_07B17    ---------- ------AR-- ----------    ---------- ---------- ---------- ----------    ---------- ---------- ---------- ----------    ---------- ---------L
10003_07B14    ---------- ------AR-- ----------    ---------- ---------- ---------- ----------    ---------- ---------- ---------- ----------    ---------- ---------L
10003_07NC1    ---------- ------AR-- ----------    ---------- ---------- ---------- ----------    ---------- ---------- ---------- ----------    ---------- ----------
10003_07ND5    ---------- ------AR-- ----------    ---------- ---------- ---------- ----------    ---------- ---------- ---------- ----------    ---------- ----------
10003_07NB20   ---------- ------AR-- ----------    ---------- ---------- ---------- ----------    ---------- ---------- ---------- ----------    ---------- ----------
10003_07B12    ---------- ------AR-- ----------    ---------- ---------- ---------- ----------    ---------- ---------- ---------- ----------    ---------- ----------
10003_07B9     ---------- ------AR-- ----------    ---------- ---------- ---------- ----------    ---------- ---------- ---------- ----------    ---------- ----------
10003_07NA10   ---------- ------AR-- ----------    ---------- ---------- ---------- ----------    ---------- ---------- ---------- ----------    ---------- ----------
10003_07NB18   ---------- ------AR-- ----------    ---------- ---------- ---------- ----------    ---------- --A----E-- ---------- ----------    ---------- ---------L
10003_07C2     ---------- ---------- ----------    ---------- ---------- ---------- ----------    ---------- ---------- ---------- ----------    ----D----- ---------L
10003_07ND4    ---------- ---------- ----------    ---------- ---------- ---------- ----------    ---------- ---------- ---------- ----------    ----D----- ---------L
10003_07NA18   ---------- ---------- ----------    ---------- ---------- ---------- ----------    ---------- --A----E-- ---------- ----------    ----D----- ---------L
10003_07C6     ---------- ---------- ----------    ---------- ---------- ---------- ----------    ---------- --A----E-- ---------- ----------    ----D----- ---------L
10003_07C4     ---------- ---------- ----------    ---------- ---------- ---------- ----------    ---------- --A----E-- ---------- ----------    ----D----- ---------L
10003_07B18    ---------- ---------- ----------    ---------- ---------- ---------- ----------    ---------- --A----E-- ---------- ----------    ----D----- ---------L
10003_07NB14   ---------- ---------- ----------    ---------- ---------- ---------- ----------    ---------- --A----E-- ---------- ----------    ----D----- ---------L
10003.09.J.E6  ---------- ---------- ----------    ---------- ---------- ---------- ----------    ---------- --A----E-- ---------- ----------    ----D----- ---------L
10003_07NA14   ---------- ---------- ----------    ---------- ---------- ---------- ----------    ---------- -------D-- ----K----- ----------    ---------- ----------
10003_07A3     ---------- ---------- ----------    ---------- ---------- ---------- ----------    ---------- -------D-- ----K----- ----------    ---------- ----------
10003_07NA4    ---------- ---------- ----------    ---------- ---------- ---------- ----------    ---------- -------D-- ----K----- ----------    ---------- ----------
10003.07.J.G3  ---------- ---------- ----------    ---------- ---------- ---------- ----------    ---------- -------D-- ----K----- ----------    ---------- ----------
10003_07NC21   ---------- ---------- ----------    ---------- ---------- ---------- ----------    ---------- -------D-- ----K----- ----------    ---------- ----------
10003_07ND14   ---------- ---------- ----------    ---------- ---------- ---------- ----------    ---------- -------D-- ----K----- ----------    ---------- ----------
10003_07NB7    ---------- ---------- ----------    ---------- ---------- ---------- ----------    ---------- -------D-- ----K----- ----------    ---------- ----------
10003_07B22    ---------- ---------- ----------    ---------- ---------- ---------- ----------    ---------- -------D-- ----K----- ----------    ---------- ----------
10003_07NB11   ---------- ---------- ----------    ---------- ---------- ---------- ----------    ---------- -------D-- ----K----- ----------    ---------- ----------
10003_07C11    ---------- ---------- ----------    ---------- ---------- ---------- ----------    ---------- -------D-- ----K----- ----------    ---------- ----------
10003_07C8     ---------- ---------- ----------    ---------- ---------- ---------- ----------    ---------- -------D-- ----K----- ----------    ---------- ----------
10003_07D7     ---------- ---------- ----------    ---------- ---------- ---------- ----------    ---------- -------D-- ----K----- ----------    ---------- ----------
10003_07NC9    ---------- ---------- ----------    ---------- ---------- ---------- ----------    ---------- -------D-- ----K----- ----------    ---------- ----------
10003_07ND11   ---------- ---------- ----------    ---------- ---------- ---------- ----------    ---------- -------D-- ----K----- ----------    ---------- ----------
10003.07.J.A1  ---------- ---------- ----------    ---------- ---------- ---------- ----------    ---------- -------D-- ----K----- ----------    ---------- ----------
10003_07NC20   ---------- ---------- ----------    ---------- ---------- ---------- ----------    ---------- -------D-- ----K----- ----------    ---------- ----------
10003_07A5     ---------- ---------- ----------    ---------- ---------- ---------- ----------    ---------- I------D-- ----K----- ----------    ---------- ----------
10003_07NC8    ---------- ---------- ----------    ---------- ---------- ---------- ----------    --------E- -------E-- ----K----- ----------    ---------- ----------
10003.07.J.D2  ---------- ---------- ----------    ---------- ---------- ---------- ----------    --------E- -------E-- ----K----- ----------    ---------- ----------
10003.07.J.D1  ---------- ---------- ----------    ---------- ---------- ---------- ----------    --------E- -------E-- ----K----- ----------    ---------- ----------
10003.12.J.H12 ---------- ---------- ----------    ---------- ---------- ---------- ----------    --------E- -------E-- ----K----- ----------    ---------- ----------
10003_07ND13   ---------- ---------- ----------    ---------- ---------- ---------- ----------    --------E- -------E-- ----K----- ----------    ---------- ----------
10003_07NA20   ---------- ---------- ----------    ---------- ---------- ---------- ----------    --------E- -------E-- ----K----- ----------    ---------- ----------
10003_07NB5    ---------- ---------- ----------    ---------- ---------- ---------- ----------    --------E- -------E-- ----K----- ----------    ---------- ----------
10003_07NA1    ---------- ---------- ----------    ---------- ---------- ---------- ----------    --------E- -------E-- ----K----- ----------    ----D----- ---------L
10003_07B4     ---------- ---------- ----------    ---------- ---------- ---------- ----------    --------E- -------E-- ----K----- ----------    ----D----- ---------L
10003_07NB1    ---------- ---------- ----------    ---------- ---------- ---------- ----------    --------E- -------E-- ----K----- ----------    ----D----- ---------L
10003_07C1     ---------- ---------- ----------    ---------- ---------- ---------- ----------    --------A- -------E-- ---------- ----------    ---------- ----------
10003_07B3     ---------- ---------- ----------    ---------- ---------- ---------- ----------    --------A- -------E-- ---------- ----------    ---------- ----------
10003_07NC16   ---------- ---------- ----------    ---------- ---------- ---------- ----------    --------A- -------E-- ---------- ----------    ---------- ----------
10003_07B13    ---------- ---------- ----------    ---------- ---------- ---------- A---------    --------A- -------E-- ---------- ----------    ---------- ----------
10003_07B11    ---------- ---------- ----------    ---------- ---------- ---------- A---------    --------A- -------E-- ---------- ----------    ---------- ----------
10003_07C12    ---------- ---------- ----------    ---------- ---------- ---------- A---------    --------A- -------E-- ---------- ----------    ---------- ----------
10003_07B5     ---------- ---------- ----------    ---------- ---------- ---------- A---------    --------A- -------E-- ---------- ----------    ---------- ----------
10003_07NC19   ---------- ---------- ----------    ---------- ---------- ---------- A---------    --------A- -------E-- ---------- ----------    ---------- ----------
10003_07NA12   ---------- ---------- ----------    ---------- ---------- ---------- A---------    --------A- -------E-- ---------- ----------    ---------- ----------
10003_07NA2    ---------- -----L---- ----------    ---------- ---------- ---------- A---------    --------A- -------E-- ---------- ----------    ---------- ----------
10003.12.K.F9  ---------- ---------- ----------    ---------- ---------- ---------- A---------    --------A- -------E-- ---------- ----------    ---------- ----------
10003_07B2     ---------- ---------- ----------    ---------- ---------- ---------- A---------    --------A- -------E-- ---------- ----------    ---------- ----------
10003_07NA6    ---------- ---------- ----------    ---------- ---------- ---------- A---------    --------A- -------E-- ---------- ----------    ---------- ----------
10003_07C3     ---------- ---------- ----------    ---------- --S------- ---------- A---------    --------A- -------E-- ---------- ----------    ---------- ----------
```

Figure S11
