# Supplementary material for: Elucidation of Hepatitis C Virus Transmission and Early Diversification by Single Genome Sequencing
Source: PLoS Pathog. 2012 Aug 23;8(8):e1002880. doi: 10.1371/journal.ppat.1002880 (PMC3426529; doi:10.1371/journal.ppat.1002880)
Supplement: Figure S18 — Polymerase template switching. Template switching by the RNA polymerase between minus sense and plus sense strands of double-stranded HCV RNA resulted in short stretches of perfect inverted repeat sequences. Five examples are illustrated in panels A–E. Proposed mechanisms for template switching are illustrated. (PDF) [file ppat.1002880.s018.pdf]

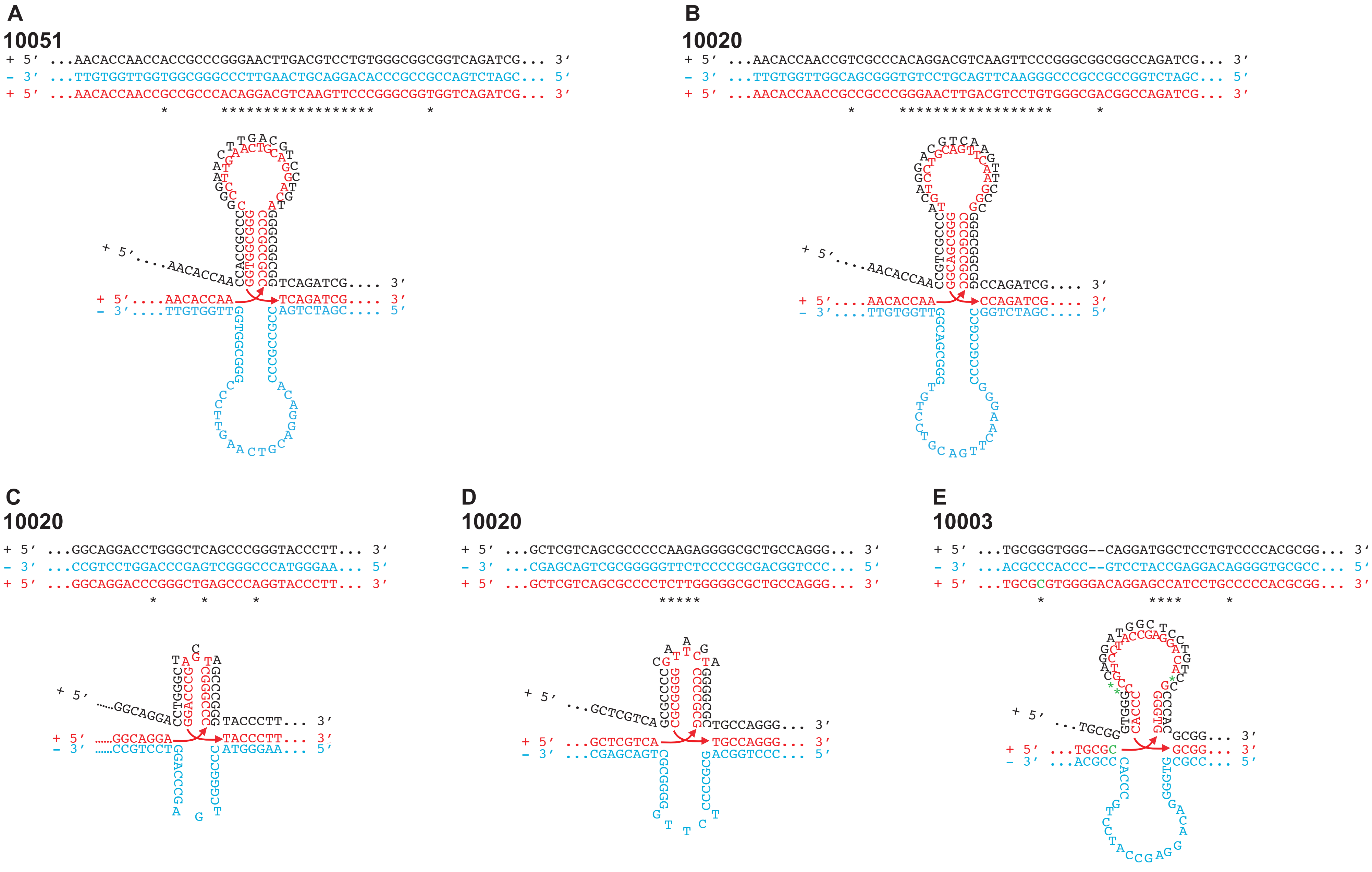
A
10051
+ 5′ ...AACACCAACCACCGCCCGGGAACTTGACGTCCTGTGGGCGGCGGTCAGATCG... 3′
- 3′ ...TTGTGGTTGGTGGCGGGCCCTTGAACTGCAGGACACCCGCCGCCAGTCTAGC... 5′
+ 5′ ...AACACCAACCGCCGCCCACAGGACGTCAAGTTCCCGGGCGGTGGTCAGATCG... 3′
B
10020
+ 5′ ...AACACCAACCGTCGCCCACAGGACGTCAAGTTCCCGGGCGGCGGCCAGATCG... 3′
- 3′ ...TTGTGGTTGGCAGCGGGTGTCCTGCAGTTCAAGGGCCCGCCGCCGGTCTAGC... 5′
+ 5′ ...AACACCAACCGCCGCCCGGGAACTTGACGTCCTGTGGGCGACGGCCAGATCG... 3′
C
10020
+ 5′ ...GGCAGGACCTGGGCTCAGCCCGGGTACCCTT... 3′
- 3′ ...CCGTCCTGGACCCGAGTCGGGCCCATGGGAA... 5′
+ 5′ ...GGCAGGACCCGGGCTGAGCCCAGGTACCCTT... 3′
D
10020
+ 5′ ...GCTCGTCAGCGCCCCCAAGAGGGGCGCTGCCAGGG... 3′
- 3′ ...CGAGCAGTCGCGGGGGTTCTCCCCGCGACGGTCCC... 5′
+ 5′ ...GCTCGTCAGCGCCCCTCTTGGGGGCGCTGCCAGGG... 3′
E
10003
+ 5′ ...TGCGGGTGGG--CAGGATGGCTCCTGTCCCCACGCGG... 3′
- 3′ ...ACGCCCACCC--GTCCTACCGAGGACAGGGGTGCGCC... 5′
+ 5′ ...TGCGCGTGGGGACAGGAGCCATCCTGCCCCCACGCGG... 3′

Figure S18
