# Supplementary material for: Elucidation of Hepatitis C Virus Transmission and Early Diversification by Single Genome Sequencing
Source: PLoS Pathog. 2012 Aug 23;8(8):e1002880. doi: 10.1371/journal.ppat.1002880 (PMC3426529; doi:10.1371/journal.ppat.1002880)
Supplement: Table S1 — Diversity analysis of HCV 5′ half genome sequences from 14 chronically infected subjects. (DOC) [file ppat.1002880.s019.doc]

| **Table S1. Diversity analysis of HCV 5' half genome sequences from 14 chronically infected subjects** | | | | | | | | | | | |
| --- | --- | --- | --- | --- | --- | --- | --- | --- | --- | --- | --- |
| **Sample ID** | **Sample date** | **Viral load (IU/ml)** | **Genotype** | | **# of 5' half  sequences** | | **Length** | | **Median diversity** | | **Range of  diversity** |
| WIMI4025 | 10/3/08 | 7,690,001 | 1a | 36 | | 4887 | | 1.21% | | 0.12 - 3.83% | |
| SLRO5563 | 9/16/09 | 1,000,000 | 1a | 29 | | 4902 | | 0.63% | | 0.24 - 3.22% | |
| KNPH3730 | 4/22/09 | 1,300,000 | 1a | 13 | | 4887 | | 0.82% | | 0.12 - 2.01% | |
| ROMI6847 | 9/2/08 | 1,444,000 | 1a | 18 | | 4902 | | 0.94% | | 0.08 - 1.31% | |
| BLMI6862 | 10/3/08 | 1,830,000 | 1a | 22 | | 4880 | | 0.59% | | 0.08 - 2.50% | |
| BRRO6924 | 7/9/09 | 2,400,000 | 1b | 35 | | 4904 | | 0.55% | | 0.08 - 2.96% | |
| ARJA6267 | 3/19/09 | 4,000,000 | 1a | 43 | | 4875 | | 0.64% | | 0.06 - 2.46% | |
| WEPA5774 | 5/11/09 | 6,400,000 | 1a | 44 | | 4902 | | 2.41% | | 0.10 - 3.00% | |
| WHRO3882 | 11/2/05 | 146,000 | 1a | 22 | | 4889 | | 0.33% | | 0.12 - 1.01% | |
| JOTO6422 | 7/31/07 | 2,850,000 | 1a | 21 | | 4887 | | 1.38% | | 0.24 - 2.27% | |
| RUVI5913 | 10/10/08 | 1,870,000 | 1b | 29 | | 4902 | | 1.37% | | 0.14 - 3.00% | |
| LAST90001 | 1/25/11 | 24,000 | 1a | 19 | | 4839 | | 0.23% | | 0.08 - 1.49% | |
| GOTO90002 | 1/25/11 | 2,435,764 | 1b | 20 | | 4842 | | 0.29% | | 0.12 - 0.56% | |
| WIMI90003 | 1/25/11 | 2,081,138 | 1a | 28 | | 4839 | | 0.48% | | 0.14 - 2.17% | |
| Median |  | 2,081,138 |  | 25 | |  | |  | |  | |
| Range |  | 24,000 - 7,690,001 |  | 13-44 | |  | |  | |  | |
