# Supplementary material for: Elucidation of Hepatitis C Virus Transmission and Early Diversification by Single Genome Sequencing
Source: PLoS Pathog. 2012 Aug 23;8(8):e1002880. doi: 10.1371/journal.ppat.1002880 (PMC3426529; doi:10.1371/journal.ppat.1002880)
Supplement: Table S2 — Estimates of numbers of T/F viruses in acute HCV infection using empirical and model based methods. (DOC) [file ppat.1002880.s020.doc]

| **Table S2. Estimates of numbers of T/F viruses in acute HCV infection using empirical and model based methods** | | | | | | | |
| --- | --- | --- | --- | --- | --- | --- | --- |
|  |  |  | **Estimates of numbers of T/F genomes** | | |  |  |
| **Subject** | **Number of sequences** | **Number of time points** | **Empirical^a^** | **Max cut-off model** | **Average cut-off model** | **Power calculation^b^** | **Points of interest** |
| 9055 | 157 | 3 | 1 | 1 | 1 | 1.9% | single founder genome |
| 10021 | 151 | 3 | 1 | 1 | 1 | 2.0% | single founder genome |
| 10025 | 175 | 3 | 1 | 1 | 1 | 1.7% | single founder genome |
| 10051 | 303 | 3 | 1 | 1 | 1 | 1.0% | single founder genome |
| 10003 | 133 | 3 | 37 | 19 | 9 | 2.2% | acute-to-acute transmission |
| 10016 | 72 | 2 | 15 | 11 | 5 | 4.1% | acute-to-acute transmission |
| 10020 | 122 | 3 | 10 | 6 | 2 | 2.4% | acute-to-acute transmission |
| 6213 | 41 | 1 | 3 | 3 | 3 | 7.0% |  |
| 6222 | 17 | 1 | 4 | 4 | 4 | 16.2% |  |
| 10002 | 31 | 2 | 12 | 13 | 11 | 9.2% |  |
| 10004 | 36 | 1 | 3 | 3 | 3 | 8.0% |  |
| 10012 | 230 | 4 | 3 | 3 | 3 | 1.3% |  |
| 10017 | 249 | 5 | 5 | 3 | 3 | 1.2% |  |
| 10024 | 222 | 3 | 6 | 6 | 6 | 1.3% |  |
| 10029 | 322 | 4 | 9 | 9 | 9 | 0.9% |  |
| 10062 | 188 | 4 | 3 | 3 | 3 | 1.6% |  |
| 106889 | 87 | 1 | >30 | 28 | 16 | 3.4% | drug resistant T/F genomes |
| ^a^ Manual estimates were based on phylogenies and *Highlighter* plots of all time points combined. | | | | | | | |
| ^b^ Power calculation estimating an upper bound on the prevalence of unseen variants given the number of sequences analyzed. This estimate is based on the total number of sequences from all time points. | | | | | | | |
