# Supplementary material for: Elucidation of Hepatitis C Virus Transmission and Early Diversification by Single Genome Sequencing
Source: PLoS Pathog. 2012 Aug 23;8(8):e1002880. doi: 10.1371/journal.ppat.1002880 (PMC3426529; doi:10.1371/journal.ppat.1002880)
Supplement: Table S3 — Estimation of numbers of T/F viruses by time point using model-based methods with different cut-offs. (DOC) [file ppat.1002880.s021.doc]

| **Table S3. Estimation of numbers of T/F viruses by time point using model-based methods with different cut-offs** | | | | | | | | |
| --- | --- | --- | --- | --- | --- | --- | --- | --- |
|  |  |  |  |  |  | **Maximum cut-off^b^** | | **Average cut-off^b^** |
| **Subject** | **Sampling time point** | **Genome** | **Number of sequences** | **Sequence length** | **Days since  negative sample** | **Total mutation cut-off^a^** | **Model-based** | **Model-based** |
| 9055 | 7 | 5' half | 44 | 4992 | 6 | 5 | 1 | 1 |
|  | 9 | 5' half | 78 | 4993 | 15 | 5 | 1 | 1 |
|  | 11 | 5' half | 35 | 4993 | 37 | 9 | 1 | 1 |
| 10021 | 8 | 5' quarter 1 | 31 | 2172 | 7 | 3 | 1 | 1 |
|  | 8 | 5' quarter 2 | 24 | 2773 | 7 | 3 | 1 | 1 |
|  | 10 | 5' half | 30 | 4960 | 14 | 5 | 1 | 1 |
|  | 14 | 5' half | 66 | 4960 | 35 | 9 | 1 | 1 |
| 10025 | 8 | 5' quarter 1 | 40 | 2318 | 3 | 3 | 1 | 1 |
|  | 8 | 5' quarter 2 | 43 | 2645 | 3 | 3 | 1 | 1 |
|  | 9 | 5' half | 38 | 4964 | 15 | 5 | 1 | 1 |
|  | 11 | 5' half | 54 | 4964 | 31 | 8 | 1 | 1 |
| 10051 | 9 | 5' quarter 1 | 46 | 2212 | 8 | 3 | 1 | 1 |
|  | 9 | 5' quarter 2 | 45 | 2733 | 8 | 3 | 1 | 1 |
|  | 10 | 5' quarter 1 | 54 | 2212 | 13 | 3 | 1 | 1 |
|  | 10 | 5' quarter 2 | 33 | 2733 | 13 | 3 | 2 | 1 |
|  | 11 | 5' half | 65 | 4948 | 15 | 5 | 1 | 1 |
|  | 14 | 5' half | 60 | 4948 | 27 | 7 | 1 | 1 |
| 10003 | 7 | 5' half | 120 | 4995 | 7 | 5 | 19 | 9 |
|  | 9 | 5' half | 7 | 4995 | 14 | 5 |  |  |
|  | 12 | 5' half | 6 | 4995 | 26 | 7 |  |  |
| 10016 | 10 | 5' quarter | 53 | 2851 | 4 | 3 | 9 | 3 |
|  | 12 | 5' quarter | 19 | 2851 | 28 | 4 | 5 | 3 |
| 10020 | 6 | 5' quarter | 58 | 2194 | 9 | 3 | 5 | 2 |
|  | 8 | 5' half | 24 | 4987 | 16 | 5 | 6 | 1 |
|  | 13 | 5' half | 40 | 4987 | 41 | 10 | 1 | 1 |
| 6213 | 10 | 5' half | 41 | 4905 | 29 | 7 | 3 | 3 |
| 6222 | 8 | 5' half | 17 | 4985 | 38 | 10 | 4 | 4 |
| 10002 | 4 | 5' half | 5 | 4987 | 11 | 5 |  |  |
|  | 7 | 5' half | 26 | 4987 | 24 | 6 | 13 | 11 |
| 10004^c^ | 6 | 5' quarter | 36 | 2849 | 12 | 3 | 3 | 3 |
| 10012^c^ | 6 | 5' quarter 1 | 52 | 2367 | 7 | 3 | 3 | 3 |
|  | 6 | 5' quarter 2 | 49 | 2596 | 7 | 3 | 3 | 3 |
|  | 8 | 5' half | 36 | 4963 | 14 | 5 | 3 | 3 |
|  | 10 | 5' half | 49 | 4963 | 21 | 5 | 3 | 3 |
|  | 13 | 5' half | 44 | 4964 | 33 | 8 | 3 | 3 |
| 10017 | 9 | 5' quarter 1 | 35 | 2288 | 11 | 3 | 3 | 3 |
|  | 9 | 5' quarter 2 | 22 | 2681 | 11 | 3 | 2 | 1 |
|  | 10 | 5' half | 63 | 4970 | 16 | 5 | 3 | 3 |
|  | 12 | 5' half | 48 | 4969 | 24 | 6 | 2 | 2 |
|  | 14 | 5' half | 54 | 4970 | 31 | 8 | 2 | 2 |
|  | 16 | 5' half | 27 | 4970 | 42 | 11 | 1 | 1 |
| 10024 | 6 | 5' quarter 1 | 40 | 2318 | 16 | 3 | 4 | 4 |
|  | 6 | 5' quarter 2 | 70 | 2700 | 16 | 3 | 3 | 3 |
|  | 7 | 5' half | 63 | 4964 | 20 | 5 | 3 | 3 |
|  | 8 | 5' half | 49 | 4964 | 22 | 6 | 3 | 3 |
| 10029 | 8 | 5' quarter 1 | 68 | 2358 | 6 | 3 | 7 | 7 |
|  | 8 | 5' quarter 2 | 53 | 2599 | 6 | 3 | 7 | 6 |
|  | 9 | 5' half | 68 | 4957 | 13 | 5 | 6 | 7 |
|  | 11 | 5' half | 75 | 4957 | 20 | 5 | 7 | 7 |
|  | 15 | 5' half | 58 | 4957 | 34 | 9 | 6 | 5 |
| 10062 | 3 | 5' quarter 1 | 24 | 2425 | 4 | 3 | 2 | 2 |
|  | 3 | 5' quarter 2 | 24 | 2514 | 4 | 3 | 2 | 2 |
|  | 4 | 5' half | 54 | 4938 | 7 | 5 | 3 | 3 |
|  | 5 | 5' half | 59 | 4938 | 11 | 5 | 3 | 3 |
|  | 8 | 5' half | 27 | 4938 | 42 | 11 | 2 | 2 |
| 106889 | 5 | 5' half | 87 | 4984 | 11 | 5 | 28 | 16 |
| ^a^ Total mutation cut-off was based on the time of sampling relative to the last negative time point and the average diversity in a cluster and was used to define distinct founders. | | | | | | | | |
| ^b^ Two methods were used to implement the automated clustering algorithm. The average cut-off method is the more conservative estimate of the minimum number of founder strains needed to explain the observed diversity. The maximum cut-off distinguishes more lineages and separates them into clusters from distinct founders. See methods for details. | | | | | | | | |
| ^c^ The model assumes absence of homoplasy. In these subjects infected by multiple divergent viruses, one mutation at one site could be explained as a homoplasy and hence the corresponding column was removed from the alignment. | | | | | | | | |
