# Supplementary material for: Elucidation of Hepatitis C Virus Transmission and Early Diversification by Single Genome Sequencing
Source: PLoS Pathog. 2012 Aug 23;8(8):e1002880. doi: 10.1371/journal.ppat.1002880 (PMC3426529; doi:10.1371/journal.ppat.1002880)
Supplement: Figure S8 — HCV diversity in acute subject 10017. 5′ quarter 1 genomesequences are color coded in red, orange, green, blue and black in chronological order to reflect sampling time points in Figure 1 and are represented in a ML tree and Highlighter plot. Sequences show evidence of productive clinical infection by at least 4 T/F viruses. Bootstrap values are indicated and represent 100 repetitions. The horizontal scale bar indicates genetic distance. (PDF) [file ppat.1002880.s008.pdf]

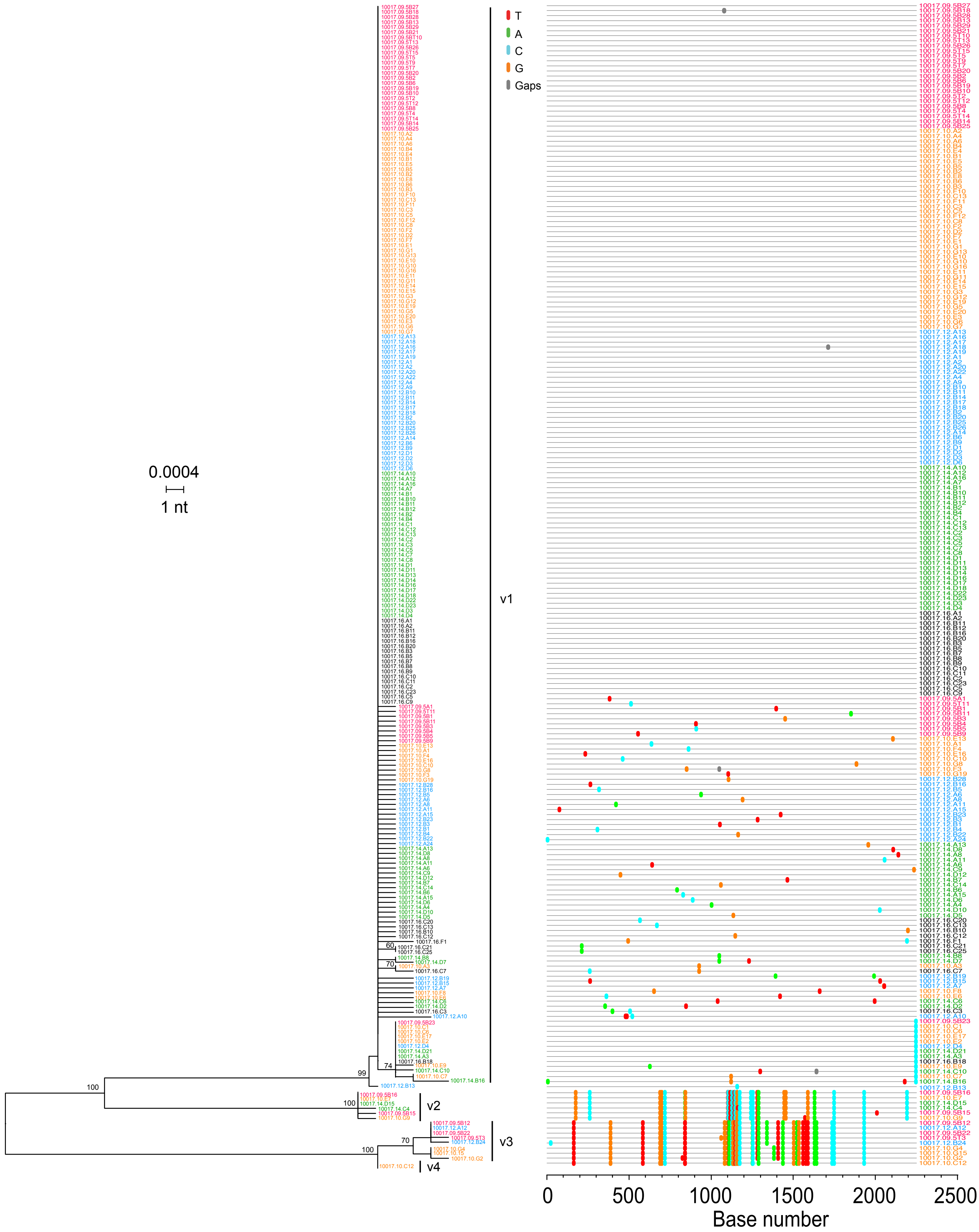
T
A
C
G
Gaps
0.0004
1 nt
v1
v2
v3
v4
100
99
74
70
60
0
500
1000
1500
2000
2500
Base number

Figure S8
