# Supplementary material for: Elucidation of Hepatitis C Virus Transmission and Early Diversification by Single Genome Sequencing
Source: PLoS Pathog. 2012 Aug 23;8(8):e1002880. doi: 10.1371/journal.ppat.1002880 (PMC3426529; doi:10.1371/journal.ppat.1002880)
Supplement: Figure S10 — Nonsynonymous and synonymous mutations in HCV sequences from acute subjects 10003, 10020 and 10016. Highlighter plotsof 5′ half or quarter1 genomesequences are color coded to denote nonynonymous (red) and synonymous (green) mutations for subjects 10003 (panel A), 10020 (panel B) and 10016 (panel C). (PDF) [file ppat.1002880.s010.pdf]

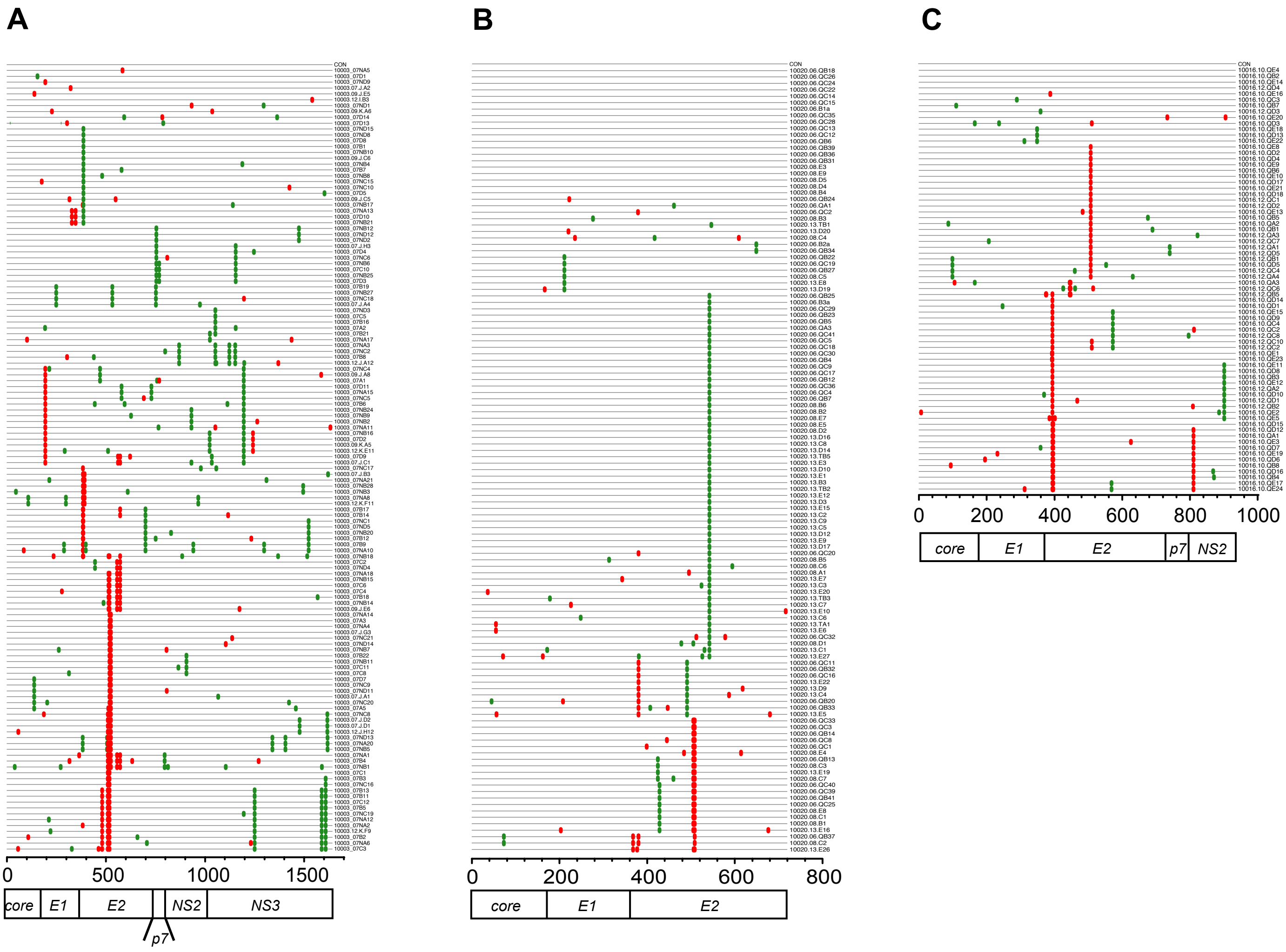
A
B
C
0
500
1000
1500
core
E1
E2
p7
NS2
NS3
0
200
400
600
800
core
E1
E2
0
200
400
600
800
1000
core
E1
E2
p7
NS2

Figure S10
