# Supplementary material for: Elucidation of Hepatitis C Virus Transmission and Early Diversification by Single Genome Sequencing
Source: PLoS Pathog. 2012 Aug 23;8(8):e1002880. doi: 10.1371/journal.ppat.1002880 (PMC3426529; doi:10.1371/journal.ppat.1002880)
Supplement: Figure S12 — Amino acid alignment of the HCV Env coding region of acute subject 10020. The H77 reference sequence is shown at the top. Nonrandom concentrations of nonsynonymous mutations are evident. (PDF) [file ppat.1002880.s012.pdf]

```
                    E2                                                            CD81-1
                      HVR1                                             HVR2                                                                                                                        CD81-2
H77                 ETHVTGGNAG RTTAGLVGLL TPGAKQNIQL INTNGSW....ASCRRLTDFA QGWGPISYAN GSGLDERPYC WHYPPRPCGI VPAKSVCGPV YCFTPSPVVV GTTDRSGAPT YSWGANDTDV FVLNNTRPPL GNWFGCTWMN....
                                                                                                                                     516                              543
10020.06.QB18       KTYVTGGAAA HTTSGLAGLF LQGPKQNVHL INTNGSW....ASCRLLTDFD QGWGPISHAN GSGPDQRPYC WHYPPKPCGI VPAKSVCGPV YCFTPSPVVV GTTDRSGEPT YNWGENDTDV FVLNNIRPPL GNWFGCTWMN....
10020.06.QC26       ---------- ---------- ---------- -------    ---------- ---------- ---------- ---------- ---------- ---------- ---------- ---------- ---------- ----------
10020.06.QC24       ---------- ---------- ---------- -------    ---------- ---------- ---------- ---------- ---------- ---------- ---------- ---------- ---------- ----------
10020.06.QC22       ---------- ---------- ---------- -------    ---------- ---------- ---------- ---------- ---------- ---------- ---------- ---------- ---------- ----------
10020.06.QC14       ---------- ---------- ---------- -------    ---------- ---------- ---------- ---------- ---------- ---------- ---------- ---------- ---------- ----------
10020.06.QC15       ---------- ---------- ---------- -------    ---------- ---------- ---------- ---------- ---------- ---------- ---------- ---------- ---------- ----------
10020.06.B1a        ---------- ---------- ---------- -------    ---------- ---------- ---------- ---------- ---------- ---------- ---------- ---------- ---------- ----------
10020.06.QC35       ---------- ---------- ---------- -------    ---------- ---------- ---------- ---------- ---------- ---------- ---------- ---------- ---------- ----------
10020.06.QC28       ---------- ---------- ---------- -------    ---------- ---------- ---------- ---------- ---------- ---------- ---------- ---------- ---------- ----------
10020.06.QC13       ---------- ---------- ---------- -------    ---------- ---------- ---------- ---------- ---------- ---------- ---------- ---------- ---------- ----------
10020.06.QC12       ---------- ---------- ---------- -------    ---------- ---------- ---------- ---------- ---------- ---------- ---------- ---------- ---------- ----------
10020.06.QB6        ---------- ---------- ---------- -------    ---------- ---------- ---------- ---------- ---------- ---------- ---------- ---------- ---------- ----------
10020.06.QB39       ---------- ---------- ---------- -------    ---------- ---------- ---------- ---------- ---------- ---------- ---------- ---------- ---------- ----------
10020.06.QB36       ---------- ---------- ---------- -------    ---------- ---------- ---------- ---------- ---------- ---------- ---------- ---------- ---------- ----------
10020.06.QB31       ---------- ---------- ---------- -------    ---------- ---------- ---------- ---------- ---------- ---------- ---------- ---------- ---------- ----------
10020.08.E3         ---------- ---------- ---------- -------    ---------- ---------- ---------- ---------- ---------- ---------- ---------- ---------- ---------- ----------
10020.08.E9         ---------- ---------- ---------- -------    ---------- ---------- ---------- ---------- ---------- ---------- ---------- ---------- ---------- ----------
10020.08.D5         ---------- ---------- ---------- -------    ---------- ---------- ---------- ---------- ---------- ---------- ---------- ---------- ---------- ----------
10020.08.D4         ---------- ---------- ---------- -------    ---------- ---------- ---------- ---------- ---------- ---------- ---------- ---------- ---------- ----------
10020.08.B4         ---------- ---------- ---------- -------    ---------- ---------- ---------- ---------- ---------- ---------- ---------- ---------- ---------- ----------
10020.06.QB24       ---------- ---------- ---------- -------    ---------- ---------- ---------- ---------- ---------- ---------- ---------- ---------- ---------- ----------
10020.06.QA1        ---------- ---------- ---------- -------    ---------- ---------- ---------- ---------- ---------- ---------- ---------- ---------- ---------- ----------
10020.06.QC2        ---------- -A-------- ---------- -------    ---------- ---------- ---------- ---------- ---------- ---------- ---------- ---------- ---------- ----------
10020.08.B3         ---------- ---------- ---------- -------    ---------- ---------- ---------- ---------- ---------- ---------- ---------- ---------- ---------- ----------
10020.13.TB1        ---------- ---------- ---------- -------    ---------- ---------- ---------- ---------- ---------- ---------- ---------- ---------- ---------- ----------
10020.13.D20        ---------- ---------- ---------- -------    ---------- ---------- ---------- ---------- ---------- ---------- ---------- ---------- ---------- ----------
10020.08.C4         ---------- ---------- ---------- -------    ---------- ---------- ---------- ---------- ---------- ---------- ---------- ---------- ---------- ----------
10020.06.B2a        ---------- ---------- ---------- -------    ---------- ---------- ---------- ---------- ---------- ---------- ---------- ---------- ---------- ----------
10020.06.QB34       ---------- ---------- ---------- -------    ---------- ---------- ---------- ---------- ---------- ---------- ---------- ---------- ---------- ----------
10020.06.QB22       ---------- ---------- ---------- -------    ---------- ---------- ---------- ---------- ---------- ---------- ---------- ---------- ---------- ----------
10020.06.QC19       ---------- ---------- ---------- -------    ---------- ---------- ---------- ---------- ---------- ---------- ---------- ---------- ---------- ----------
10020.06.QB27       ---------- ---------- ---------- -------    ---------- ---------- ---------- ---------- ---------- ---------- ---------- ---------- ---------- ----------
10020.08.C5         ---------- ---------- ---------- -------    ---------- ---------- ---------- ---------- ---------- ---------- ---------- ---------- ---------- ----------
10020.13.E8         ---------- ---------- ---------- -------    ---------- ---------- ---------- ---------- ---------- ---------- ---------- ---------- ---------- ----------
10020.13.D19        ---------- ---------- ---------- -------    ---------- ---------- ---------- ---------- ---------- ---------- ---------- ---------- ---------- ----------
10020.06.QB25       ---------- ---------- ---------- -------    ---------- ---------- ---------- ---------- ---------- ---------- ---------- ---------- ---------- ----------
10020.06.B3a        ---------- ---------- ---------- -------    ---------- ---------- ---------- ---------- ---------- ---------- ---------- ---------- ---------- ----------
10020.06.QC29       ---------- ---------- ---------- -------    ---------- ---------- ---------- ---------- ---------- ---------- ---------- ---------- ---------- ----------
10020.06.QB23       ---------- ---------- ---------- -------    ---------- ---------- ---------- ---------- ---------- ---------- ---------- ---------- ---------- ----------
10020.06.QB5        ---------- ---------- ---------- -------    ---------- ---------- ---------- ---------- ---------- ---------- ---------- ---------- ---------- ----------
10020.06.QA3        ---------- ---------- ---------- -------    ---------- ---------- ---------- ---------- ---------- ---------- ---------- ---------- ---------- ----------
10020.06.QC41       ---------- ---------- ---------- -------    ---------- ---------- ---------- ---------- ---------- ---------- ---------- ---------- ---------- ----------
10020.06.QC5        ---------- ---------- ---------- -------    ---------- ---------- ---------- ---------- ---------- ---------- ---------- ---------- ---------- ----------
10020.06.QC18       ---------- ---------- ---------- -------    ---------- ---------- ---------- ---------- ---------- ---------- ---------- ---------- ---------- ----------
10020.06.QC30       ---------- ---------- ---------- -------    ---------- ---------- ---------- ---------- ---------- ---------- ---------- ---------- ---------- ----------
10020.06.QB4        ---------- ---------- ---------- -------    ---------- ---------- ---------- ---------- ---------- ---------- ---------- ---------- ---------- ----------
10020.06.QC9        ---------- ---------- ---------- -------    ---------- ---------- ---------- ---------- ---------- ---------- ---------- ---------- ---------- ----------
10020.06.QC17       ---------- ---------- ---------- -------    ---------- ---------- ---------- ---------- ---------- ---------- ---------- ---------- ---------- ----------
10020.06.QB12       ---------- ---------- ---------- -------    ---------- ---------- ---------- ---------- ---------- ---------- ---------- ---------- ---------- ----------
10020.06.QC36       ---------- ---------- ---------- -------    ---------- ---------- ---------- ---------- ---------- ---------- ---------- ---------- ---------- ----------
10020.06.QC4        ---------- ---------- ---------- -------    ---------- ---------- ---------- ---------- ---------- ---------- ---------- ---------- ---------- ----------
10020.06.QB7        ---------- ---------- ---------- -------    ---------- ---------- ---------- ---------- ---------- ---------- ---------- ---------- ---------- ----------
10020.08.B6         ---------- ---------- ---------- -------    ---------- ---------- ---------- ---------- ---------- ---------- ---------- ---------- ---------- ----------
10020.08.B2         ---------- ---------- ---------- -------    ---------- ---------- ---------- ---------- ---------- ---------- ---------- ---------- ---------- ----------
10020.08.E7         ---------- ---------- ---------- -------    ---------- ---------- ---------- ---------- ---------- ---------- ---------- ---------- ---------- ----------
10020.08.E5         ---------- ---------- ---------- -------    ---------- ---------- ---------- ---------- ---------- ---------- ---------- ---------- ---------- ----------
10020.08.D2         ---------- ---------- ---------- -------    ---------- ---------- ---------- ---------- ---------- ---------- ---------- ---------- ---------- ----------
10020.13.D16        ---------- ---------- ---------- -------    ---------- ---------- ---------- ---------- ---------- ---------- ---------- ---------- ---------- ----------
10020.13.C8         ---------- ---------- ---------- -------    ---------- ---------- ---------- ---------- ---------- ---------- ---------- ---------- ---------- ----------
10020.13.D14        ---------- ---------- ---------- -------    ---------- ---------- ---------- ---------- ---------- ---------- ---------- ---------- ---------- ----------
10020.13.TB5        ---------- ---------- ---------- -------    ---------- ---------- ---------- ---------- ---------- ---------- ---------- ---------- ---------- ----------
10020.13.E3         ---------- ---------- ---------- -------    ---------- ---------- ---------- ---------- ---------- ---------- ---------- ---------- ---------- ----------
10020.13.D10        ---------- ---------- ---------- -------    ---------- ---------- ---------- ---------- ---------- ---------- ---------- ---------- ---------- ----------
10020.13.E1         ---------- ---------- ---------- -------    ---------- ---------- ---------- ---------- ---------- ---------- ---------- ---------- ---------- ----------
10020.13.B3         ---------- ---------- ---------- -------    ---------- ---------- ---------- ---------- ---------- ---------- ---------- ---------- ---------- ----------
10020.13.TB2        ---------- ---------- ---------- -------    ---------- ---------- ---------- ---------- ---------- ---------- ---------- ---------- ---------- ----------
10020.13.E12        ---------- ---------- ---------- -------    ---------- ---------- ---------- ---------- ---------- ---------- ---------- ---------- ---------- ----------
10020.13.D3         ---------- ---------- ---------- -------    ---------- ---------- ---------- ---------- ---------- ---------- ---------- ---------- ---------- ----------
10020.13.E15        ---------- ---------- ---------- -------    ---------- ---------- ---------- ---------- ---------- ---------- ---------- ---------- ---------- ----------
10020.13.C2         ---------- ---------- ---------- -------    ---------- ---------- ---------- ---------- ---------- ---------- ---------- ---------- ---------- ----------
10020.13.C9         ---------- ---------- ---------- -------    ---------- ---------- ---------- ---------- ---------- ---------- ---------- ---------- ---------- ----------
10020.13.C5         ---------- ---------- ---------- -------    ---------- ---------- ---------- ---------- ---------- ---------- ---------- ---------- ---------- ----------
10020.13.D12        ---------- ---------- ---------- -------    ---------- ---------- ---------- ---------- ---------- ---------- ---------- ---------- ---------- ----------
10020.13.E9         ---------- ---------- ---------- -------    ---------- ---------- ---------- ---------- ---------- ---------- ---------- ---------- ---------- ----------
10020.13.D17        ---------- ---------- ---------- -------    ---------- ---------- ---------- ---------- ---------- ---------- ---------- ---------- ---------- ----------
10020.06.QC20       ---------- --A------- ---------- -------    ---------- ---------- ---------- ---------- ---------- ---------- ---------- ---------- ---------- ----------
10020.08.B5         ---------- ---------- ---------- -------    ---------- ---------- ---------- ---------- ---------- ---------- ---------- ---------- ---------- ----------
10020.08.C6         ---------- ---------- ---------- -------    ---------- ---------- ---------- ---------- ---------- ---------- ---------- ---------- ---------- ----------
10020.08.A1         ---------- ---------- ---------- -------    ---------- ---------- ---------- ---------- ---------- ----L----- ---------- ---------- ---------- ----------
10020.13.E7         ---------- ---------- ---------- -------    ---------- ---------- ---------- ---------- ---------- ---------- ---------- ---------- ---------- ----------
10020.13.C3         ---------- ---------- ---------- -------    ---------- ---------- ---------- ---------- ---------- ---------- ---------- ---------- ---------- ----------
10020.13.E20        ---------- ---------- ---------- -------    ---------- ---------- ---------- ---------- ---------- ---------- ---------- ---------- ---------- ----------
10020.13.TB3        ---------- ---------- ---------- -------    ---------- ---------- ---------- ---------- ---------- ---------- ---------- ---------- ---------- ----------
10020.13.C7         ---------- ---------- ---------- -------    ---------- ---------- ---------- ---------- ---------- ---------- ---------- ---------- ---------- ----------
10020.13.E10        ---------- ---------- ---------- -------    ---------- ---------- ---------- ---------- ---------- ---------- ---------- ---------- ---------- ----------
10020.13.C6         ---------- ---------- ---------- -------    ---------- ---------- ---------- ---------- ---------- ---------- ---------- ---------- ---------- ----------
10020.13.TA1        ---------- ---------- ---------- -------    ---------- ---------- ---------- ---------- ---------- ---------- ---------- ---------- ---------- ----------
10020.13.E6         ---------- ---------- ---------- -------    ---------- ---------- ---------- ---------- ---------- ---------- ---------- ---------- ---------- ----------
10020.06.QC32       ---------- ---------- ---------- -------    ---------- ---------- ---------- ---------- ---------- ---------- ---------- -Y-------- ---------- ----------
10020.08.D1         ---------- ---------- ---------- -------    ---------- ---------- ---------- ---------- ---------- ---------- ---------- ---------- ---------- ----------
10020.13.C1         ---------- ---------- ---------- -------    ---------- ---------- ---------- ---------- ---------- ---------- ---------- ---------- ---------- ----------
10020.13.E27        ---------- ---------- ---------- -------    ---------- ---------- ---------- ---------- ---------- ---------- ---------- ---------- ---------- ----------
10020.06.QC11       ---------- --A------- ---------- -------    ---------- ---------- ---------- ---------- ---------- ---------- ---------- ---------- ---------- ----------
10020.06.QB32       ---------- --A------- ---------- -------    ---------- ---------- ---------- ---------- ---------- ---------- ---------- ---------- ---------- ----------
10020.06.QC16       ---------- --A------- ---------- -------    ---------- ---------- ---------- ---------- ---------- ---------- ---------- ---------- ---------- ----------
10020.13.E22        ---------- --A------- ---------- -------    ---------- ---------- ---------- ---------- ---------- ---------- ---------- ---------- ---------- ----------
10020.13.D9         ---------- --A------- ---------- -------    ---------- ---------- ---------- ---------- ---------- ---------- ---------- ---------- ---------- ----------
10020.13.C4         ---------- --A------- ---------- -------    ---------- ---------- ---------- ---------- ---------- ---------- ---------- ---------- ---------- ----------
10020.06.QB20       ---------- --A------- ---------- -------    ---------- ---------- ---------- ---------- ---------- ---------- ---------- ---------- ---------- ----------
10020.06.QB33       ---------- --A------- ---------- -------    ------I--- ---------- ---------- ---------- ---------- ---------- ---------- ---------- ---------- ----------
10020.13.E5         ---------- --A------- ---------- -------    ---------- ---------- ---------- ---------- ---------- ---------- ---------- ---------- ---------- ----------
10020.06.QC33       ---------- ---------- ---------- -------    ---------- ---------- ---------- ---------- ---------- ---------- ----G--V-- ---------- ---------- ----------
10020.06.QC3        ---------- ---------- ---------- -------    ---------- ---------- ---------- ---------- ---------- ---------- ----G--V-- ---------- ---------- ----------
10020.06.QB14       ---------- ---------- ---------- -------    ---------- ---------- ---------- ---------- ---------- ---------- ----G--V-- ---------- ---------- ----------
10020.06.QC8        ---------- ---------- ---------- -------    ----P----- ---------- ---------- ---------- ---------- ---------- ----G--V-- ---------- ---------- ----------
10020.06.QC1        ---------- ---------- ---------- -S-----    ---------- ---------- ---------- ---------- ---------- ---------- ----G--V-- ---------- ---------- ----------
10020.08.E4         ---------- ---------- ---------- -------    ---------- ---------- ---------- ---------- ---R------ ---------- ----G--V-- ---------- ---------- ----------
10020.06.QB13       ---------- ---------- ---------- -------    ---------- ---------- ---------- ---------- ---------- ---------- ----G--V-- ---------- ---------- ----------
10020.08.C3         ---------- ---------- ---------- -------    ---------- ---------- ---------- ---------- ---------- ---------- ----G--V-- ---------- ---------- ----------
10020.13.E19        ---------- ---------- ---------- -------    ---------- ---------- ---------- ---------- ---------- ---------- ----G--V-- ---------- ---------- ----------
10020.08.C7         ---------- ---------- ---------- -------    ---------- ---------- ---------- ---------- ---------- ---------- ----G--V-- ---------- ---------- ----------
10020.06.QC40       ---------- ---------- ---------- -------    ---------- ---------- ---------- ---------- ---------- ---------- ----G--V-- ---------- ---------- ----------
10020.06.QC39       ---------- ---------- ---------- -------    ---------- ---------- ---------- ---------- ---------- ---------- ----G--V-- ---------- ---------- ----------
10020.06.QB41       ---------- ---------- ---------- -------    ---------- ---------- ---------- ---------- ---------- ---------- ----G--V-- ---------- ---------- ----------
10020.06.QC25       ---------- ---------- ---------- -------    ---------- ---------- ---------- ---------- ---------- ---------- ----G--V-- ---------- ---------- ----------
10020.08.E8         ---------- ---------- ---------- -------    ---------- ---------- ---------- ---------- ---------- ---------- ----G--V-- ---------- ---------- ----------
10020.08.C1         ---------- ---------- ---------- -------    ---------- ---------- ---------- ---------- ---------- ---------- ----G--V-- ---------- ---------- ----------
10020.08.B1         ---------- ---------- ---------- -------    ---------- ---------- ---------- ---------- ---------- ---------- ----G--V-- ---------- ---------- ----------
10020.13.E16        ---------- ---------- ---------- -------    ---------- ---------- ---------- ---------- ---------- ---------- ----G--V-- ---------- ---------- ----------
10020.06.QB37       G--------- --A------- ---------- -------    ---------- ---------- ---------- ---------- ---------- ---------- -------V-- ---------- ---------- ----------
10020.08.C2         G--------- --A------- ---------- -------    ---------- ---------- ---------- ---------- ---------- ---------- -------V-- ---------- ---------- ----------
10020.13.E26        E--------G ---------- ---------- -------    ---------- ---------- ---------- ---------- ---------- ---------- -----A-V-- ---------- ---------- ----------
```

Figure S12
