# Supplementary material for: Elucidation of Hepatitis C Virus Transmission and Early Diversification by Single Genome Sequencing
Source: PLoS Pathog. 2012 Aug 23;8(8):e1002880. doi: 10.1371/journal.ppat.1002880 (PMC3426529; doi:10.1371/journal.ppat.1002880)
Supplement: Figure S13 — Amino acid alignment of the HCV Env coding region of acute subject 10016. The H77 sequence is shown at the top. Nonrandom concentrations of nonsynonymous mutations are evident. (PDF) [file ppat.1002880.s013.pdf]

```
                 E2
                 HVR1                                                    HVR2       CD81-1                                                                                  CD81-2
H77              ETHVTGGNAG RTTAGLVGLL TPGAKQNIQL INTNGSW....ASCRRLTDFA QGWGPISYAN GSGLDE--RP YCWHYPPRPC GIVPAKSVCG PVYCFTPSPV VVGTTDRSGA PTYSWGANDT DVFVLNNTRP PLGNWFGCTW MN....
                                                                                                                                   516                    543
10016.10.QE4     ETHVTGGQAG RTTAGFASLF SSGAQQEISL INTNGSW....SSCRRLEDFR IGWGTLEYET NVTNDEDMRP YCWHYPPKPC GIVSAKTVCG PVYCFTPSPV VVGTTDKQGA PTYSWGENET DVFLLNSTRP PQGAWFGCTW MN....
10016.10.QB2     ---------- ---------- ---------- -------    ---------- ---------- ---------- ---------- ---------- ---------- ---------- ---------- ---------- ---------- --
10016.10.QE14    ---------- ---------- ---------- -------    ---------- ---------- ---------- ---------- ---------- ---------- ---------- ---------- ---------- ---------- --
10016.12.QD4     ---------- ---------- ---------- -------    ---------- ---------- ---------- ---------- ---------- ---------- ---------- ---------- ---------- ---------- --
10016.10.QE16    ---------- ---------- G--------- -------    ---------- ---------- ---------- ---------- ---------- ---------- ---------- ---------- ---------- ---------- --
10016.10.QC3     ---------- ---------- ---------- -------    ---------- ---------- ---------- ---------- ---------- ---------- ---------- ---------- ---------- ---------- --
10016.10.QB7     ---------- ---------- ---------- -------    ---------- ---------- ---------- ---------- ---------- ---------- ---------- ---------- ---------- ---------- --
10016.12.QD3     ---------- ---------- ---------- -------    ---------- ---------- ---------- ---------- ---------- ---------- ---------- ---------- ---------- ---------- --
10016.10.QE20    ---------- ---------- ---------- -------    ---------- ---------- ---------- ---------- ---------- ---------- ---------- ---------- ---------- ---------- --
10016.10.QD3     ---------- ---------- ---------- -------    ---------- ---------- ---------- ---------- ---------- ---------- ---------V ---------- ---------- ---------- --
10016.10.QE18    ---------- ---------- ---------- -------    ---------- ---------- ---------- ---------- ---------- ---------- ---------- ---------- ---------- ---------- --
10016.10.QD13    ---------- ---------- ---------- -------    ---------- ---------- ---------- ---------- ---------- ---------- ---------- ---------- ---------- ---------- --
10016.10.QE22    ---------- ---------- ---------- -------    ---------- ---------- ---------- ---------- ---------- ---------- ---------- ---------- ---------- ---------- --
10016.10.QE8     ---------- ---------- ---------- -------    ---------- ---------- ---------- ---------- ---------- ---------- ------R--- ---------- ---------- ---------- --
10016.10.QD2     ---------- ---------- ---------- -------    ---------- ---------- ---------- ---------- ---------- ---------- ------R--- ---------- ---------- ---------- --
10016.10.QD4     ---------- ---------- ---------- -------    ---------- ---------- ---------- ---------- ---------- ---------- ------R--- ---------- ---------- ---------- --
10016.10.QE9     ---------- ---------- ---------- -------    ---------- ---------- ---------- ---------- ---------- ---------- ------R--- ---------- ---------- ---------- --
10016.10.QB6     ---------- ---------- ---------- -------    ---------- ---------- ---------- ---------- ---------- ---------- ------R--- ---------- ---------- ---------- --
10016.10.QE10    ---------- ---------- ---------- -------    ---------- ---------- ---------- ---------- ---------- ---------- ------R--- ---------- ---------- ---------- --
10016.10.QD17    ---------- ---------- ---------- -------    ---------- ---------- ---------- ---------- ---------- ---------- ------R--- ---------- ---------- ---------- --
10016.10.QE21    ---------- ---------- ---------- -------    ---------- ---------- ---------- ---------- ---------- ---------- ------R--- ---------- ---------- ---------- --
10016.10.QD18    ---------- ---------- ---------- -------    ---------- ---------- ---------- ---------- ---------- ---------- ------R--- ---------- ---------- ---------- --
10016.12.QC1     ---------- ---------- ---------- -------    ---------- ---------- ---------- ---------- ---------- ---------- ------R--- ---------- ---------- ---------- --
10016.12.QD2     ---------- ---------- ---------- -------    ---------- ---------- ---------- ---------- ---------- ---------- ------R--- ---------- ---------- ---------- --
10016.10.QE13    ---------- ---------- ---------- -------    ---------- ---------- ---------- ---------- --I------- ---------- ------R--- ---------- ---------- ---------- --
10016.10.QB5     ---------- ---------- ---------- -------    ---------- ---------- ---------- ---------- ---------- ---------- ------R--- ---------- ---------- ---------- --
10016.10.QA2     ---------- ---------- ---------- -------    ---------- ---------- ---------- ---------- ---------- ---------- ------R--- ---------- ---------- ---------- --
10016.10.QB1     ---------- ---------- ---------- -------    ---------- ---------- ---------- ---------- ---------- ---------- ------R--- ---------- ---------- ---------- --
10016.12.QA3     ---------- ---------- ---------- -------    ---------- ---------- ---------- ---------- ---------- ---------- ------R--- ---------- ---------- ---------- --
10016.12.QC7     ---------- ---------- ---------- -------    ---------- ---------- ---------- ---------- ---------- ---------- ------R--- ---------- ---------- ---------- --
10016.12.QA1     ---------- ---------- ---------- -------    ---------- ---------- ---------- ---------- ---------- ---------- ------R--- ---------- ---------- ---------- --
10016.12.QD5     ---------- ---------- ---------- -------    ---------- ---------- ---------- ---------- ---------- ---------- ------R--- ---------- ---------- ---------- --
10016.12.QB1     ---------- ---------- ---------- -------    ---------- ---------- ---------- ---------- ---------- ---------- ------R--- ---------- ---------- ---------- --
10016.10.QD5     ---------- ---------- ---------- -------    ---------- ---------- ---------- ---------- ---------- ---------- ------R--- ---------- ---------- ---------- --
10016.12.QC4     ---------- ---------- ---------- -------    ---------- ---------- ---------- ---------- ---------- ---------- ------R--- ---------- ---------- ---------- --
10016.12.QA4     ---------- ---------- ---------- -------    ---------- ---------- ---------- ---------- ---------- ---------- ------R--- ---------- ---------- ---------- --
10016.10.QA3     ---------- ---------- ---------- -------    ----S-D--- ---------- ---------- ---------- ---------- ---------- ---------- ---------- ---------- ---------- --
10016.12.QC6     ---------- ---------- ---------- -------    ----T-D--- ---------- ---------- ---------- ---------- ---------- ---------- ---N------ ---------- ---------- --
10016.12.QB5     -------LV- ---------- ------R--- -------    ----T-D--- ---------- ---------- ---------- ---------- ---------- ---------- ---------- ---------- ---------- --
10016.10.QD14    ---------- ---------- ------Q--- -------    ---------- ---------- ---------- ---------- ---------- ---------- ---------- ---------- ---------- ---------- --
10016.10.QD1     ---------- ---------- ------Q--- -------    ---------- ---------- ---------- ---------- ---------- ---------- ---------- ---------- ---------- ---------- --
10016.10.QE15    ---------- ---------- ------Q--- -------    ---------- ---------- ---------- ---------- ---------- ---------- ---------- ---------- ---------- ---------- --
10016.10.QD9     ---------- ---------- ------Q--- -------    ---------- ---------- ---------- ---------- ---------- ---------- ---------- ---------- ---------- ---------- --
10016.10.QC4     ---------- ---------- ------Q--- -------    ---------- ---------- ---------- ---------- ---------- ---------- ---------- ---------- ---------- ---------- --
10016.10.QC2     ---------- ---------- ------Q--- -------    ---------- ---------- ---------- ---------- ---------- ---------- ---------- ---------- ---------- ---------- --
10016.12.QC8     ---------- ---------- ------Q--- -------    ---------- ---------- ---------- ---------- ---------- ---------- ---------- ---------- ---------- ---------- --
10016.12.QC10    ---------- ---------- ------Q--- -------    ---------- ---------- ---------- ---------- ---------- ---------- ---------V ---------- ---------- ---------- --
10016.12.QC2     ---------- ---------- ------Q--- -------    ---------- ---------- ---------- ---------- ---------- ---------- ---------V ---------- ---------- ---------- --
10016.10.QE1     ---------- ---------- ----R-K--- -------    ---------- ---------- ---------- ---------- ---------- ---------- ---------- ---------- ---------- ---------- --
10016.10.QE23    ---------- ---------- ----R-K--- -------    ---------- ---------- ---------- ---------- ---------- ---------- ---------- ---------- ---------- ---------- --
10016.10.QE11    ---------- ---------- ------R--- -------    ---------- ---------- ---------- ---------- ---------- ---------- ---------- ---------- ---------- ---------- --
10016.10.QD8     ---------- ---------- ------R--- -------    ---------- ---------- ---------- ---------- ---------- ---------- ---------- ---------- ---------- ---------- --
10016.10.QB3     ---------- ---------- ------R--- -------    ---------- ---------- ---------- ---------- ---------- ---------- ---------- ---------- ---------- ---------- --
10016.10.QE12    ---------- ---------- ------R--- -------    ---------- ---------- ---------- ---------- ---------- ---------- ---------- ---------- ---------- ---------- --
10016.12.QA2     ---------- ---------- ------R--- -------    ---------- ---------- ---------- ---------- ---------- ---------- ---------- ---------- ---------- ---------- --
10016.10.QD10    ---------- ---------- ------R--- -------    ---------- ---------- ---------- ---------- ---------- ---------- ---------- ---------- ---------- ---------- --
10016.12.QD1     ---------- ---------- ------R--- -------    ---------- ---------- ------G--- ---------- ---------- ---------- ---------- ---------- ---------- ---------- --
10016.12.QB2     ---------- ---------- ------R--- -------    ---------- ---------- ---------- ---------- ---------- ---------- ---------- ---------- ---------- ---------- --
10016.10.QE2     ---------- ---------- ------R--- -------    ---------- ---------- ---------- ---------- ---------- ---------- ---------- ---------- ---------- ---------- --
10016.10.QE5     ---------- -------T-- ------R--- ---D---    ---------- ---------- ---------- ---------- ---------- ---------- ---------- ---------- ---------- ---------- --
10016.10.QD15    ---------- ---------- ------K-N- -------    ---------- ---------- ---------- ---------- ---------- ---------- ---------- ---------- ---------- ---------- --
10016.10.QD12    ---------- ---------- ------K-N- -------    ---------- ---------- ---------- ---------- ---------- ---------- ---------- ---------- ---------- ---------- --
10016.10.QA1     ---------- ---------- ------K-N- -------    ---------- ---------- ---------- ---------- ---------- ---------- ---------- ---------- ---------- ---------- --
10016.10.QE3     ---------- ---------- ------K-N- -------    ---------- ---------- ---------- ---------- ---------- ---------- ---------- ---------- ---------- ---------- --
10016.10.QD7     ---------- ---------- ------K-N- -------    ---------- ---------- ---------- ---------- ---------- ---------- ---------- ---------- ---------- ---------- --
10016.10.QE19    ---------- ---------- ------K-N- -------    ---------- ---------- ---------- ---------- ---------- ---------- ---------- ---------- ---------- ---------- --
10016.10.QD6     ---------- ---------- ------K-N- -------    ---------- ---------- ---------- ---------- ---------- ---------- ---------- ---------- ---------- ---------- --
10016.10.QB8     ---------- ---------- ------K-N- -------    ---------- ---------- ---------- ---------- ---------- ---------- ---------- ---------- ---------- ---------- --
10016.10.QD16    ---------- ---------- ------K-N- -------    ---------- ---------- ---------- ---------- ---------- ---------- ---------- ---------- ---------- ---------- --
10016.10.QB4     ---------- ---------- ------K-N- -------    ---------- ---------- ---------- ---------- ---------- ---------- ---------- ---------- ---------- ---------- --
10016.10.QE17    ---------- ---------- ------K-N- -------    ---------- ---------- ---------- ---------- ---------- ---------- ---------- ---------- ---------- ---------- --
10016.10.QE24    ---------- ---------- ------K-N- -------    ---------- ---------- ---------- ---------- ---------- ---------- ---------- ---------- ---------- ---------- --
```

Figure S13
