# Supplementary material for: Elucidation of Hepatitis C Virus Transmission and Early Diversification by Single Genome Sequencing
Source: PLoS Pathog. 2012 Aug 23;8(8):e1002880. doi: 10.1371/journal.ppat.1002880 (PMC3426529; doi:10.1371/journal.ppat.1002880)
Supplement: Figure S16 — HCV diversity in acute subject 9055. 5′ half genomesequences are color coded in green, blue and black in chronological order to reflect sampling time points in Figure 1 and are represented in a ML tree and Highlighter plot. Sequences show evidence of productive clinical infection by one T/F virus with mutations accumulating at positions 3311 and 3346 at the last sampling time point coincident with antibody seroconversion. Bootstrap values represent 100 repetitions. The horizontal scale bar indicates genetic distance. (PDF) [file ppat.1002880.s016.pdf]

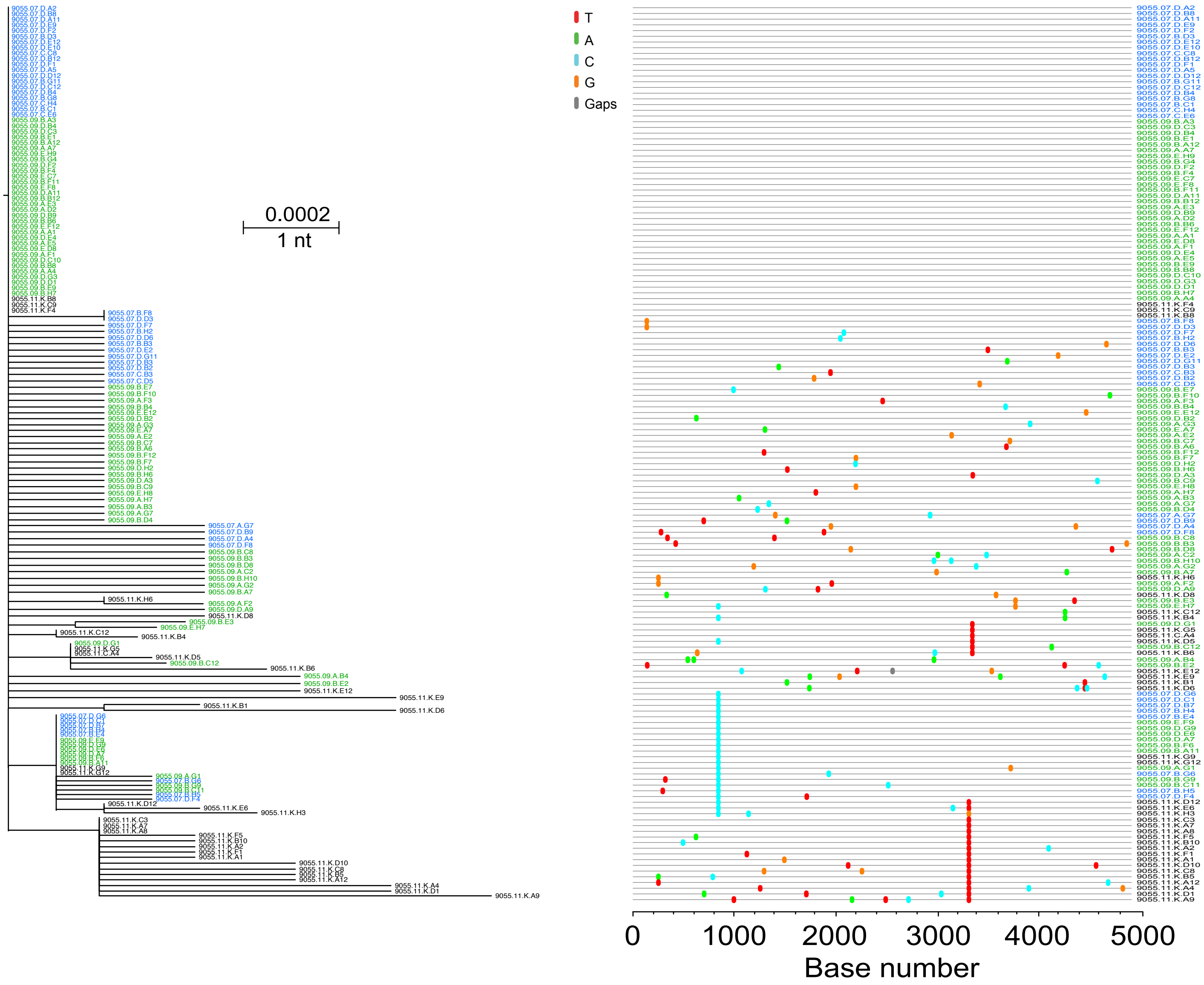

Figure S16
